# Synthesis and Evaluation of Polymyxins Bearing Reductively Labile Disulfide-Linked Lipids

**DOI:** 10.1021/acs.jmedchem.2c01528

**Published:** 2022-11-18

**Authors:** Cornelis J. Slingerland, Charlotte M. J. Wesseling, Paolo Innocenti, Koen G. C. Westphal, Rosalinde Masereeuw, Nathaniel I. Martin

**Affiliations:** † Biological Chemistry Group, Institute of Biology Leiden, Leiden University, Sylviusweg 72, 2333 BE Leiden, The Netherlands; ‡ Division of Pharmacology, Utrecht Institute for Pharmaceutical Sciences, Utrecht University, 3584 CG Utrecht, The Netherlands

## Abstract

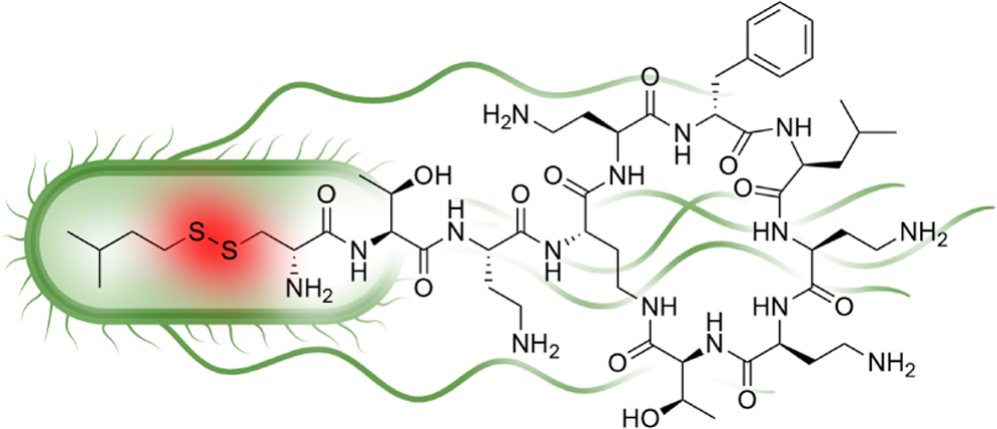

Polymyxins are a class of lipopeptide anti-infective agents with potent and specific activity against Gram-negative bacteria. While toxicity concerns associated with polymyxin B and E (colistin) have historically limited their clinical application, today they are increasingly used as last-resort antibiotics given the rise of multidrug-resistant Gram-negative pathogens. The adverse side effects of polymyxins are well known, particularly as related to their nephrotoxicity. Here, we describe the synthesis and evaluation of a novel series of polymyxin analogues, aimed at reducing their nephrotoxic effects. Using a semisynthetic approach, we explored modifications of the exocyclic part of the polymyxin scaffold, namely, the terminal amino acid and lipophilic tail. By incorporating a reductively labile disulfide linkage in the lipid tail, we obtained novel polymyxins that exhibit potent antibacterial activity on par with polymyxin B but with reduced toxicity toward human renal proximal tubular epithelial cells.

## Introduction

Worldwide, the emergence of multidrug-resistant bacteria is on the rise while the pipeline of new antibiotics under development is nearly dry. This problem is most notable when it comes to the dearth of new antibiotics that target Gram-negative species. In this regard, the World Health Organization recently listed carbapenem-resistant *Acinetobacter baumannii*, *Pseudomonas aeruginosa*, and *Enterobacteriaceae* as pathogens for which new antibiotics are critically needed.^1^ To address this need, a number of approaches can be considered, including: (1) identifying and developing entirely new antibiotics with novel mechanisms of action, (2) looking for synergistic combinations of approved drugs, or (3) improving on already approved antibiotics by structural modification. While the first approach offers perhaps the best chance to address resistance, it is also inherently the most challenging. Alternatively, while combination therapies can show promising effects in *in vitro* assays, their applicability *in vivo* can be hampered by the different pharmacokinetic (PK) and pharmacodynamic (PD) properties of the individual components. By comparison, pursuing improved structural analogues of known antibiotics as a means of enhancing activity and/or overcoming resistance or reducing toxicity has historically delivered many clinical successes and remains an important and fruitful strategy.

**Figure 1:**
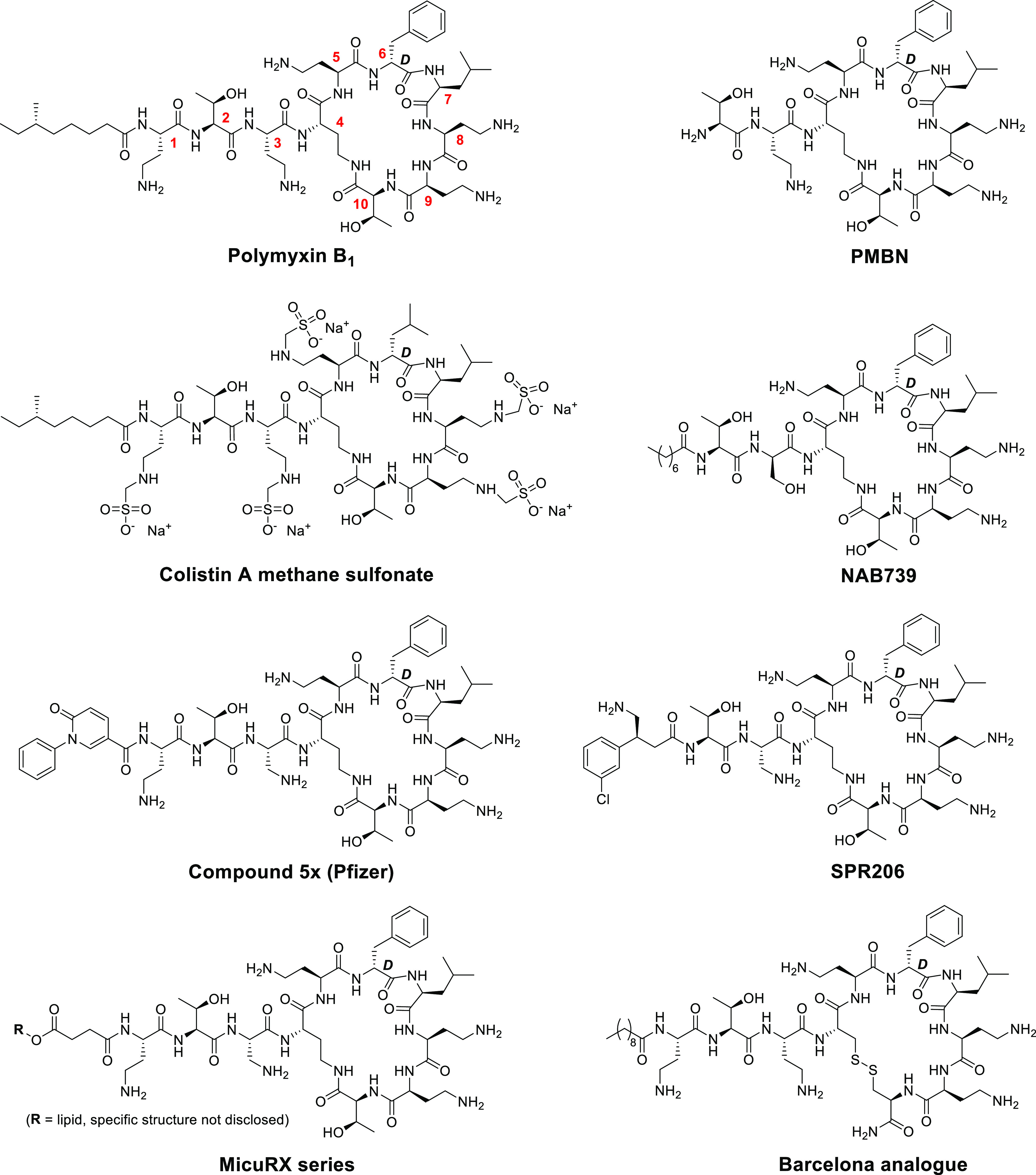
Structures of polymyxins and polymyxin-derived analogues. Typical numbering used for polymyxin residues is indicated in the structure for the natural product polymyxin B_1_. PMBN: polymyxin B nonapeptide.

The polymyxins (as exemplified by polymyxin B_1_, Figure 1) are a well-established class of anti-Gram-negative antibiotics, first isolated from *Bacillus polymyxa* in the late 1940s.^2^ Since their initial discovery, many different polymyxin variants have been isolated from natural sources.^3−7^ Most extensively studied are the clinically used members of the family, polymyxin B and polymyxin E (commonly called colistin). The component amino acids are typically numbered as shown for polymyxin B_1_ in Figure 1. Polymyxin B and colistin differ only in the residue found at P6, which is d-Phe in polymyxin B and d-Leu in colistin. Structurally, polymyxins contain a macrocyclic heptapeptide, formed via an amide linkage between the C-terminus of the peptide and the side chain of the 2,4-diaminobutyric acid (Dab) residue found at P4, along with an exocyclic tripeptide that is acylated at the N-terminus with a fatty acid tail. The presence of five Dab residues gives the polymyxins a high net positive charge at physiological pH. The main target of the polymyxins is the lipopolysaccharide (LPS) portion of the bacterial outer membrane (OM), which explains their Gram-negative specific activity. Despite decades of research, the precise mechanism of action by which the polymyxins elicit their antibacterial effect is still an active area of investigation.^8−10^

It is hypothesized that the positively charged side chains of the five Dab residues allow for electrostatic interactions with the phosphate groups of lipid A, the lipid anchor of LPS.^8^ Binding studies also indicate that the interaction of polymyxins with LPS is affected by the type of LPS (either rough or smooth) used, suggesting that (modified) liposaccharides beyond lipid A may also influence binding.^11^ In addition to electrostatic interactions, hydrophobic contacts between the polymyxin’s fatty acyl tail as well as the hydrophobic amino acids at P6/P7 with the outer and inner membranes of susceptible bacteria also play a key role.^8^ Furthermore, it has been shown that membrane fluidization and lipid A aggregation are both observed upon exposure of bacterial cells to polymyxins.^11^ Additional mechanistic insights have come from studies with polymyxin nonapeptide (PMBN, Figure 1), a polymyxin B derivative devoid of the alkyl chain and N-terminal Dab residue. While PMBN is able to disrupt the Gram-negative OM and, in doing so, effectively synergizes with different antibiotics,^12−15^ it has no direct antimicrobial effects on its own. This observation clearly indicates the necessity of the lipid tail for the activity of the polymyxins.

Like other membrane-active cationic antimicrobial peptides, polymyxins selectively target bacterial membranes over mammalian membranes. Both charge and secondary structure are believed to be important contributors to this selectivity.^16^ Notably, while polymyxins are essentially nonhemolytic, their clinical application is dose-limited due to their well-documented nephrotoxicity, an effect that has historically limited their widespread use in treating infections.^17,18^ However, with the increasing incidence of multidrug-resistant Gram-negative pathogens, the use of polymyxin therapy is on the rise.^19^ In this regard, polymyxin analogues that maintain the potent antibacterial activity of natural products while exhibiting reduced toxicity are highly desirable.

The molecular basis of polymyxin toxicity is not completely understood. It is known, however, that PMBN is significantly less toxic than the parent compound polymyxin B.^20,21^ This indicates that the hydrophobic lipid tail together with the Dab residue at P1 of polymyxin B plays a key role in damaging renal tissue. At the cellular level, polymyxins appear to accumulate heavily in the proximal tubular cells,^22^ for which entry is facilitated by megalin,^23,24^ an endocytic receptor involved in nutrient and polybasic drug uptake.^25,26^ Excessive polymyxin accumulation via this resorption mechanism in turn interferes with cellular functions, causing either apoptosis^27^ or necrosis.^28^

To date, a number of approaches have been explored as a means of accessing less toxic polymyxin analogues.^7,29−31^ Administering colistin in the form of a colistin methane sulfonate (Figure 1) prodrug was explored early on,^32^ but the efficacy of this approach has been recently called into question.^18^ Given the central role of megalin and its affinity toward polybasic drugs, analogues with a reduced net positive charge have also been developed, of which NAB739 (Figure 1) is among the best-studied examples.^28,33,34^ Originally developed by Vaara and co-workers, NAB739 was found to be less toxic toward a porcine proximal tubule cell line,^28^ while outperforming polymyxin B in a mouse infection model of pyelonephritis caused by *Escherichia coli*.^35^ Structural variation of the fatty acid moiety has also been investigated as an approach to tuning the activity/toxicity ratio of the polymyxins. Researchers at Pfizer reported a series of biaryl amide substituted polymyxins with very promising activity and toxicity data on human renal proximal tubular epithelial cells (PTECs). However, while the lead compound (5X, Figure 1) exhibited low toxicity in rats, it failed to do so in dogs where it was found to be no better than polymyxin B.^30^ Similarly, recent work by the groups of Blaskovich and Cooper revealed that polymyxin analogues containing fatty acids comprising aromatic biphenyls or biphenyl ethers maintain activity against relevant Gram-negative strains.^36^ These analogues were found to exhibit reduced nephrotoxicity as indicated by decreased levels of lactate dehydrogenase and γ-glutamyl transferase released by human primary kidney cells relative to those measured upon exposure to polymyxin B.^36^ In addition, Brown and co-workers recently disclosed SPR206 (Figure 1), a next-generation polymyxin bearing an N-terminal, β-branched aminobutyrate moiety featuring an aryl substituent.^29^ This compound was found to maintain potent antibacterial activity while showing lower cytotoxicity than polymyxin B in cell-based models as well as lower kidney exposure and toxicity in mouse models.^29^ In another approach, researchers at MicuRx recently disclosed a novel series of polymyxin analogues wherein the lipid tail is connected to the peptide via a hydrolytically labile ester linkage (Figure 1).^37^ The operating principle in these polymyxin analogues revolves around the exploitation of blood plasma esterases for the hydrolysis of the ester-linked lipid, a metabolic process envisioned to convert the active drug into less toxic metabolites. The first PK/PD and efficacy data for these interesting new analogues were reported in 2021 and a phase I trial is now underway with the lead candidate MRX-8 (specific structure not disclosed).^38^ Another approach aimed at developing polymyxins that are converted to a less toxic metabolite *in vivo* was recently reported by Rabanal and co-workers at the University of Barcelona wherein analogues containing a disulfide motif within the heptapeptide cycle were prepared and evaluated (Figure 1).^39^ In doing so, Dab4 and Thr10 were replaced by cysteine residues and the heptapeptide ring closure was subsequently achieved by disulfide formation. The most promising compound was equipped with a C10 lipid tail and norleucine at P6, which interestingly also led to activity against Gram-positive species including *Staphylococcus aureus*. While this compound was found to exhibit low acute toxicity, its nephrotoxicity appeared to be similar to that of polymyxin B, an issue that could be partially resolved by restoring the original hydroxyl-group-containing side chain at P10.^40^

As noted above, the nephrotoxicity of the polymyxins is attributed to their propensity to accumulate in kidney cells, an effect that appears to be associated with the presence of the N-terminal lipid tail given the comparatively reduced nephrotoxicity of PMBN. In considering alternative approaches to addressing this issue, we were intrigued by the potential to exploit the large difference in the reducing environment found inside proximal tubular cells relative to that of the bloodstream. The intracellular glutathione concentration of renal proximal tubule cells is approximately 5 mM^41^ while in healthy adults blood plasma concentrations of glutathione are nearly 1000-fold lower.^42^ To this end, we here report the design, synthesis, and evaluation of a series of polymyxin analogues wherein the lipid tail is connected to the peptide via a reductively labile disulfide linkage. The working hypothesis behind this strategy being that, upon entry to kidney cells, the disulfide-linked lipids are cleaved (owing to the high local concentration of glutathione) to generate polymyxin degradation products similar to the less toxic PMBN. A series of analogues were prepared via a convenient semisynthetic route allowing for the incorporation of a range of lipid groups. A number of the disulfide-linked polymyxins thus prepared were found to maintain the potent antibacterial activity of the clinically used antibiotic *in vitro*. Furthermore, the disulfide-linked polymyxins were found to be stable when incubated with glutathione at concentrations found in the bloodstream but were rapidly cleaved in the presence of glutathione at concentrations mimicking those found in renal cells. Notably, cell-based assays identified a subset of compounds that retained potent antibacterial activity while displaying lower toxicity than polymyxin B toward PTECs, the kidney cell type typically associated with the accumulation of polymyxins.^22,24^

## Results and Discussion

**Scheme 1:**
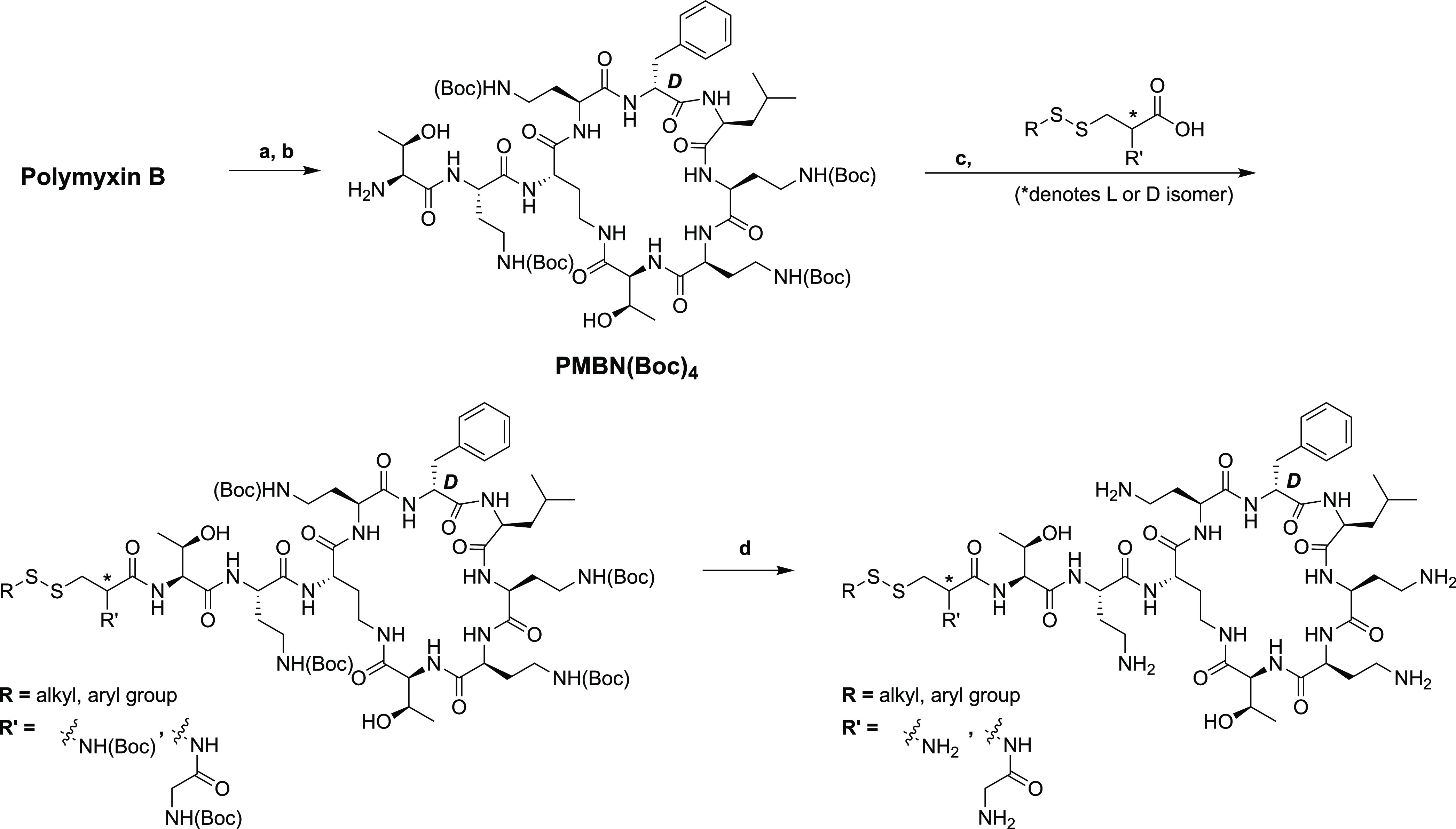
General Strategy for Semisynthesis of Disulfide-Containing Polymyxin Analogues Reagents and conditions: (a) ficin, dithiothreitol (DTT), water, 37 °C, o/n; (b) Boc-ON, Et_3_N, water/dioxane, room temperature (RT), 25 min; (c) (benzotriazol-1-yloxy)tris(dimethylamino)phosphonium hexafluorophosphate/*N*,*N*-di-isopropylethylamine (BOP/DIPEA), dichloromethane/dimethylformamide (DMF/DCM), RT, o/n; (d) trifluoroacetic acid/triisopropylsilane (TFA/TIPS)/H_2_O, RT, 1.5 h.

In designing our semisynthetic, disulfide-linked lipid polymyxin analogues, we elected to replace the N-terminal Dab residue with cysteine (general strategy illustrated in Scheme 1). This provided a convenient means for introducing the disulfide-linked lipid at the N-terminus. In addition, the α-amino group of Cys approximates the side chain amine of the Dab1 residue, which is important for antimicrobial activity.^36^ To this end, we first prepared a series of cysteine-based building blocks containing a disulfide-linked lipid for subsequent coupling to the polymyxin core. The polymyxin core peptide was in turn generated by an established chemoenzymatic process wherein the N-terminal Dab residue and lipid were enzymatically removed.^43^ To this end, polymyxin B was first treated with the commercially available plant protease ficin to obtain PMBN. Selective Boc protection of the four Dab side chain amines was then achieved by treatment with 2-(tert-butoxycarbonyloxyimino)-2-phenylacetonitrile (Boc-ON) to afford the PMBN(Boc)_4_ building block on gram scale (see Scheme 1). Subsequent coupling of the disulfide-linked lipidated cysteine building blocks was then performed using standard amide-bond-forming conditions after which global deprotection with acid yielded the desired polymyxin analogues (Scheme 1).

**Scheme 2:**
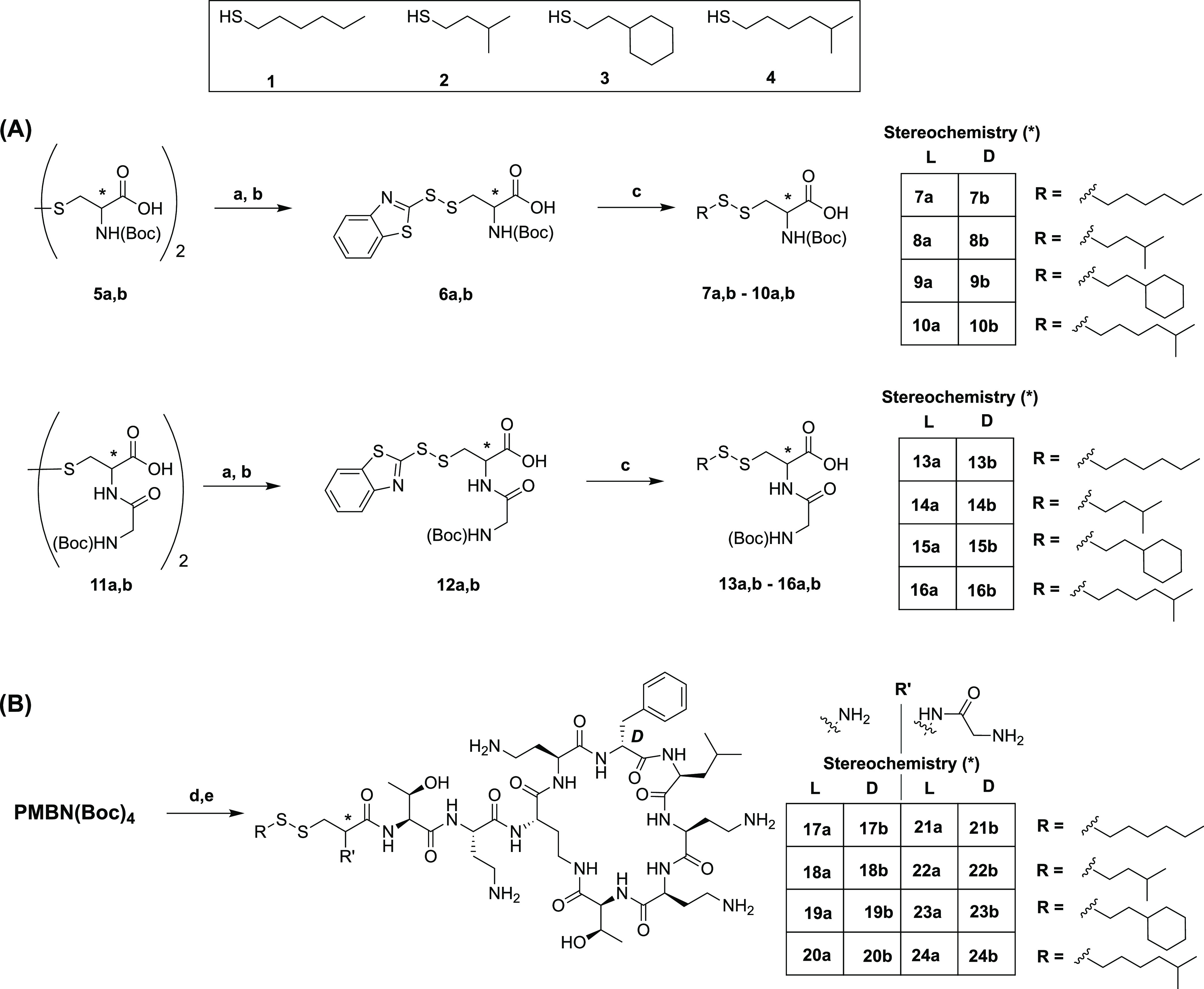
Synthesis of Disulfide-Containing Polymyxin Analogues with Aliphatic Tails, Based on Cysteine-Containing Disulfides (A) Synthesis of cysteine-containing disulfides; (B) conjugation to protected polymyxin B nonapeptide. Reagents and conditions: (a) PPh_3_, H_2_O, THF, 50 °C, o/n; (b) 2,2′-dithiobis(benzothiazole), CHCl_3_, RT, 3 h; (c) corresponding thiol (**1**–**4**), CHCl_3_, RT, o/n_;_ (d) corresponding disulfide building block (**7a,b**–**10a,b** and **13a,b**–**16a,b**), BOP, DIPEA, RT, o/n; (e) TFA, TIPS, H_2_O, RT, 1.5 h.

In the first generation of analogues explored, we specifically aimed to introduce disulfide-linked lipids that closely mimicked the all-carbon aliphatic lipid tails found naturally among the polymyxins. When produced by fermentation, polymyxins are obtained as mixtures of structurally similar isomers with slight differences in their fatty acid tails.^44^ In general, the antimicrobial potencies of these naturally occurring variants are similar.^45−47^ In our design, we therefore prepared disulfide-linked lipid analogues consisting of linear and branched aliphatic tails (see Scheme 2A). In addition, to further explore the structure–activity-toxicity relationships of our disulfide-linked lipid analogues, both l and d stereochemistries at the cysteine α-carbon were explored (compound numbering as **X“a”** and **X“b”**, respectively). Preparation of the Cys-derived building blocks first required access to aliphatic thiols **1**–**4** that were obtained commercially as for **1** and **2**, or synthesized as for **3** and **4** (see Scheme 2A and Supporting Information Scheme S1). In the case of the starting cysteine building blocks used, the corresponding l- and d-cystine species offered the advantage of not requiring protection of the thiol side chain. To this end, *N*,*N*-di-Boc-protected **5a,b** was reduced by treatment with triphenylphosphine and the free thiol species directly reacted with 2,2′-dithiobis(benzothiazole) to form activated disulfides **6a,b**.^48^ These intermediates provided a convenient means for the introduction of the disulfide-linked lipids wherein reaction with the aliphatic thiol provided the desired products (**7a,b**–**10a,b**) in good yields. Notably, when an excess of the aliphatic thiol (typically 2–3 equiv) was used, the reaction was found to proceed to completion at room temperature, without formation of the undesired cystine disulfide, after which the excess aliphatic thiol and mercaptobenzothiazole byproduct were easily removed by column chromatography. A complementary set of disulfide-linked lipidated Cys building blocks were also prepared using the same lipid moieties but with a glycine unit appended to the cysteine amino group (see building blocks **13a,b**–**16a,b**Scheme 2A). This was done to probe the importance of the location of the free amine at the N-terminus and to more closely mimic the placement of the amino group of the Dab side chain as found in natural polymyxins. Starting from either l- or d-cystine, coupling with Boc-Gly-Osu gave carboxylic acids **11a,b**. Subsequent reduction and reaction with 2,2′-dithiobis(benzothiazole) yielded the asymmetric disulfide intermediates **12a,b**, which upon treatment with thiols **1**–**4**, yielded **13a,b**–**16a,b**. Building blocks **7a,b**–**10a,b** and **13a,b**–**16a,b** were then coupled to PMBN(Boc)_4_ in an overnight reaction using a 2-fold excess of the cysteine-derived building block with BOP/DIPEA activation leading to complete consumption of the PMBN(Boc)_4_ (based on LCMS assessment). The crude intermediates were then concentrated and directly deprotected by treatment with TFA/TIPS/H_2_O after which reversed-phase-high performance liquid chromatography (RP-HPLC) purification (see the General Procedures section) yielded polymyxin analogues **17a,b**–**20a,b** and **21a,b**–**24a,b** (Scheme 2B).

The antibacterial activities of polymyxin analogues **17a,b**–**20a,b** and **21a,b**–**24a,b** were next evaluated against selected ATCC strains of *E. coli*, *K. pneumoniae*, *A. baumannii*, and *P. aeruginosa* (Table 1). Polymyxin B and PMBN were also included as reference compounds. In line with expectation, PMBN was found to exhibit no bactericidal activity on its own (up to 32 μg/mL), while polymyxin B effectively prevented bacterial growth, with low minimum inhibitory concentration (MIC) values. Gratifyingly, several of the disulfide-linked lipid polymyxin variants were also found to display good antimicrobial activity, indicating that disulfide-linked lipids are tolerated and that the polymyxin Dab residue at P1 can be replaced by cysteine-based building blocks. Notably, the l-cysteine-derived compounds lacking the additional N-terminal Gly motif (**17a**, **18a**, **19a**, **20a**) exhibited reduced activity, especially against *A. baumannii*. The observation that **20a** is the most active of these four analogues indicates that antibacterial activity can be enhanced by the introduction of a more hydrophobic alkyl tail, a finding in line with previous reports.^29^ Interestingly, coupling of a Gly unit to the amine of the N-terminal l-Cys residue was found to improve the activity for all compounds (**21a**, **22a**, **23a**, **24a**) against the tested strains. Also, in comparison to the l-Cys-containing compounds, the analogues in which the disulfide-linked lipid was appended to a d-Cys residue were generally more active. This is particularly clear for **17b**–**19b** with **18b** showing MIC values on par with polymyxin B. Interestingly, in this series, the analogue with the larger branched lipid (**20b**) was found to exhibit somewhat lower activity. The effect of coupling a Gly unit to the amine of the d-Cys residue was also examined (as in compounds **21b**, **22b**, **23b**, and **24b**); however, this was found to provide only a marginal gain in potency. Collectively, these results suggest that for the polymyxin analogues here explored, the stereochemistry of the d-Cys residue plays a key role in governing antibacterial activity. Appending a Gly motif to the terminal Cys residue only led to increased antimicrobial activity for the inferior analogues bearing an N-terminal l-Cys.

**Table 1:**
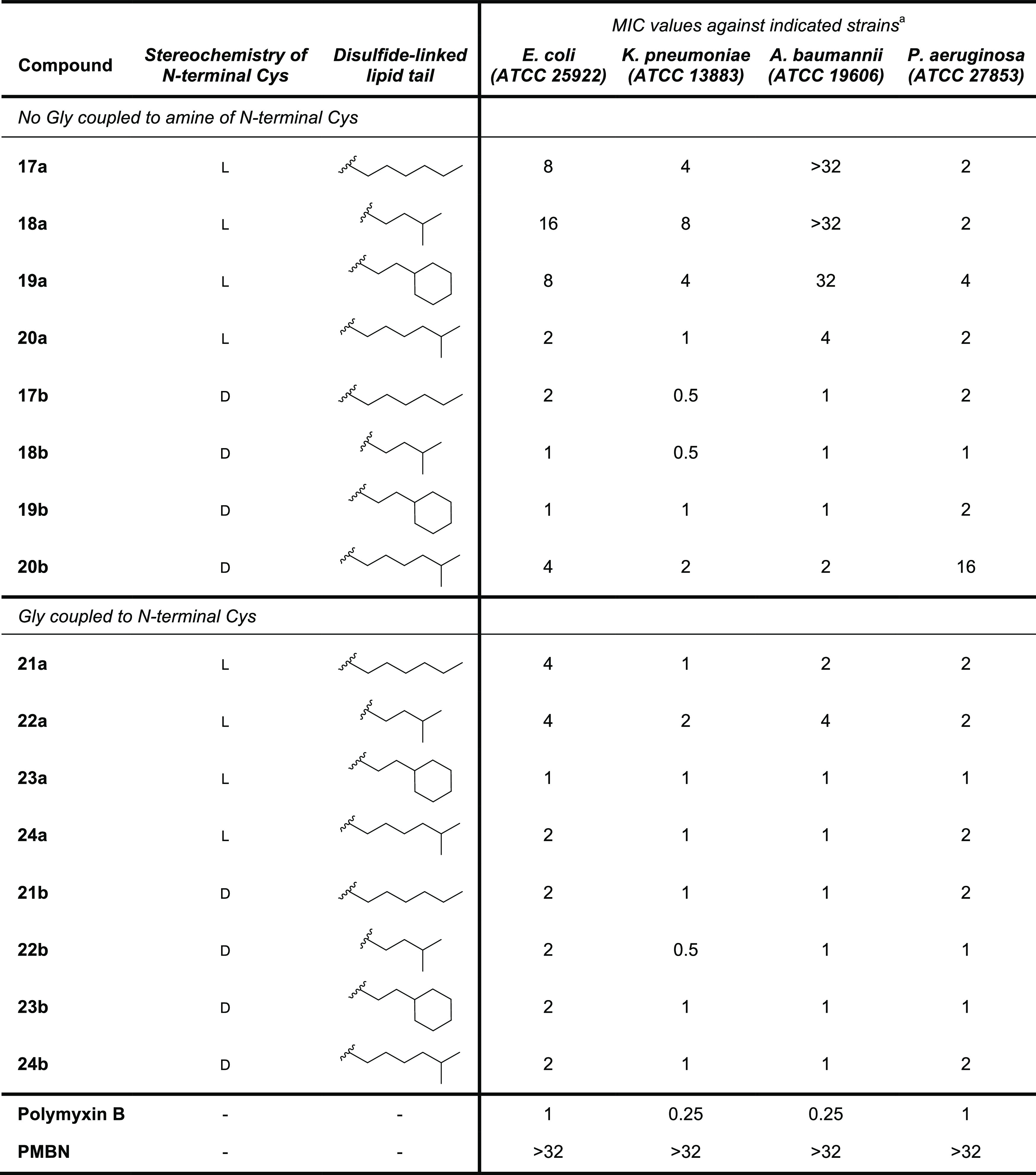
MIC Values for Disulfide-Linked Lipid Polymyxin Analogues **17a,b**–**20a,b** and **21a,b**–**24a,b** against Selected Gram-Negative Bacteria

a MIC values are derived from triplicate experiments and are expressed as μg/mL.

**Scheme 3:**
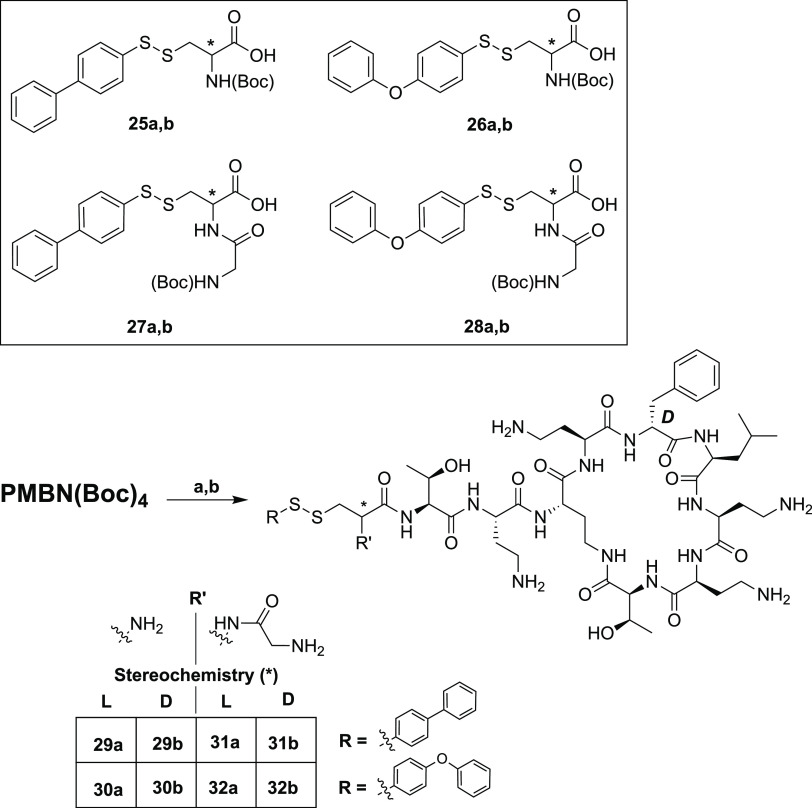
Synthesis of Disulfide-Containing Polymyxin Analogues with Aromatic Tails, Based on Cysteine-Containing Disulfides Reagents and conditions: (a) corresponding cysteine disulfide (**25a,b**–**28a,b**), BOP, DIPEA, RT, o/n; (b) TFA, TIPS, H_2_O, RT, 1.5 h.

In addition to the aliphatic acyl chains found in the natural polymyxins, aromatic moieties and especially biphenyl systems have been explored in several studies.^29,36,49^ To further expand our library of analogues, we therefore chose to also prepare a series of polymyxins wherein aromatic moieties were linked to the N-terminus via a disulfide linkage. In doing so, we used [1,1′-biphenyl]-4-thiol and 4-phenoxybenzene thiol to form asymmetric disulfides with cysteine and then conjugated those to PMBN(Boc)_4_ (Scheme 3). The necessary [1,1′-biphenyl]-4-thiol and 4-phenoxybenzene thiol building blocks were synthesized from 4-iodo-1,1′-biphenyl and 1-iodo-4-phenoxybenzene, respectively, via a copper-catalyzed reaction with elemental sulfur followed by treatment with NaBH_4_ (Supporting Information Scheme S2A).^50^ While this approach provided access to the desired thiol, significant quantities of the corresponding symmetric disulfide were found to form during purification with column chromatography and were difficult to separate. Fortunately, we found that treatment of the column-purified thiol/disulfide mixtures with zinc dust under acidic aqueous conditions provided the thiol in a purity suitable for use in the following step. The direct treatment of Boc-l-Cys with the aromatic thiols in turn led to the formation of building blocks **25a** and **26a** while coupling with Boc-d-Cys resulted in **25b** and **26b** (Supporting Information Scheme S2B). For the preparation of the Cys-based building blocks with an additional Gly motif **27a,b** and **28a,b**, we opted to react the aromatic thiols with the previously used mercaptobenzothiazole-containing intermediates **12a** and **12b** (Supporting Information Scheme S2C). The subsequent conjugation of **25a,b**–**28a,b** to PMBN(Boc)_4_ and subsequent deprotection provided access to the eight polymyxin analogues **29a,b**–**32a,b** bearing disulfide-tethered aromatic groups at the N-terminus (Scheme 3).

The antibacterial activities of **29a,b**–**32a,b** were assessed against the same panel of Gram-negative bacteria (Table 2) and found to be generally lower than the aliphatic analogues (Table 1). This reduced activity was somewhat surprising given the reported potency of amide-linked biphenyl and biphenyl ether polymyxin conjugates.^36^ In general, there was little difference in the MIC values observed for analogues bearing the biphenyl and biphenyl ether tails. Also, unlike the analogues bearing the aliphatic lipid tails, the activities observed for the aromatic series were not found to show a strong dependence on the stereochemistry of the Cys residue to which the lipid is attached. Furthermore, while in the aliphatic series the activity was improved by the addition of a Gly residue at the N-terminal Cys, the same trend is not seen for the aromatic analogues.

**Table 2:**
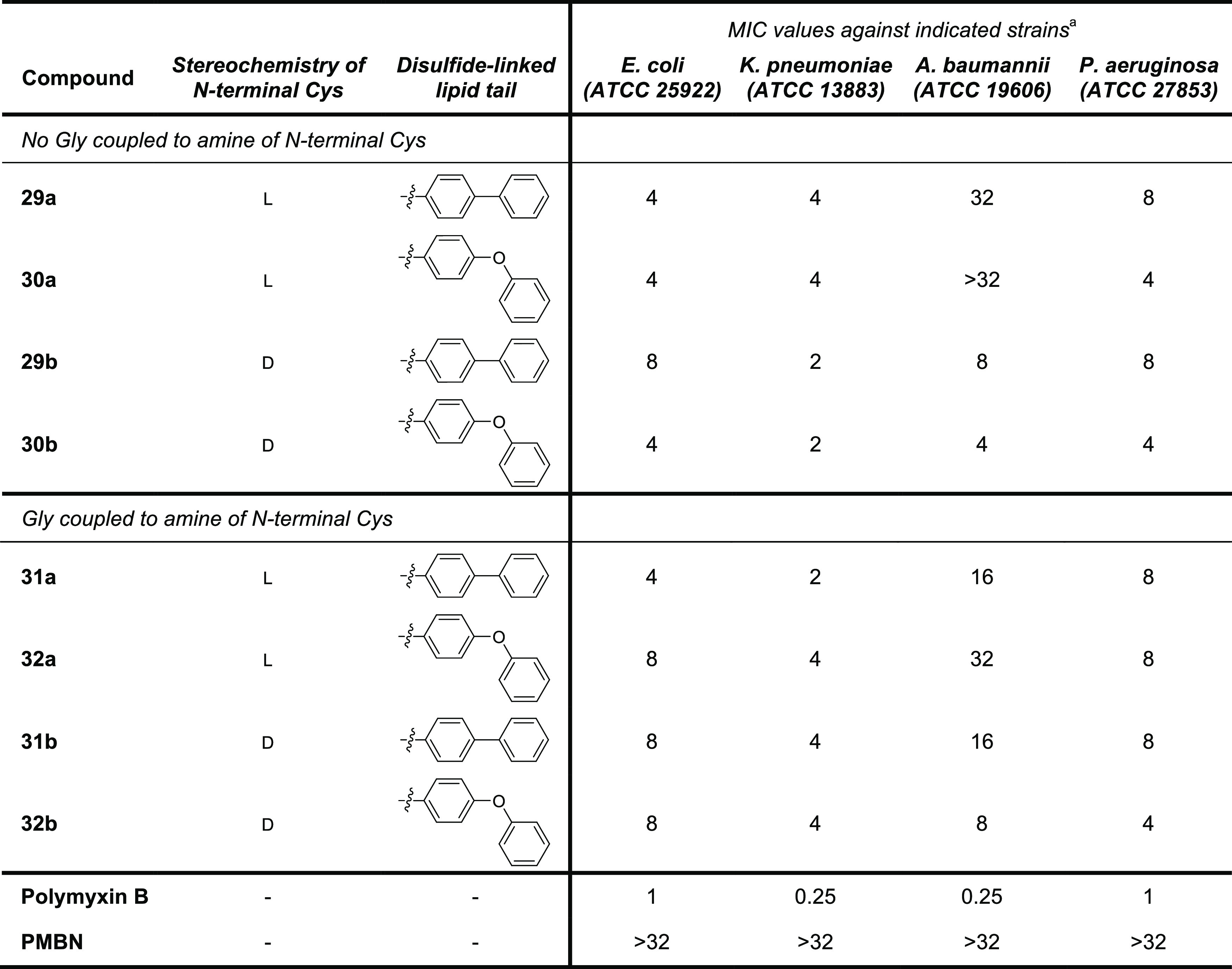
MIC Values for Disulfide-Linked Lipid Polymyxin Analogues **29a,b**–**30a,b** and **31a,b**–**32a,b** against Selected Gram-Negative Bacteria

a MIC values are derived from triplicate experiments and are expressed as μg/mL.

**Scheme 4:**
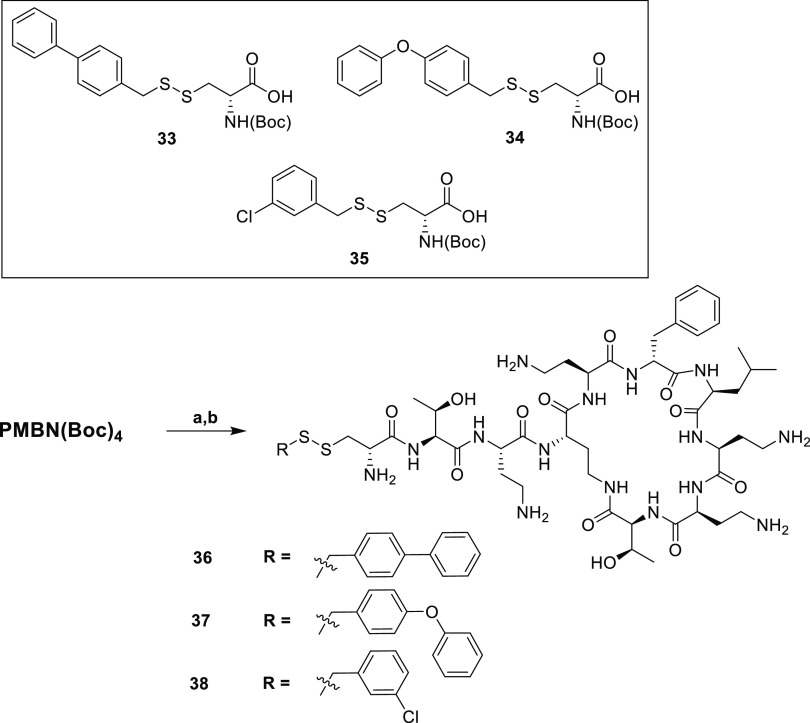
Synthesis of Disulfide-Containing Polymyxin Analogues with Benzylic Tails Based on d-Cysteine-Containing Disulfides Reagents and conditions: (a) corresponding cysteine disulfide (**33**–**35**), BOP, DIPEA, RT, o/n; (b) TFA, TIPS, H_2_O, RT, 1.5 h.

The moderate antibacterial activity of analogues **29a,b**–**32a,b** prompted to us to further investigate the linkage between the aromatic group and the disulfide moiety. Specifically, we were interested in knowing whether a benzyl motif, expected to impart greater flexibility, could be of benefit. To this end, we synthesized three additional aromatic analogues wherein a methylene group was introduced between the disulfide and the aromatic unit. In addition to analogues **36** and **37**, bearing the biphenyl and biphenyl ether groups, respectively, we also prepared compound **38** featuring a 3-chlorobenzyl motif inspired by the N-terminal moiety featured in polymyxin analogue SPR206 recently disclosed by Spero Therapeutics (Figure 1).^29^ Also, we opted to prepare **36**–**38** as only the d-Cys variants given that among the analogues described above those with d-Cys at P1 consistently showed superior activity to the corresponding l-Cys species. The required building blocks **33**–**35** were prepared from the corresponding thiols and intermediate **6b** (Supporting Information Scheme S3). Conjugation of **33**–**35** to PMBN(Boc)_4_, followed by global deprotection and HPLC purification, afforded polymyxin analogues **36**–**38** (Scheme 4). Assessment of the antibacterial activities for **36**–**38** (Table 3) revealed that analogues **36** and **37** show improved activities relative to their nonbenyzlic counterparts **29b** and **30b**. Analogue **38** containing the 3-chlorobenzyl motif also shows good antimicrobial potency, narrowly outperforming **36** and **37** against *E. coli* and *K. pneumoniae*. These results indicate that a well-positioned chlorine atom can recapitulate the contribution made by the second benzene ring, an observation in line with previous reports describing structure–activity relationship studies with different polymyxin analogues.^29,51^

**Table 3:**
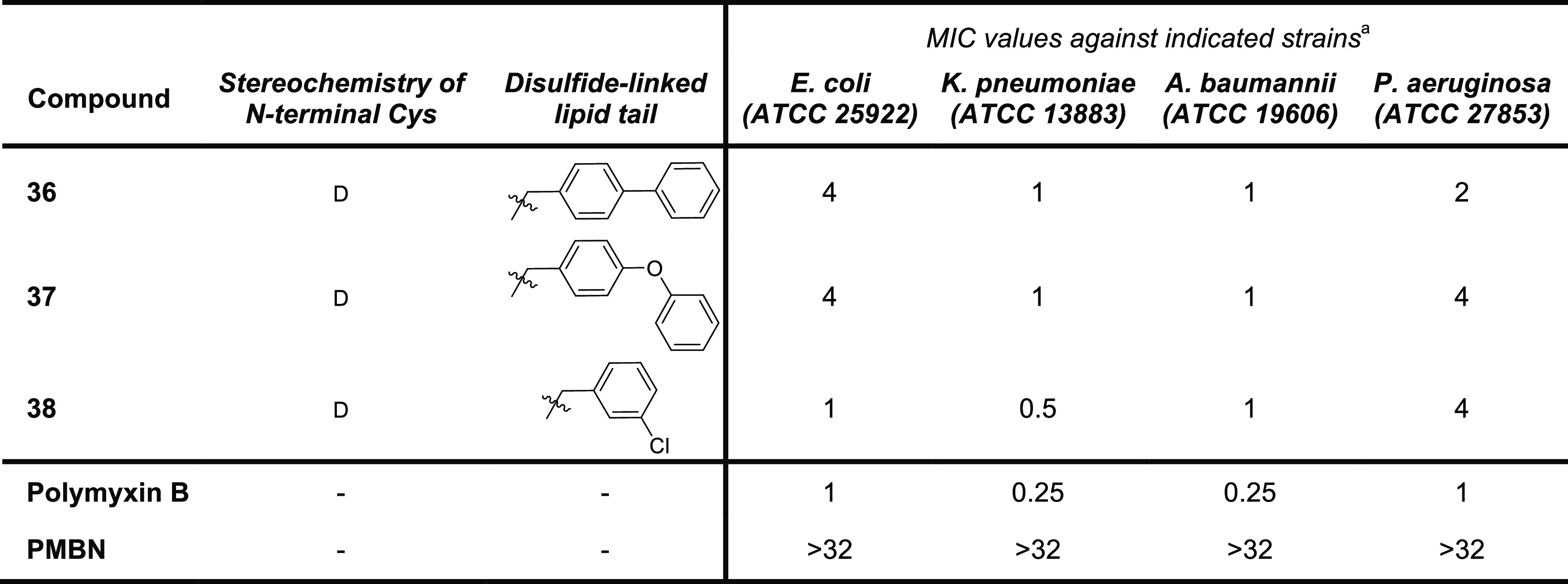
MIC Values for Disulfide-Linked Lipid Polymyxin Analogues **36**–**38** against Selected Gram-Negative Bacteria

a MIC values are derived from triplicate experiments and are expressed as μg/mL.

We next tested the hypothesis that the disulfide linkage incorporated in these polymyxin analogues could be selectively cleaved under different reducing conditions. As noted above, there is an approximate 1000-fold difference in the glutathione concentration found inside proximal tubular cells and in blood.^41,42^ To mimic these conditions, we assessed the stability of two of the more active analogues, **18b** and **23a**, in the presence of high (5 mM) and low (5 μM) glutathione concentrations in phosphate-buffered saline (PBS). The selected compounds were incubated for 24 h at 37 °C, with regular sampling and RP-HPLC analysis to monitor the stability of the compounds (Supporting Information Figure S1). Under conditions mimicking the higher intracellular concentration of glutathione, both **18b** and **23a** were completely degraded within the first hour of exposure. In contrast, when exposed to the milder reducing conditions (meant to mimic blood plasma levels of glutathione) only 10% degradation was seen in the first hour, with 70–80% of both compounds intact after 24 h. These findings suggest that the disulfide-linked lipid strategy may indeed provide a means to tune the metabolic stability of polymyxin analogues.

### Evaluation of Cell-Based Toxicity

The cell-based toxicity of the disulfide-linked lipid polymyxin analogues was next evaluated against both erythrocytes and kidney cells. Gratifyingly, it was found that the new analogues induced little-to-no hemolysis (Supporting Information Figure S2). The nephrotoxicity associated with polymyxins is generally ascribed to their damaging effects on proximal tubular epithelial cells (PTECs) which exhibit excessive absorption of polycationic drugs.^22^ For this reason, *in vitro* toxicity screening using differentiated PTECs is seen as more predictive of cytotoxicity than testing on nondifferentiated cell lines.^29,30^ To this end, we employed differentiated conditionally immortalized PTECs (ciPTECs)^52,53^ to evaluate the toxicity of a selection of the most active polymyxin analogues prepared (Table 4). Residual mitochondrial activity of the ciPTECs after 24h of incubation with the polymyxin species was used as read-out.^52^ The selected polymyxin analogues were assessed at multiple concentrations covering a range of 0–100% viability, thereby generating robust TC_50_ (concentration required for half-maximal toxic effect) values. The nephrotoxicity of polymyxins and their derived nonapeptides has been studied for decades, using both *in vitro*(54) or *in vivo*(20,55) methods, and it is well established that PMBN is considerably less toxic than full-length polymyxin.^20,54,55^ As seen in Table 4, in our ciPTEC-based assays, the TC_50_ of PMBN was found to be more than 10 times higher than that of polymyxin B. This result matches well with those previously reported,^30^ indicating the suitability of our cellular model for evaluating the renal toxicity of the antibacterial compounds prepared as part of the current study.

**Table 4:**
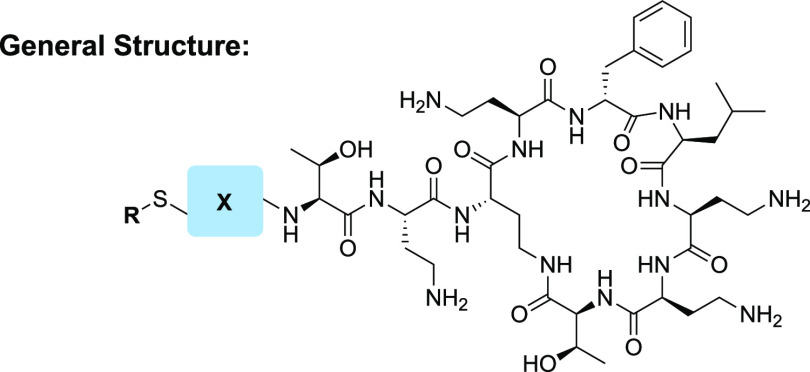

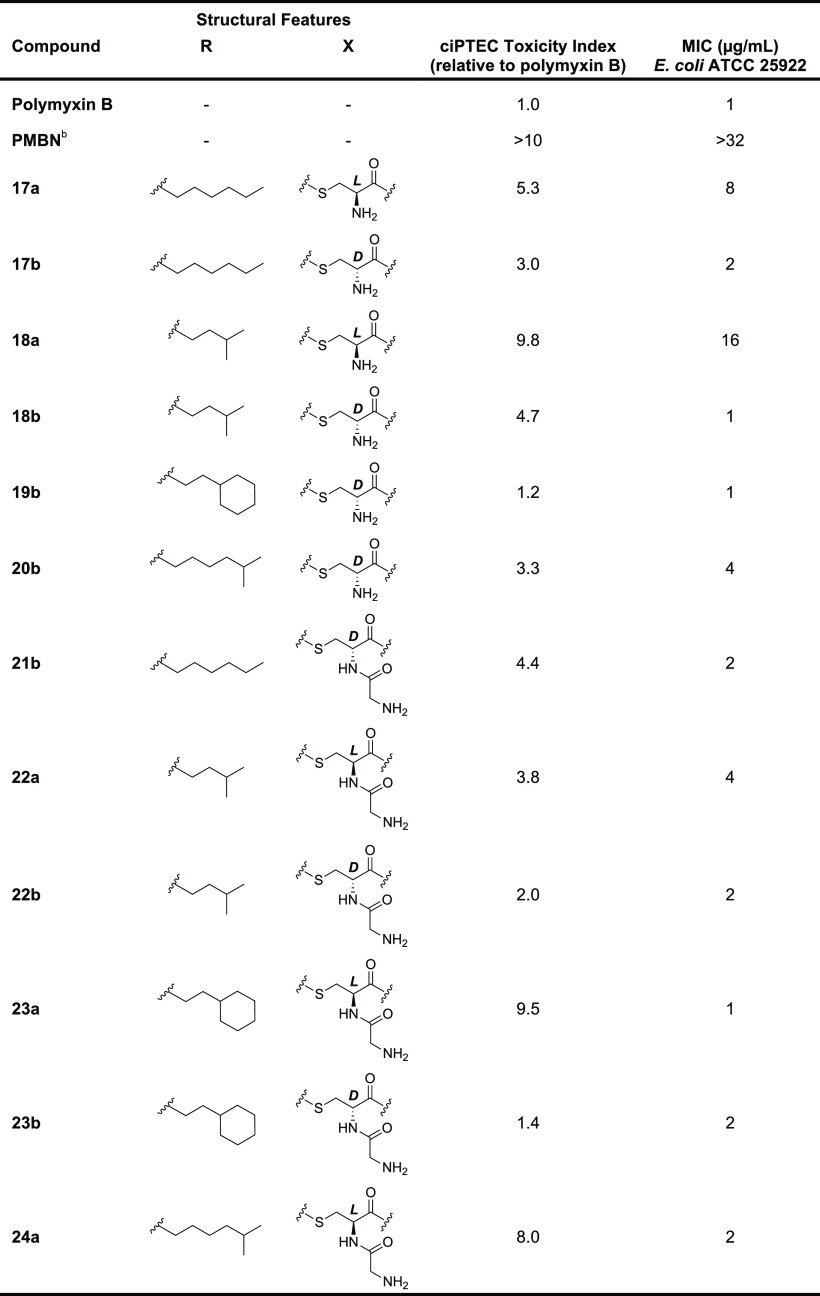

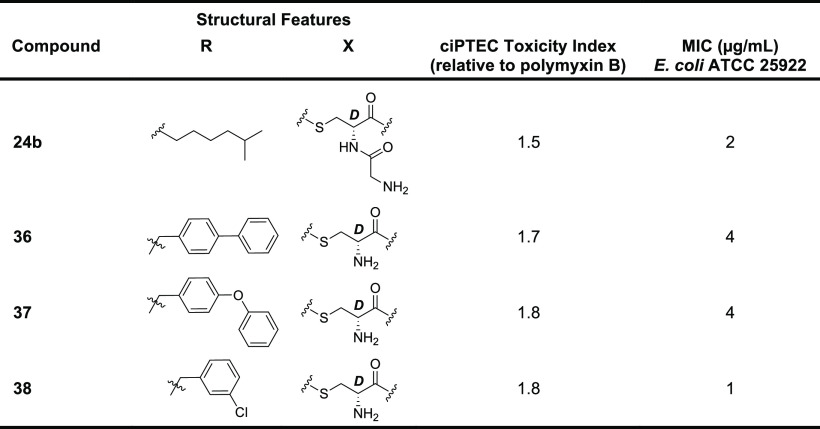
Cellular Toxicity of Disulfide Lipid-Linked Polymyxin Analogues as Determined on ciPTECs^a^

a Values are expressed relative to the toxicity of polymyxin B and represent the TC_50_ values determined after nonlinear regression. MIC values determined against *E. coli* ATCC 25922 (derived from Tables 1–3) included to provide comparison of antibacterial activity. Experiments were run in triplicate.

b PMBN: Polymyxin B nonapeptide.

Encouragingly, all of the analogues tested were found to be less toxic than polymyxin B (Table 4 and Supporting Information Table S1, Figure S3). There was a range of toxicities observed among the compounds with antibacterial activities similar to polymyxin B. Analogues **19b**, **22b**, **23b**, **24b**, and **38** showed a modest 1.2- to 2.0-fold reduction in cell toxicity relative to polymyxin B while **17b** and **21b**, were found to be 3.0 and 4.4 times less toxic, respectively. Of particular note, however, are analogues **18b**, **23a**, and **24a**, which were found to exhibit a 4.7-, 9.5-, and 8.0-fold reduction in cell toxicity, respectively, while maintaining potent antibacterial activity. While **18a** is among the least toxic analogues, its antibacterial activity is moderate with an MIC of 16 μg/mL. Interestingly, **18b** wherein the terminal Cys residue was stereochemically inverted to d-Cys resulted in a significant increase in antibacterial potency (MIC 1.0 μg/mL) while maintaining a reduced cell toxicity of some 4.7-fold lower than polymyxin B. Also of note, the addition of Gly to the terminal l-Cys reside in **18a** leads to **22a**, which shows some improvement in activity (MIC 4.0 μg/mL) along with a moderate increase in cell toxicity. Interestingly, the introduction of a slightly longer aliphatic tail as in **23a**, **24a** further enhances antibacterial activity while lowering toxicity. Given their promising combination of antibacterial activity and reduced cell toxicity, polymyxin analogues **18b**, **23a**, and **24a** were further tested on an expanded panel of Gram-negative strains including drug-resistant isolates against which all three compounds maintained potent activity (Supporting Information Table S2). These findings indicate that the balance between activity and toxicity can be fine-tuned by: (i) proper choice of stereochemistry at the disulfide-linked Cys residue; (ii) structural elaboration at the Cys amino group; and (iii) variation of the length of the lipid moiety.

### Contribution of Disulfide Linkage to Reduced Toxicity

**Scheme 5:**
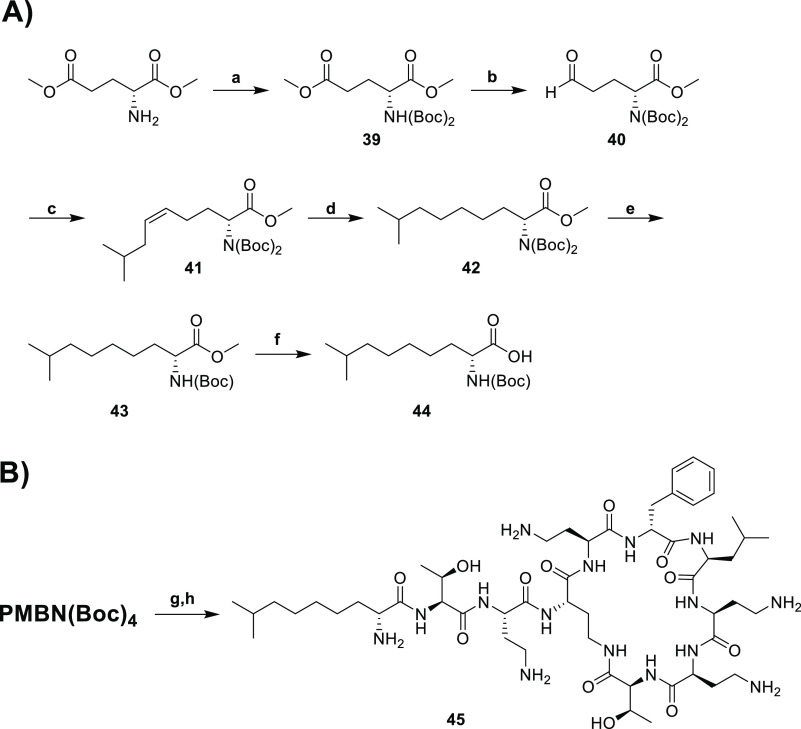
Synthesis of the Polymyxin Analogue **45** Lacking a Reductively Labile Disulfide Bond (A) Synthesis of Boc-protected amino acid building block **44**. (B) Conjugation to PMBN(Boc)_4_ and subsequent deprotection and purification. Reagents and conditions: (a) (i) Et_3_N, (Boc)_2_O, DCM, RT, o/n; (ii) DMAP, (Boc)_2_O, acetonitrile, RT, o/n; (b) DIBAL-H, Et_2_O, −78 °C, 15 min; (c) (3-methylbutyl)triphenylphosphonium bromide, potassium bis(trimethylsilyl)amide, toluene, −78 °C, 2 h; (d) Pd/C, triethylsilane, EtOH, RT, 40 min; (e) (i) HCl, THF/dioxane, RT, 4h; (ii) Et_3_N, (Boc)_2_O, MeOH, RT, o/n; (f) NaOH, dioxane/H_2_O, RT, o/n; (g) **44**, BOP, DIPEA, RT, o/n; (h) TFA, TIPS, H_2_O, RT, 1.5 h.

To establish the extent to which the disulfide motif in these polymyxin analogues contributes to their reduced toxicity, we also synthesized and evaluated a variant of **18b** wherein the disulfide was replaced with the corresponding C–C linkage. To do so, Boc-protected (*R*)-2-amino-8-methylnonanoic acid building block **44** was required. This was synthesized starting from the bismethyl ester of glutamic acid as shown in Scheme 5.^56^ Double Boc protection resulted in intermediate **39**, which was in turn selectively reduced using DIBAL-H to give aldehyde **40**. A Wittig reaction with the commercially available isopentyltriphenylphosphonium bromide yielded δ,ε-unsaturated ester **41** that was subsequently reduced to give protected amino acid **42**. Removal of both Boc groups under acidic conditions followed by mono-Boc protection afforded intermediate **43**, after which ester hydrolysis provided access to building block **44**. Coupling of **44** to PMBN(Boc)_4_ followed by deprotection and purification by RP-HPLC was performed as for the other analogues to yield polymyxin variant **45** (Scheme 5B).

The antibacterial activity of all-carbon lipid-containing analogue **45** was tested against a number of strains and found to be on par with that of disulfide-linked analogue **18b** (Supporting Information Table S3). With regard to its effect on kidney cells, analogue **45** was found to be 2.4 times more toxic toward ciPTECs than **18b** (TC_50_ of 82 μm for **45** vs 192 μM for **18b**). Based on these findings, it appears that the reductively labile disulfide linkage in the lipid tail makes a significant contribution to the reduced toxicity observed for analogue **18b**.

## Conclusions

While polymyxins have been part of the clinical arsenal of antibiotics for more than half a century, their systemic use has historically been minimized due to toxicity concerns. The emergence of extensively resistant Gram-negative pathogens has led to renewed interest in the use of these lipopeptide agents as last-resort therapies and has spurred interest in next-generation polymyxins with improved safety profiles. The nephrotoxicity ascribed to the polymyxins is attributed to their capacity to accumulate in kidney cells. Notably, the interior of such cells presents a strongly reducing environment by virtue of the much higher glutathione concentration in comparison with that found in the bloodstream. We here report efforts aimed at developing a new class of semisynthetic polymyxins bearing a reductively labile disulfide linkage connecting the peptide and lipid moieties. The working principle behind this approach stems from the knowledge that the removal of the lipid tail from the polymyxin core results in products with significantly reduced nephrotoxicity. In pursuing these next-generation polymyxins, an efficient semisynthetic approach was employed providing access to a number of new analogues with differing disulfide-linked lipids and N-terminal amino acids. The antibacterial activity of these analogues compared well with that of polymyxin B and cell-based studies identified a subset of compounds that also demonstrate reduced toxicity. Stability studies in the presence of varying glutathione concentrations, as well as comparison with a non-disulfide-linked comparator, indicate that the working principle behind these next-generation polymyxins may offer a means of tuning their toxicity by exploiting their metabolic instability.

## Experimental Section

### Reagents

All reagents employed were of American Chemical Society (ACS) grade or finer and were used without further purification unless otherwise stated. Commercially sourced Polymyxin B was obtained as a mixture of isomers (Combi-Blocks, San Diego), with polymyxin B1, B2, and B3 accounting for >90%.

### General Procedures

For compound characterization,^1^H NMR spectra were recorded at 400, 500, or 600 MHz, and chemical shifts are reported in parts per million downfield relative to CH_3_OH (δ 3.31), CHCl_3_ (δ 7.26) or DMSO (δ 2.50).^1^H NMR data are reported in the following order: multiplicity (s, singlet; d, doublet; t, triplet; q, quartet; and m, multiplet), coupling constant (J), and the number of protons. ^13^C NMR spectra were recorded at 101, 126, or 151 MHz, and chemical shifts are reported relative to CDCl_3_ (δ 77.16), methanol (δ 49.00), or DMSO (δ 39.52).

All polymyxin analogues prepared were purified via preparative HPLC using a BESTA-Technik system with a Dr. Maisch Reprosil Gold 120 C18 column (25 × 250 mm, 10 μm) and equipped with an ECOM Flash UV detector monitoring at 214 nm. The following solvent system, at a flow rate of 12 mL/min, was used: solvent A, 0.1% TFA in water/acetonitrile 95/5; solvent B, 0.1% TFA in water/acetonitrile 5/95. Gradient elution was as follows: 100:0 (A/B) for 3 min, 100:0 to 85:15 (A/B) over 2 min, 60:40 (A/B) over 45 min, 60:40 to 0:100 (A/B) over 3 min, 0:100 (A/B) for 3 min, then reversion back to 100:0 (A/B) over 1 min, 100:0 (A/B) for 3 min. Depending on the polarity of the compounds, gradients were chosen between 10 and 60% maximum concentration of solvent B.

The purity of the polymyxin analogues was confirmed to be ≥95% by analytical RP-HPLC using a Shimadzu Prominence-i LC-2030 system with a Dr. Maisch ReproSil Gold 120 C18 column (4.6 × 250 mm, 5 μm) at 30 °C and equipped with a UV detector monitoring at 214 nm. The following solvent system, at a flow rate of 1 mL/min, was used: solvent A, 0.1% TFA in water/acetonitrile, 95/5; solvent B, 0.1% TFA in water/acetonitrile, 5/95. Gradient elution was as follows: 100:0 (A/B) for 3 min, 100:0 to 0:100 (A/B) over 47 min, 0:100 (A/B) for 4 min, then reversion back to 100:0 (A/B) over 1 min, 100:0 (A/B) for 5 min. For compound characterization, HRMS analysis was performed on a Shimadzu Nexera X2 UHPLC system with a Waters Acquity HSS C18 column (2.1 × 100 mm, 1.8 μm) at 30 °C and equipped with a diode array detector. The following solvent system, at a flow rate of 0.5 mL/min, was used: solvent A, 0.1% formic acid in water; solvent B, 0.1% formic acid in acetonitrile. Gradient elution was as follows: 95:5 (A/B) for 1 min, 95:5 to 15:85 (A/B) over 6 min, 15:85 to 0:100 (A/B) over 1 min, 0:100 (A/B) for 3 min, then reversion back to 95:5 (A/B) for 3 min. This system was connected to a Shimadzu 9030 QTOF mass spectrometer (ESI ionization) calibrated internally with Agilent’s API-TOF reference mass solution kit (5.0 mM purine, 100.0 mM ammonium trifluoroacetate and 2.5 mM hexakis(1*H*,1*H*,3*H*-tetrafluoropropoxy)phosphazine) diluted to achieve a mass count of 10,000.

### Synthesis of PMBN

Polymyxin B sulfate salt (3.0 g, 2.1 mmol) was dissolved in water (90 mL). Dithiothreitol (79 mg, 0.51 mmol) and ficin (MP Biomedicals, 0.79 g, ∼0.02 mmol, declared activity 215 BAPA u/g) were dissolved in water (10 mL) and added to the dissolved polymyxin B. Enzymatic digestion was run at 37 °C under N_2_ atmosphere overnight. When required, additional dithiothreitol (20 mg, 0.13 mmol) and ficin (100 mg, 0.003 mmol) were added, and the digestion time was prolonged. After complete digestion, the mixture was heated to reflux for 20 min, cooled down, and filtered. The pH of the filtrate was adjusted to 2 (5 M HCl) and extracted with *n*-butanol (4 × 25 mL). The combined aqueous layers were neutralized (NaOH 6 M) and freeze-dried. The crude material (white powder) was used without further purification in the following synthetic step. For biological testing, pure PMBN was obtained by RP-HPLC (General Procedures section), employing a 10–35% B gradient.

### Synthesis of PMBN(Boc)_4_

PMBN (1.36 g (crude), 1.41 mmol) was dissolved in H_2_O (8.7 mL). Triethylamine (8.7 mL, 62.4 mmol) was added, and the mixture was stirred for 5 min. 2-(*tert*-Butoxycarbonyloxyimino)-2-phenylacetonitrile (1.55 g, 6.3 mmol) was dissolved in dioxane (8.8 mL) and added slowly to the PMBN solution. The mixture was stirred at RT for 30 min and quenched by methanolic NH_3_ (7 N, 6 mL). The mixture was concentrated, and the residue was dissolved in MeOH (130 mL). After filtration, the filtrate was concentrated and purified by flash chromatography,^43^ using a solvent system of 13% MeOH/1% Et_3_N/DCM. Yield: 1.2 g, 0.9 mmol, 64%.

### General Method for the Synthesis of Analogues from PMBN(Boc)_4_

PMBN(Boc)_4_ (0.1 g, 0.07 mmol) was dissolved in DCM (2 mL). (Benzotriazol-1-yloxy)tris(dimethylamino)phosphonium hexafluorophosphate (BOP, 65 mg, 0.15 mmol) and Boc-protected cysteine-derived disulfide (0.15 mmol) were dissolved in DCM (2 mL) and added to the PMBN(Boc)_4_ solution. DIPEA (51 μL, 0.29 mmol) was added, and the reaction was run at RT overnight. After reaction completion, the reaction mixture was concentrated and the residue was treated with TFA/TIPS/H_2_O (95:2.5:2.5, 5 mL) for 90 min. The solution was transferred to cold (−20 °C) methyl *t*-butyl ether (MTBE, 40 mL), and the mixture was spun down (4500 rpm, 5 min). The obtained pellet was suspended in MTBE and spun down (4500 rpm, 5 min). The resulting pellet was freeze-dried from H_2_O/*t*-butanol to yield an off-white powder that was purified by RP-HPLC.

### MIC Assays

Minimum inhibitory concentrations (MICs) on relevant Gram-negative bacteria were determined by broth microdilution, in accordance with CLSI guidelines. Indicated bacteria were taken from glycerol stocks and incubated overnight on blood agar at 37 °C. Well-isolated colonies were taken, suspended in TSB (5 mL), and grown to an OD_600_ of 0.5. Compounds to be tested were dissolved in DMSO (6.4 mg/mL), diluted in cation-adjusted Mueller–Hinton Broth (CAMHB) (12.5 and 20 μg/mL Mg^2+^ and Ca^2+^, respectively), and diluted serially on polypropylene microtiter plates. To the diluted compounds (50 μL per well) was added 50 μL of relevant bacterial suspension, to yield a final concentration of 10^6^ CFU/mL. Plates were covered by adhesive gas-permeable membranes and incubated at 37 °C. MIC was read out as the lowest concentration that inhibited visual bacterial growth. Shown values are consistent results from at least duplicate experiments.

### Glutathione Stability Assay

Glutathione stock concentrations of 5.5 mM and 5.5 μM were prepared in PBS; 1.35 mL was taken and mixed with the tested compound (1 mg/mL, 0.15 mL). The sample vial was flushed with N_2_ and placed at RT in the autosampler of the HPLC equipment. Samples were drawn at *t* = 0, 1, 2, 3, 4, 8, 10, and 24 h and analyzed by UV monitoring at 214 nm. An external calibration curve was constructed with the samples diluted in PBS. Calibration samples were run in duplicates.

### Hemolysis and Cytotoxicity Assays

#### Hemolysis Assay

Red blood cells from defibrinated sheep blood (Thermo Fisher Scientific) were centrifuged (400*g* for 15 min at 4 °C) and washed with PBS containing 0.002% Tween20 four times. The red blood cells were normalized to obtain an absorbance value between 2.5 and 3.0 at 415 nm to stay in the linear range of the assay with maximum sensitivity. A serial dilution of the compounds (128–4 μg/mL, 75 μL) was prepared in a 96-well plate. Each plate contained a positive control (0.1% Triton-X final concentration, 75 μL) and a negative control (buffer, 75 μL). The normalized blood cells (75 μL) were added, and the plates were incubated at 37 °C for 1 h while shaking at 500 rpm. After incubation, the plates were centrifuged (800*g* for 5 min at RT) and 25 μL of the supernatant was transferred to a second plate, containing 100 μL of PBS in each well. Absorbance was read-out at 415 nm. Values were corrected for background (negative control) and transformed to a percentage relative to the positive control. Results shown (Supporting Information Figure S2) are based on triplicate data.

#### ciPTEC Cell Culture

ciPTECs (OAT-1) cells,^52,53^ grown to 90–100% confluence, were washed by Hanks’ balanced salt solution (HBSS; Gibco, Life Technologies, Paisley, U.K.) and detached by accutase (4 mL) for 5 min at 37 °C. The cells were then suspended in fresh medium (DMEM/F-12, supplemented with 10% fetal calf serum, insulin (5 μg/mL), transferrin (5 μg/mL), selenium (5 μg/mL) hydrocortisone (35 ng/mL), Epidermal Growth Factor (10 ng/mL) and tri-iodothyronine (40 pg/mL)). Cell density was adjusted to 2.0 × 10^5^ cells/mL, of which 100 μL was added to each well of a 96-well plate. The cells were incubated for 24 h at 33 °C, followed by 6 days at 37 °C, in a humidified atmosphere containing 5% (v/v) CO_2_. The medium was refreshed every second or third day.

#### ciPTEC Toxicity Assay

Compounds were dissolved and serially diluted in serum-free medium. Differentiated cells were washed once with HBSS and serially diluted compounds (80 μL/well) were transferred to the ciPTEC-containing plate. Compounds were incubated for 24 h at 37 °C; the cells were washed by HBSS, followed by incubation with 10% PrestoBlue cell viability reagent (Thermo Scientific, Vienna, Austria) in HBSS at 37 °C in the dark. Fluorescence was recorded (excitation: 530 nm, emission: 590 nm). Raw data are corrected for PrestoBlue background fluorescence and reported relative to the no-treatment control (cells with medium only). Viability data were fitted with GraphPad Prism software (version 5.03; GraphPad Software, La Jolla, CA), by nonlinear regression with 0 as bottom constraint. Presented data are based on triplicates.

## Supporting Information Available

Analytical data including HRMS values, analytical HPLC traces, and supplemental tables and figures (PDF)
Molecular formula strings (CSV)

## Abbreviations Used

ACN: acetonitrile
ATCC: American Tissue Culture Collection
BAPA: *N*_α_-benzoyl-l-arginine-4-nitroanilide
BOP: benzotriazol-1-yloxy)tris(dimethylamino)phosphonium hexafluorophosphate
CAMHB: Cation-Adjusted Mueller–Hinton Broth
CFU: colony-forming units
CLSI: Clinical and Laboratory Standards Institute
Dab: 4-diaminobutyric acid
DIPEA: *N*,*N*-di-isopropylethylamine
DMEM: Dulbecco’s modified Eagle’s medium
HBSS: Hancks’ balanced salt solution
MIC: minimal inhibitory concentration
o/n: overnight
OAT-1: organic anion transporter-1
OM: outer membrane
PMBN: polymyxin B nonapeptide
ciPTEC: conditionally immortalized proximal tubule epithelial cell
RP-HPLC: reversed-phase high-performance liquid chromatography
TC_50_: half-maximum toxicity concentration
