# Supplementary material for: Synthesis and Evaluation of Polymyxins Bearing Reductively Labile Disulfide-Linked Lipids
Source: J Med Chem. 2022 Nov 18;65(23):15878–92. doi: 10.1021/acs.jmedchem.2c01528 (PMC9743094; doi:10.1021/acs.jmedchem.2c01528)
Supplement: Supplementary file 1 — jm2c01528_si_001.pdf [file jm2c01528_si_001.pdf]

Supporting information for:

Synthesis and Evaluation of Polymyxins Bearing Reductively Labile Disulfide-Linked Lipids

Cornelis J. Slingerland,[1] Charlotte M.J. Wesseling,[1] Koen G.C. Westphal,[2] Rosalinde Masereeuw,[2] Paolo Innocenti[1], Nathaniel I. Martin[1,*]

[1]Biological Chemistry Group, Institute of Biology Leiden, Leiden University, Sylviusweg 72, 2333 BE Leiden, The Netherlands.

[2]Division of Pharmacology, Utrecht Institute for Pharmaceutical Sciences, Utrecht University, 3584 CG, Utrecht, The Netherlands

*All correspondence should be directed to n.i.martin@biology.leidenuniv.nl

Table of contents

S2-S3	Reagents and General Procedures
S4-S29	Synthesis schemes, methods and analytical data on thiols and disulfide containing small molecules
S30-S32	Synthesis methods and analytical data on control compound **44**
S33-S47	HRMS data and HPLC traces of final peptide compounds
S48	Stabililty assessment in presence of glutathione
S48	Hemolysis assessment
S49	Toxicity assessment on PTECs
S50	Bacterial strains used for MIC studies
S50	Extended MIC assessment
S51	Activity and toxicity comparison of **18b**, **45** and **polymyxin B**.
S52	References

Reagents

All reagents employed were of American Chemical Society (ACS) grade or finer and were used without further purification unless otherwise stated. Commercially sourced Polymyxin B was obtained as a mixture of isomers (Combi-Blocks, San Diego, USA), with polymyxin B1, B2, and B3 accounting for >90%.

General Procedures

For compound characterization, ^{1}H NMR spectra were recorded at 400, 500 or 600 MHz, and chemical shifts are reported in parts per million downfield relative to CH_3OH (δ 3.31), $CHCl_3$ (δ 7.26) or DMSO (δ 2.50). ^{1}H NMR data are reported in the following order: multiplicity (s, singlet; d, doublet; t, triplet; q, quartet; and m, multiplet), coupling constant (J), and the number of protons. ^{13}C NMR spectra were recorded at 101, 126 or 151 MHz, and chemical shifts are reported relative to $CDCl_3$ (δ 77.16), methanol (δ 49.00), or DMSO (δ 39.52).

All polymyxin analogues prepared were purified via preparative HPLC using a BESTA-Technik system with a Dr. Maisch Reprosil Gold 120 C18 column (25 × 250 mm, 10 µm) and equipped with a ECOM Flash UV detector monitoring at 214 nm. The following solvent system, at a flow rate of 12 mL/min, was used: solvent A, 0.1 % TFA in water/acetonitrile 95/5; solvent B, 0.1 % TFA in water/acetonitrile 5/95. Gradient elution was as follows: 100:0 (A/B) for 3 min, 100:0 to 85:15 (A/B) over 2 min, 60:40 (A/B) over 45 min, 60:40 to 0:100 (A/B) over 3 min, 0:100 (A/B) for 3 min, then reversion back to 100:0 (A/B) over 1 min, 100:0 (A/B) for 3 min. Depending on the polarity of the compounds, gradients were chosen between 10% and 60% maximum concentration of solvent B.

Purity of the peptides was confirmed to be ≥ 95% by analytical RP-HPLC using a Shimadzu Prominence-i LC-2030 system with a Dr. Maisch ReproSil Gold 120 C18 column (4.6 × 250 mm, 5 µm) at 30 °C and equipped with a UV detector monitoring at 214 nm. The following solvent system, at a flow rate of 1 mL/min, was used: solvent A, 0.1 % TFA in water/acetonitrile, 95/5; solvent B, 0.1 % TFA in water/acetonitrile, 5/95. Gradient elution was

as follows: 100:0 (A/B) for 3 min, 100:0 to 0:100 (A/B) over 47 min, 0:100 (A/B) for 4 min, then reversion back to 100:0 (A/B) over 1 min, 100:0 (A/B) for 5 min.

For compound characterization HRMS analysis was performed on a Shimadzu Nexera X2 UHPLC system with a Waters Acquity HSS C18 column (2.1 × 100 mm, 1.8 µm) at 30 °C and equipped with a diode array detector. The following solvent system, at a flow rate of 0.5 mL/min, was used: solvent A, 0.1 % formic acid in water; solvent B, 0.1 % formic acid in acetonitrile. Gradient elution was as follows: 95:5 (A/B) for 1 min, 95:5 to 15:85 (A/B) over 6 min, 15:85 to 0:100 (A/B) over 1 min, 0:100 (A/B) for 3 min, then reversion back to 95:5 (A/B) for 3 min. This system was connected to a Shimadzu 9030 QTOF mass spectrometer (ESI ionisation) calibrated internally with Agilent's API-TOF reference mass solution kit (5.0 mM purine, 100.0 mM ammonium trifluoroacetate and 2.5 mM hexakis(1*H*,1*H*,3*H*-tetrafluoropropoxy)phosphazine) diluted to achieve a mass count of 10000.

Synthesis schemes, methods and analytical data on thiols and disulfide containing small molecules

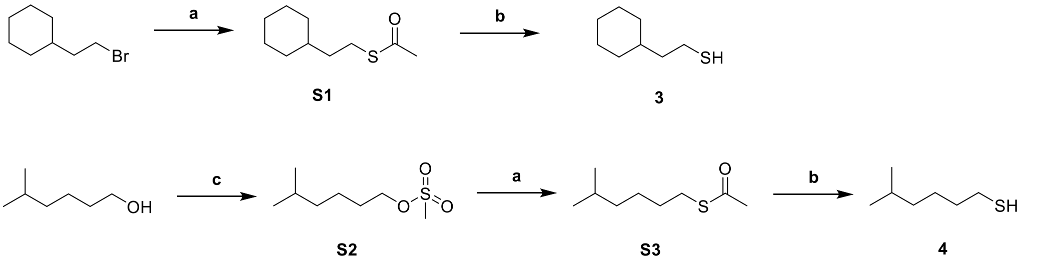

Scheme S1: Synthesis of aliphatic thiols used for subsequent disulfide construction. Reagents and conditions: (a) potassium thioacetate, DMF, 0 °C – RT, 1h; (b) NaOH (aq.), EtOH, 0 °C – RT, 2h; (c) Et_3N, methanesulfonyl chloride, DCM, 0 °C – RT, o/n.

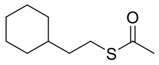

S1

S-(2-Cyclohexylethyl) ethanethioate

S1: 1-Bromo-2-cyclohexylethane (5.0 g, 26 mmol) was mixed with DMF (175 mL). The solution was cooled on ice. Potassium thioacetate 9.0 g, 79 mmol) was added to the mixture in one portion. The mixture was stirred at RT for 0.5 h. Once the reaction was complete, the mixture was diluted with DCM (250 mL), washed with cold water (3 x 250 mL) and brine. After drying (Na_2SO_4), the mixture was concentrated and column purified (2% EtOAc/PE) to yield the title compound. Yield: 4.17 g, 22.4 mmol, 86%. NMR: ^{1}H NMR (500 MHz, $CDCl_3$) δ 2.90 – 2.84 (m, 2H), 2.30 (s, 3H), 1.75 – 1.60 (m, 5H), 1.44 (ddd, J = 9.3, 7.7, 6.5 Hz, 2H), 1.35 – 1.26 (m, 1H), 1.26 – 1.10 (m, 3H), 0.95 – 0.83 (m, 2H). ^{13}C NMR (126 MHz, $CDCl_3$) δ 196.2, 37.2, 37.0, 33.0, 30.8, 27.0, 26.7, 26.3. HRMS: calculated for $C_{10}H_{19}OS$ $[M+H]^+$: 187.1158, found: 187.1153.

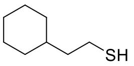

3

2-Cyclohexylethane-1-thiol

3: *S*-(2-Cyclohexylethyl) ethanethioate **[S1]** (1.0 g, 5.4 mmol) was mixed with EtOH (6 mL). The sample was cooled on ice and brought under N_2 atmosphere. Aqueous NaOH (5 M, 6 mL) was added

dropwise at 0 °C and the resulting mixture was stirred at RT for 1.5 hours. Once complete, the solution was acidified by aqueous HCl (1 M) and extracted with DCM (3 x 30 mL). The organic layers were combined, washed by brine, dried (Na_2SO_4) and concentrated on the rotovap. The crude compound, containing traces of DCM, was used without further purification for disulfide synthesis.

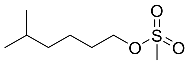

S2

5-Methylhexyl methanesulfonate

S2: 5-Methyl hexanol (0.5 g, 4.3 mmol) and Et_3N (0.96 mL, 6.9 mmol) were mixed with DCM (5.2 mL) and the solution was cooled to 0 °C. Methanesulfonyl chloride (0.55 g, 4.8 mmol) was added dropwise to the solution. The reaction was run for 0.5 h at 0 °C, followed by reaction at RT overnight. Mixture was washed with aqueous HCl (1 M, 3 x 5 mL), aqueous $NaHCO_3$ (sat., 3 x 5 mL) and brine (1 x 5 mL). The $NaHCO_3$ and brine layers were combined and back-extracted with DCM (20 mL). The combined DCM layers were dried (Na_2SO_4) and evaporated to yield the title compound. Yield: 0.73 g, 3.7 mmol, 87%. NMR: ^{1}H NMR (500 MHz, $CDCl_3$) δ 4.12 (t, J = 6.5, 2H), 2.91 (s, 3H), 1.69 – 1.59 (m, 2H), 1.51 – 1.41 (m, 1H), 1.36 – 1.27 (m, 2H), 1.17 – 1.08 (m, 2H), 0.79 (d, J = 7.6, 6H). ^{13}C NMR (126 MHz, $CDCl_3$) δ 70.3, 38.1, 37.0, 29.2, 27.7, 23.1, 22.3.

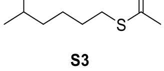

S3

***S*-(5-Methylhexyl) ethanethioate**

S3: 5-Methylhexyl methanesulfonate **[S2]** (0.68 g, 3.5 mmol) was dissolved in dry DMF (20 mL). The solution was cooled on ice to 0 °C and potassium thioacetate (1.2 g, 11 mmol) was added to the mixture in one portion. The mixture was stirred at RT for 1 h. Once complete, the mixture was diluted with DCM (60 mL) and washed with cold water (3 x 60 mL). The organic layer was dried (Na_2SO_4) and concentrated on the rotovap. Sample was column purified (2% EtOAc/PE) to yield the title compound. Yield: 0.42 g, 2.41 mmol, 69%. NMR: ^{1}H NMR (400 MHz, $CDCl_3$) δ 2.83 (t, J = 7.3 Hz, 2H),

2.28 (s, 3H), 1.59 – 1.39 (m, 3H), 1.37 – 1.24 (m, 2H), 1.19 – 1.08 (m, 2H), 0.83 (d, J = 6.7 Hz, 6H). ^{13}C NMR (101 MHz, $CDCl_3$) δ 196.0, 38.5, 30.7, 29.8, 29.2, 27.9, 26.7, 22.6. HRMS: calculated for $C_9H_{19}OS$ $[M+H]^+$: 175.1158, found: 175.1152.

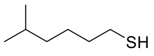

4

5-Methylhexane-1-thiol

4: *S*-(5-Methylhexyl) ethanethioate **[S3]** (0.41 g, 2.3 mmol) was dissolved in ethanol (3 mL). The mixture was cooled on ice and N_2 flushed. Aqueous NaOH (5 M, 3.0 mL) was added dropwise to the sample on ice. The ice bath was removed, and reaction was run at RT for 2 hours. Once complete, the solution was acidified with aqueous HCl (1 M). The mixture was extracted with DCM (3 x 30 mL), the combined organic layers dried (Na_2SO_4) and concentrated on the rotovap. The compound was taken straight for disulfide synthesis.

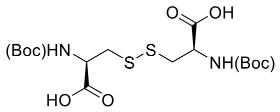

5a

(2*R*,2'*R*)-3,3'-Disulfanediylbis(2-((*tert*-butoxycarbonyl)amino)propanoic acid)

5a: This compound was made as described previously [1]. L-Cystine (5.0 g, 21 mmol) was dissolved in a water/THF mixture (9/1, 50 mL). The pH of the solution was adjusted to 12 with aqueous NaOH (6 M). Di-*tert*butyl dicarbonate (11.4 g, 52.2 mmol) was dissolved in THF (16 mL) and added dropwise to the stirred cystine solution. The reaction was stirred overnight at RT. Once complete, the mixture was acidified by aqueous HCl (1 M) to pH 2 and extracted with EtOAc (3 x 100 mL). Combined organic layers were washed by aqueous HCl (0.01 M) (2 x 50 mL) and brine (150 mL). The organic layer was dried ($NaSO_4$) and concentrated. The formed white solid was washed with *n*-hexane (2x) to obtain the title compound. Yield: 8.66 g, 19.7 mmol, 95%.

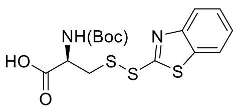

6a

S-(Benzo[d]thiazol-2-ylthio)-N-(tert-butoxycarbonyl)-L-cysteine

6a: *N*,*N'*-diBoc protected cystine **[5a]** (4.40 g, 10 mmol) was dissolved in THF (80 mL) and brought under N_2 atmosphere. Triphenylphosphine (3.9 g, 15 mmol) was added, followed by the addition of H_2O (8.8 mL). The reaction was heated to 50 °C and run overnight. Aqueous saturated $NaHCO_3$ was added (160 mL) after cooling the mixture. The mixture was extracted with EtOAc (3 x 100 mL). The combined EtOAc layers were back-extracted with cold water. The combined aqueous layers were cooled on ice and the pH was adjusted to 2 with aqueous HCl (1 M). The acidified layer was extracted with EtOAc (3 x 75 mL). The combined organic layers were dried (Na_2SO_4) and concentrated to yield (*tert*-butoxycarbonyl)-L-cysteine. Yield: 4.15g, 18.8 mmol, 94%.

2,2'-Dithiobis(benzothiazole) (0.67 gram, 2.0 mmol) was suspended in $CHCl_3$ (30 mL) and brought under N_2 atmosphere. (*tert*-butoxycarbonyl)-L-cysteine (0.44 g, 2.0 mmol) was dissolved in $CHCl_3$ (10 mL), brought under N_2 atmosphere and added slowly to the suspension of 2,2'-dithiobis(benzothiazole). The reaction was stirred vigorously at RT for 3 hours. The crude mixture was column purified (DCM - 5% MeOH/DCM/AcOH). Yield: 0.62 g, 1.6 mmol, 80%. NMR: ^{1}H NMR (500 MHz, $CDCl_3$). δ 7.93 (d, *J* = 8.2 Hz, 1H), 7.79 (d, *J* = 7.6 Hz, 1H), 7.47 (dt, *J* = 7.2, 1.2 Hz, 1H), 7.38 (dt, *J* = 7.4, 1.2 Hz, 1H), 5.90 (d, *J* = 7.1 Hz, 1H), 4.61 (td, *J* = 7.3, 4.7 Hz, 1H), 3.56 (dd, *J* = 14.4, 4.7 Hz, 1H), 3.24 (dd, *J* = 14.4, 7.7 Hz, 1H), 1.42 (s, 9H). ^{13}C NMR (126 MHz, $CDCl_3$) δ 172.2, 171.5, 155.2, 152.5, 135.7, 126.9, 125.5, 121.6, 121.6, 80.8, 53.7, 41.8, 28.4. HRMS: calculated for $C_{15}H_{19}N_2O_4S_3$ $[M+H]^+$: 387.0509, found: 387.0504.

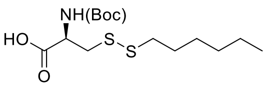

7a

N-(tert-Butoxycarbonyl)-S-(hexylthio)-L-cysteine

7a: *S*-(Benzo[*d*]thiazol-2-ylthio)-*N*-(*tert*-butoxycarbonyl)-L-cysteine **[6a]** (0.34 g, 0.9 mmol) was dissolved in $CHCl_3$ (30 mL) and brought under N_2 atmosphere. Hexanethiol (0.14 mL, 1.0 mmol) was

mixed with $CHCl_3$ (3 mL), brought under N_2 atmosphere and added slowly to the *S*-(Benzo[*d*]thiazol-2-ylthio)-*N*-(*tert*-butoxycarbonyl)-L-cysteine. After stirring overnight at RT under N_2, the mixture was concentrated and column purified (DCM – 5% MeOH/DCM/AcOH). Yield: 0.3 g, 0.9 mmol, 89%. ^{1}H NMR (500 MHz, $CDCl_3$/MeOD) δ 5.49 (d, *J* = 7.9 Hz, 1H), 4.57 – 4.47 (m, 1H), 3.17 (dd, *J* = 13.9, 4.6 Hz, 1H), 3.05 (dd, *J* = 13.9, 6.5 Hz, 1H), 2.69 – 2.64 (m, 2H), 1.62 (quintet, *J* = 7.4 Hz, 2H), 1.38 (s, 9H), 1.33 (quintet, *J* = 7.1 Hz, 2H), 1.29 – 1.20 (m, 4H), 0.84 (t, *J* = 7.0 Hz, 3H). ^{13}C NMR (126 MHz, $CDCl_3$/MeOD) δ 173.2, 155.6, 80.3, 53.0, 41.1, 39.0, 31.4, 29.1, 28.3, 28.2, 22.6, 14.0. HRMS: calculated for $C_{14}H_{28}NO_4S_2$ $[M+H]^+$: 338.1461, found: 338.1457.

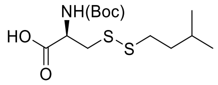

8a

_N_-(_tert_-Butoxycarbonyl)-_S_-(isopentylthio)-L-cysteine

8a: *S*-(Benzo[*d*]thiazol-2-ylthio)-*N*-(*tert*-butoxycarbonyl)-L-cysteine **[6a]** (0.07 g, 0.18 mmol) was dissolved in $CHCl_3$ (7 mL) and brought under N_2 atmosphere. 3-Methylbutanethiol (0.06 mL, 0.5 mmol) was mixed with $CHCl_3$ (1 mL), brought under N_2 atmosphere and added slowly to the *S*-(Benzo[*d*]thiazol-2-ylthio)-*N*-(*tert*-butoxycarbonyl)-L-cysteine. After stirring overnight at RT, the mixture was concentrated and column purified (DCM – 5% MeOH/DCM/AcOH). Yield: 0.05 g, 0.14 mmol, 79%. NMR: ^{1}H NMR (500 MHz, $CDCl_3$) δ 8.51 (s, 1H), 5.40 (d, *J* = 7.7 Hz, 1H), 4.65 – 4.45 (m, 1H), 3.24 – 3.07 (m, 2H), 2.71 (t, *J* = 7.7 Hz, 2H), 1.67 (n, *J* = 6.6 Hz, 1H), 1.59 – 1.51 (m, 2H), 1.45 (s, 9H), 0.90 (d, *J* = 6.6 Hz, 6H). ^{13}C NMR (126 MHz, $CDCl_3$) δ 175.5, 155.7, 80.8, 53.1, 40.5, 38.2, 37.1, 28.4, 27.3, 22.4. HRMS: calculated for $C_{13}H_{26}NO_4S_2$ $[M+H]^+$: 324.1305, found: 324.1303.

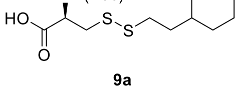

9a

_N_-(_tert_-Butoxycarbonyl)-_S_-((2-cyclohexylethyl)thio)-L-cysteine

9a: *S*-(Benzo[*d*]thiazol-2-ylthio)-*N*-(*tert*-butoxycarbonyl)-L-cysteine **[6a]** (0.19 g, 0.48 mmol) was dissolved in CH_3Cl (18 mL) and brought under N_2 atmosphere. 2-Cyclohexylethane-1-thiol (**3**, crude,

155 mg, ca. 1.1 mmol) was added dropwise to the reaction mixture. The reaction mixture was stirred at RT under N_2 overnight. Once complete, the reaction mixure was concentrated and column purified (2.5% MeOH/DCM – 5% MeOH/DCM/AcOH) to yield the title compound. Yield: 0.11 g, 0.30 mmol, 62%. NMR: ^{1}H NMR (500 MHz, $CDCl_3$) δ 5.40 (d, *J* = 7.6 Hz, 1H), 4.60 (s, 1H), 3.23 –3.16 (m, 1H), 3.16 – 3.07 (m, 1H), 2.72 (t, *J* = 7.6 Hz, 2H), 1.74 – 1.60 (m, 5H), 1.55 (q, *J* = 7.2 Hz, 2H), 1.45 (s, 9H), 1.40 – 1.28 (m, 1H), 1.29 – 1.09 (m, 3H), 0.96 – 0.84 (m, 2H). ^{13}C NMR (126 MHz, $CDCl_3$) δ 175.4, 155.7, 80.8, 53.2, 40.6, 36.8, 36.8, 33.2, 28.5, 26.7, 26.3. HRMS: calculated for $C_{16}H_{30}NO_4S_2$ $[M+H]^+$: 364.1618, found: 364.1614.

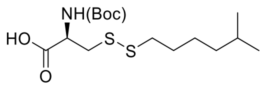

10a

_N_-(_tert_-Butoxycarbonyl)-_S_-((5-methylhexyl)thio)-L-cysteine

10a: *S*-(Benzo[*d*]thiazol-2-ylthio)-*N*-(*tert*-butoxycarbonyl)-L-cysteine **[6a]** (0.2 g, 0.5 mmol) was dissolved in $CHCl_3$ (18 mL) and brought under N_2 atmosphere. 5-Methylhexane-1-thiol (**4**, crude, 159 mg, ca. 1.2 mmol) was added dropwise to the reaction mixture. Mixture was stirred at RT overnight. Once complete, the reaction mixture was concentrated and column purified (2% MeOH/DCM - 5% MeOH/DCM/0.2% AcOH) to yield the title compound. Yield: 0.16 g, 0.52 mmol, 85%. NMR: ^{1}H NMR (500 MHz, $CDCl_3$) δ 7.92 (s, 1H), 5.37 (d, *J* = 6.9 Hz, 1H), 4.72 – 4.43 (m, 1H), 3.24 – 3.05 (m, 2H), 2.71 (t, *J* = 7.4 Hz, 2H), 1.65 (quintet, *J* = 7.5 Hz, 2H), 1.53 (dt, *J* = 13.3, 6.7 Hz, 1H), 1.46 (s, 9H), 1.41 – 1.32 (m, 2H), 1.21 – 1.15 (m, 2H), 0.87 (d, *J* = 6.6 Hz, 6H). ^{13}C NMR (126 MHz, $CDCl_3$) δ 175.4, 155.7, 80.8, 53.1, 40.5, 39.1, 38.6, 29.5, 28.4, 28.0, 26.4, 22.7. HRMS: calculated for $C_{15}H_{30}NO_4S_2$ $[M+H]^+$: 352.1618, found: 352.1614.

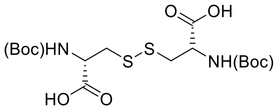

5b

(2_S_,2'_S_)-3,3'-Disulfanediylbis(2-((_tert_-butoxycarbonyl)amino)propanoic acid)

5b: Compound was prepared with a similar procedure to that employed for the synthesis of compound [**5a**]. Yield: 8.5 g, 19.2 mmol, 94%.

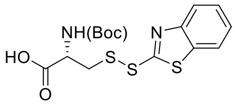

6b

S-(Benzo[*d*]thiazol-2-ylthio)-*N*-(*tert*-butoxycarbonyl)-D-cysteine

6b: Compound was prepared with a similar procedure to that employed for the synthesis of compound [**6a**] starting from (2*S*,2'*S*)-3,3'-disulfanediylbis(2-((*tert*-butoxycarbonyl)amino)-propanoic acid) **[5b]**. Yield: 3.3 gram, 8.4 mmol, 78%. NMR: ^{1}H NMR (400 MHz, $CDCl_3$) δ 7.94 (d, *J* = 8.2 Hz, 1H), 7.80 (d, *J* = 8.0 Hz, 1H), 7.48 (td, *J* = 7.8, 1.3 Hz, 1H), 7.38 (td, *J* = 7.7, 1.3 Hz, 1H), 5.90 (d, *J* = 7.0 Hz, 1H), 4.63 – 4.55 (m, 1H), 3.56 (dd, *J* = 14.3, 4.7 Hz, 1H), 3.21 (dd, *J* = 14.4, 8.0 Hz, 1H), 1.42 (s, 9H). ^{13}C NMR (101 MHz, $CDCl_3$) δ 172.1, 171.4, 155.2, 152.3, 135.7, 127.0, 125.6, 121.6, 80.9, 53.8, 41.8, 28.4. HRMS: calculated for $C_{15}H_{19}N_2O_4S_3$ $[M+H]^+$: 387.0509, found: 387.0509.

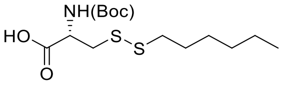

7b

N-(*tert*-Butoxycarbonyl)-*S*-(hexylthio)-D-cysteine

7b: Compound was prepared with a similar procedure to that employed for the synthesis of compound [**7a**] starting from **[6b]**. Yield: 0.33 g, 0.98 mmol, 98%. NMR: ^{1}H NMR (400 MHz, $CDCl_3$) δ 5.37 (d, *J* = 7.8 Hz, 1H), 4.68 – 4.56 (m, 1H), 3.25 – 3.08 (m, 2H), 2.71 (t, *J* = 7.4 Hz, 2H), 1.66 (quintet, *J* = 7.4 Hz, 2H), 1.46 (s, 9H), 1.43 – 1.22 (m, 6H), 0.89 (t, *J* = 6.8 Hz, 3H). ^{13}C NMR (101 MHz, $CDCl_3$) δ 175.6, 155.6, 80.8, 53.2, 40.6, 39.1, 31.6, 29.2, 28.5, 28.3, 22.7, 14.2. HRMS: calculated for $C_{14}H_{28}NO_4S_2$ $[M+H]^+$: 338.1461, found: 338.1457.

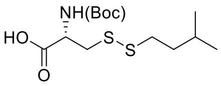

8b

N-(*tert*-Butoxycarbonyl)-*S*-(isopentylthio)-D-cysteine

8b: Compound was prepared with a similar procedure to that employed for the synthesis of compound [**8a**] starting from **[6b]**. Yield: 0.25 g, 0.78 mmol, 78%. NMR: ^{1}H NMR (400 MHz, $CDCl_3$) δ 9.01 (s, 1H), 5.37 (d, *J* = 7.1 Hz, 1H), 4.72 – 4.56 (m, 1H), 3.33 – 3.06 (m, 2H), 2.72 (t, *J* = 7.7 Hz, 2H),

1.75 – 1.60 (m, 1H), 1.55 (q, *J* = 7.6 Hz, 2H), 1.46 (s, 9H), 0.91 (dd, *J* = 6.6, 1.5 Hz, 6H). ^{13}C NMR (101 MHz, $CDCl_3$) δ 175.5, 155.7, 80.8, 53.2, 40.6, 38.2, 37.2, 28.4, 27.4, 22.4. HRMS: calculated for $C_{13}H_{26}NO_4S_2$ $[M+H]^+$: 324.1305, found: 324.1301.

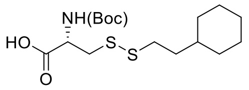

9b

***N*-(*tert*-Butoxycarbonyl)-*S*-((2-cyclohexylethyl)thio)-D-cysteine**

9b: Compound was prepared with a similar procedure to that employed for the synthesis of compound [**9a**] starting from **[6b]**. Yield: 0.22 g, 0.54 mmol, 89%. NMR: ^{1}H NMR (500 MHz, $CDCl_3$) δ 5.36 (d, *J* = 7.6 Hz, 1H) 4.66 – 4.56 (m, 1H), 3.24 – 3.07 (m, 2H), 2.73 (t, *J* = 7.7 Hz, 2H), 1.74 – 1.67 (m, 4H), 1.67 – 1.60 (m, 1H), 1.59 – 1.51 (m, 2H), 1.46 (s, 9H), 1.40 – 1.30 (m, 1H), 1.26 – 1.09 (m, 3H), 0.97 – 0.85 (m, 2H). ^{13}C NMR (126 MHz, $CDCl_3$) δ 175.2, 155.7, 80.9, 53.1, 40.4, 36.8, 36.8, 33.2, 28.4, 26.7, 26.3. HRMS: calculated for $C_{16}H_{30}NO_4S_2$ $[M+H]^+$: 364.1618, found: 364.1614.

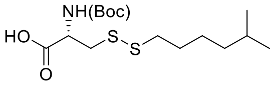

10b

***N*-(*tert*-Butoxycarbonyl)-*S*-((5-methylhexyl)thio)-D-cysteine**

10b: Compound was prepared with a similar procedure to that employed for the synthesis of compound [**10a**] starting from **[6b]**. Yield: 0.15 g, 81%. NMR: ^{1}H NMR (500 MHz, $CDCl_3$) δ 5.47 (s, 1H), 4.54 (s, 1H), 3.28 – 3.03 (m, 2H), 2.70 (t, *J* = 7.4 Hz, 2H), 1.64 (quint., *J* = 7.5 Hz, 2H), 1.53 (n, *J* = 6.7 Hz, 1H), 1.45 (s, 9H), 1.40 – 1.33 (m, 2H), 1.22 – 1.13 (m, 2H), 0.86 (d, *J* = 6.6 Hz, 6H). ^{13}C NMR (126 MHz, $CDCl_3$) δ 172.0, 155.8, 80.6, 53.5, 40.8, 39.0, 38.6, 29.4, 28.5, 28.0, 26.4, 22.7. HRMS: calculated for $C_{15}H_{30}NO_4S_2$ $[M+H]^+$: 352.1618, found: 352.1615.

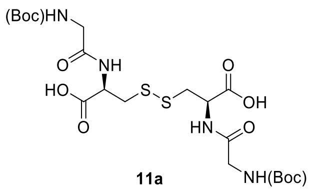

11a

(9*R*,14*R*)-14-(2-((*tert*-Butoxycarbonyl)amino)acetamido)-9-carboxy-2,2-dimethyl-4,7-dioxo-3-oxa-11,12-dithia-5,8-diazapentadecan-15-oic acid

11a: $Na_2CO_3 \cdot 10\ H_2O$ (4.9 g, 0.17 mmol) and L-cystine (1.2 g, 5.0 mmol) were dissolved in water (18 mL) and THF (8 mL) and cooled on ice. 2,5-Dioxopyrrolidin-1-yl (*tert*-butoxycarbonyl)glycinate (3.1g, 11.5 mmol) was dissolved in THF (18 mL) and added dropwise to the above mixture. The reaction was stirred overnight, concentrated under reduced pressure and quenched with water (100 mL). The mixture was extracted with DCM (2 x 40 mL). The aqueous layer was acidified with aqueous citric acid (5% w/v) and extracted with EtOAc (3 x 40 mL). The aqueous layer was further acidified to pH 1 with aqueous HCl (5 M) and extracted with EtOAc (3 x 60 mL). The combined EtOAc layers were dried (Na_2SO_4) and evaporated to yield the title compound. Yield: 2.0 g, 3.6 mmol, 73%. NMR: ^1H NMR (500 MHz, DMSO-*d6*) δ 8.21 (d, *J* = 8.0 Hz, 2H), 6.94 (t, *J* = 6.2 Hz, 2H), 4.65 – 4.47 (m, 2H), 3.64 – 3.59 (m, 4H), 3.14 (dd, *J* = 13.8, 4.9 Hz, 2H), 2.95 (dd, *J* = 13.7, 8.6 Hz, 2H), 1.38 (s, 18H). ^{13}C NMR (126 MHz, $CDCl_3$) δ 171.8, 169.5, 155.8, 78.1, 51.3, 42.9, 39.5, 28.2. HRMS: calculated for $C_{20}H_{35}N_4O_{10}S_2$ $[M+H]^+$: 555.1796, found: 555.1794.

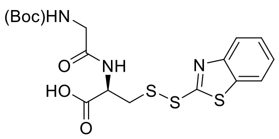

12a

***S*-(Benzo[*d*]thiazol-2-ylthio)-*N*-((*tert*-butoxycarbonyl)glycyl)-L-cysteine**

12a: Compound was prepared with a similar procedure to that employed for the synthesis of [**6a**] starting from **[11a]** and column purified (DCM – 10% MeOH/DCM/AcOH). Yield: 0.55 g, 1.2 mmol, 50%. NMR: ^1H NMR (400 MHz, $CDCl_3$) δ 7.88 (d, *J* = 8.2 Hz, 1H), 7.79 (d, *J* = 8.0 Hz, 1H), 7.48 (s, 1H), 7.46 (t, *J* = 7.7 Hz, 1H), 7.35 (t, *J* = 7.7 Hz, 1H), 5.52 – 5.34 (m, 1H), 4.81 – 5.01 (m, 1H), 3.93 (dd, *J* = 17.1, 6.1 Hz, 1H), 3.82 (dd, *J* = 17.2, 5.7 Hz, 1H), 3.54 – 3.31 (m, 2H), 1.44 (s, 9H). ^{13}C NMR (101 MHz,

$CDCl_3$) δ 171.9, 171.8, 170.0, 156.5, 153.4, 135.8, 126.8, 125.4, 121.9, 121.5, 80.8, 52.4, 44.2, 41.0, 28.4. HRMS: calculated for $C_{17}H_{22}N_3O_5S_3$ $[M+H]^+$: 444.0723, found: 444.072.

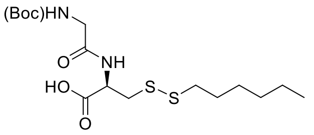

13a

N-((tert-Butoxycarbonyl)glycyl)-S-(hexylthio)-L-cysteine

13a: Compound was prepared with a similar procedure to that employed for the synthesis of **[7a]**, starting from **[12a]** and column purified (DCM – 7.5% MeOH/DCM/AcOH). Yield: 0.21 g, 0.52 mmol, 87%. NMR: ^1H NMR (400 MHz, $CDCl_3$) δ 7.15 (s, 1H), 5.36 (s, 1H), 4.88 (s, 1H), 4.00 (dd, *J* = 17.1, 6.7 Hz, 1H), 3.82 (d, *J* = 15.1 Hz, 1H), 3.25 (d, *J* = 14.6 Hz, 1H), 3.13 (dd, *J* = 14.1, 6.4 Hz, 1H), 2.70 (t, *J* = 7.3 Hz, 2H), 1.66 (quint., *J* = 7.3 Hz, 2H), 1.46 (s, 9H), 1.42 – 1.34 (m, 2H), 1.34 – 1.21 (m, 4H), 0.89 (t, *J* = 6.2 Hz, 3H). ^{13}C NMR (101 MHz, $CDCl_3$) δ 173.2, 170.4, 156.6, 80.8, 52.2, 44.1, 40.0, 39.0, 31.5, 29.1, 28.4, 28.3, 22.7, 14.2. HRMS: calculated for $C_{16}H_{31}N_2O_5S_2$ $[M+H]^+$: 395.1676, found: 395.1675.

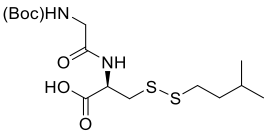

14a

N-((tert-Butoxycarbonyl)glycyl)-S-(isopentylthio)-L-cysteine

14a: Compound was prepared with a similar procedure to that employed for the synthesis of **[8b]**, starting from **[12a]** and column purified (DCM – 7.5% MeOH/DCM/AcOH). Yield: 0.17 g, 0.42 mmol, 71%. NMR: ^1H NMR (400 MHz, $CDCl_3$) δ 10.02 (s, 1H), 7.54 – 7.28 (m, 1H), 5.61 (s, 1H), 4.99 – 4.79 (m, 1H), 3.96 (dd, *J* = 16.6, 5.8 Hz, 1H), 3.89 – 3.72 (m, 1H), 3.37 – 3.17 (m, 1H), 3.10 (dd, *J* = 14.1, 6.8 Hz, 1H), 2.69 (t, *J* = 7.7, 2H), 1.71 – 1.59 (m, 1H), 1.52 (q, *J* = 7.1 Hz, 2H), 1.44 (s, 9H), 0.89 (d, *J* = 6.6 Hz, 6H). ^{13}C NMR (101 MHz, $CDCl_3$) δ 173.1, 170.5, 156.6, 80.7, 52.2, 44.1, 40.0, 38.2, 37.0, 28.4, 27.3, 22.4. HRMS: calculated for $C_{15}H_{29}N_2O_5S_2$ $[M+H]^+$: 381.152, found: 381.1519.

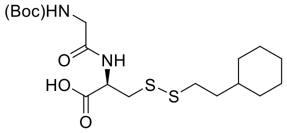

15a

N-((*tert*-Butoxycarbonyl)glycyl)-S-((2-cyclohexylethyl)thio)-L-cysteine

15a: Compound was prepared with a similar procedure to that employed for the synthesis of **[9a]** starting from **[12a]** and column purified (DCM – 7.5% MeOH/DCM/AcOH). Yield: 0.16 g, 0.37 mmol, 61%. NMR: ^{1}H NMR (500 MHz, $CDCl_3$) δ 7.20 (s, 1H), 5.43 (s, 1H), 5.05 – 4.87 (s, 1H), 4.00 (dd, J = 17.2, 6.6 Hz, 1H), 3.82 (d, J = 17.2 Hz, 1H), 3.23 (d, J = 13.8 Hz, 1H), 3.12 (dd, J = 14.1, 6.5 Hz, 1H), 2.72 (t, J = 7.7 Hz, 2H), 1.74 – 1.61 (m, 5H), 1.57 – 1.51 (m, 2H), 1.45 (s, 9H), 1.38 – 1.29 (m, 1H), 1.27 – 1.08 (m, 3H), 0.95 – 0.84 (m, 2H). ^{13}C NMR (126 MHz, $CDCl_3$) δ 173.1, 170.3, 156.6, 80.9, 52.2, 44.1, 39.9, 36.8, 36.7, 36.7, 33.2, 28.5, 26.7, 26.3. HRMS: calculated for $C_{18}H_{33}N_2O_5S_2$ $[M+H]^+$: 421.1833, found: 421.1830.

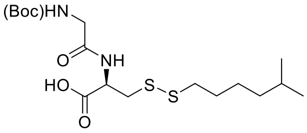

16a

N-((*tert*-Butoxycarbonyl)glycyl)-S-((5-methylhexyl)thio)-L-cysteine

16a: Compound was prepared with a similar procedure to that employed for the synthesis of **[10a]** starting from **[12a]** and column purified (DCM – 7.5% MeOH/DCM/AcOH). Yield: 35 mg, 0.09 mmol, 19%. NMR: ^{1}H NMR (400 MHz, $CDCl_3$). δ 7.50 – 7.30 (m, 1H), 5.61 (s, 1H), 4.95 – 4.67 (m, 1H), 4.04 – 3.74 (m, 2H), 3.31 – 3.16 (m, 1H), 3.09 (dd, J = 14.2, 7.0 Hz, 1H), 2.69 (t, J = 7.4 Hz, 2H), 1.63 (quintet, J = 7.5 Hz, 2H), 1.58 – 1.46 (m, 1H), 1.45 (s, 9H), 1.41 – 1.31 (m, 2H), 1.21 – 1.13 (m, 2H), 0.86 (d, J = 6.6 Hz, 6H). ^{13}C NMR (101 MHz, $CDCl_3$) δ 174.7, 170.7, 156.5, 80.7, 52.9, 44.2, 39.9, 38.9, 38.6, 29.4, 28.5, 28.0, 26.4, 22.7. HRMS: calculated for $C_{17}H_{33}N_2O_5S_2$ $[M+H]^+$: 409.1833, found: 409.1831.

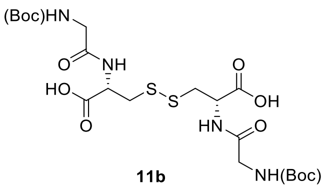

11b

(9*S*,14*S*)-14-(2-((*tert*-Butoxycarbonyl)amino)acetamido)-9-carboxy-2,2-dimethyl-4,7-dioxo-3-oxa-11,12-dithia-5,8-diazapentadecan-15-oic acid

11b: Compound was prepared with a similar procedure to that employed for the synthesis of **[11a]** starting from commercially available D-cystine. NMR: ^{1}H NMR (500 MHz, $CDCl_3$) δ 8.16 (d, *J* = 8.00 Hz, 1H), 6.91 (t, *J* = 6.1 Hz, 1H), 4.50 (m, 2H), 3.52-3.63 (m, 4H), 3.14 (dd, *J* = 4.8, 13.8 Hz, 2H), 2.93 (dd, *J* = 8.4, 13.7 Hz, 2H), 1.37 (s, 18H). ^{13}C NMR (126 MHz, DMSO-*d6*) δ 172.0, 169.7, 155.9, 78.2, 51.5, 43.1, 39.7, 28.3. HRMS: calculated for $C_{20}H_{35}N_4O_{10}S_2$ $[M+H]^+$: 555.1796, found: 555.1796.

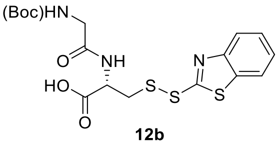

12b

***S*-(Benzo[*d*]thiazol-2-ylthio)-*N*-((*tert*-butoxycarbonyl)glycyl)-D-cysteine**

12b: Compound was prepared with a similar procedure to that employed for the synthesis of [**12a**] starting from **[11b]**. Yield: 0.28 g, 0.62 mmol, 35%. NMR: ^{1}H NMR (600 MHz, $CDCl_3$) δ 7.85 (d, *J* = 8.3 Hz, 1H), 7.76 (d, *J* = 8.0 Hz, 1H), 7.62 (s, 1H), 7.41 (t, *J* = 7.8 Hz, 1H), 7.31 (t, *J* = 7.7 Hz, 1H), 5.60 (s, 1H), 5.03 – 4.81 (m, 1H), 4.01 – 3.75 (m, 2H), 3.56 – 3.40 (m, 2H), 1.42 (s, 9H). ^{13}C NMR (151 MHz, $CDCl_3$) δ 172.2, 172.0, 170.4, 156.5, 153.9, 135.8, 126.7, 125.2, 122.0, 121.5, 80.8, 52.3, 44.1, 40.9, 28.4. HRMS: calculated for $C_{17}H_{22}N_3O_5S_3$ $[M+H]^+$: 444.0723, found: 444.0710.

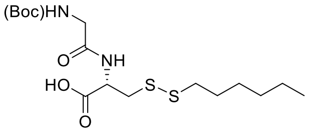

13b

***N*-((*tert*-Butoxycarbonyl)glycyl)-*S*-(hexylthio)-D-cysteine**

13b: Compound was prepared with a similar procedure to that employed for the synthesis of **[13a]** starting from **[12b]**. Yield: 0.11 g, 0.28 mmol, 96%. NMR: ^{1}H NMR (600 MHz, $CDCl_3$) δ 9.64 (s, 1H), 7.30 – 7.21 (m, 1H), 5.53 (s, 1H), 5.05 – 4.81 (m, 1H), 4.04 – 3.72 (m, 2H), 3.35 – 3.05 (m, 2H), 2.69

(t, J = 7.4 Hz, 2H), 1.65 (quintet, J = 7.2 Hz, 2H), 1.44 (s, 9H), 1.36 (quintet, J = 7.2 Hz, 2H), 1.33 – 1.23 (m, 4H), 0.88 (t, J = 6.9 Hz, 3H). ^{13}C NMR (151 MHz, $CDCl_3$) δ 173.2, 170.5, 156.6, 80.8, 52.2, 44.1, 40.0, 39.0, 31.5, 29.1, 28.5, 28.3, 22.7, 14.2. HRMS: calculated for $C_{16}H_{31}N_2O_5S_2$ $[M+H]^+$: 395.1676, found: 395.1673.

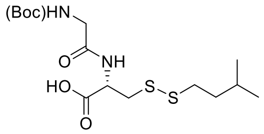

14b

_N_-((_tert_-Butoxycarbonyl)glycyl)-_S_-(isopentylthio)-D-cysteine

14b: Compound was prepared with a similar procedure to that employed for the synthesis of **[14a]** starting from **[12b]**. Yield: 0.10 g, 0.27 mmol, 94%. NMR: ^{1}H NMR (600 MHz, $CDCl_3$) δ 10.28 (s, 1H), 7.34 – 7.27 (m, 1H), 5.58 (s, 1H), 5.00 – 4.80 (m, 1H), 4.03 – 3.73 (m, 2H), 3.30 – 3.07 (m, 2H), 2.70 (t, J = 7.4 Hz, 2H), 1.66 (n, J = 6.8 Hz, 1H), 1.53 (q, J = 7.1 Hz, 2H), 1.45 (s, 9H), 0.89 (d, J = 6.7 Hz, 6H). ^{13}C NMR (151 MHz, $CDCl_3$) δ 173.2, 170.5, 156.6, 80.8, 52.2, 44.1, 40.0, 38.2, 37.1, 28.4, 27.3, 22.4. HRMS: calculated for $C_{15}H_{29}N_2O_5S_2$ $[M+H]^+$: 381.152, found: 381.1517.

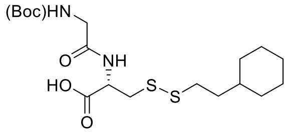

15b

_N_-((_tert_-Butoxycarbonyl)glycyl)-_S_-((2-cyclohexylethyl)thio)- D-cysteine

15b: Compound was prepared with a similar procedure to that employed for the synthesis of **[15a]** starting from **[12b]**. Yield: 0.16 g, 0.37 mmol, 61%. NMR: ^{1}H NMR (500 MHz, $CDCl_3$) δ 7.21 – 7.09 (m, 1H), 5.39 (s, 1H), 5.05 – 4.83 (m, 1H), 4.01 (dd, J = 17.1, 6.6 Hz, 1H), 3.88 – 3.75 (m, 1H), 3.31 – 3.20 (m, 1H), 3.13 (dd, J = 14.1, 6.4 Hz, 1H), 2.72 (t, J = 7.7 Hz, 2H), 1.74 – 1.61 (m, 5H), 1.60 – 1.51 (m, 2H), 1.45 (s, 9H), 1.39 – 1.29 (m, 1H), 1.27 – 1.08 (m, 3H), 0.95 – 0.85 (m, 2H). ^{13}C NMR (126 MHz, $CDCl_3$) δ 173.0, 170.3, 156.6, 81.0, 52.2, 44.2, 39.9, 36.8, 36.7, 36.7, 33.2, 28.4, 26.7, 26.3. HRMS: calculated for $C_{18}H_{33}N_2O_5S_2$ $[M+H]^+$: 421.1833, found: 421.1831.

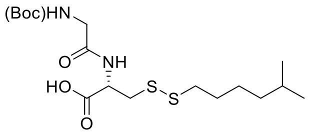

16b

_N_-((_tert_-Butoxycarbonyl)glycyl)-_S_-((5-methylhexyl)thio)-D-cysteine

16b: Compound was prepared with a similar procedure to that employed for the synthesis of **[16a]** starting from **[12b]**. Yield: 0.13 g, 0.31 mmol, 69%. NMR: ^{1}H NMR (500 MHz, $CDCl_3$) δ 7.27 – 7.19 (m, 1H), 5.45 (s, 1H), 5.05 – 4.80 (m, 1H), 4.01 (dd, *J* = 17.2, 6.6 Hz, 1H), 3.88 – 3.74 (m, 1H), 3.31 – 3.17 (m, 1H), 3.12 (dd, *J* = 14.1, 6.5 Hz, 1H), 2.70 (t, *J* = 7.4 Hz, 2H), 1.68 – 1.60 (m, 2H), 1.57 – 1.51 (m, 1H), 1.45 (s, 9H), 1.40 – 1.33 (m, 2H), 1.22 – 1.13 (m, 2H), 0.87 (d, *J* = 6.6 Hz, 6H). ^{13}C NMR (126 MHz, $CDCl_3$) δ 173.0, 170.5, 156.6, 81.0, 52.2, 44.2, 39.9, 39.1, 38.6, 29.4, 28.4, 28.0, 26.4, 22.7. HRMS: calculated for $C_{15}H_{30}NO_4S_2$ $[M+H]^+$: 352.1618, found: 352.1615.

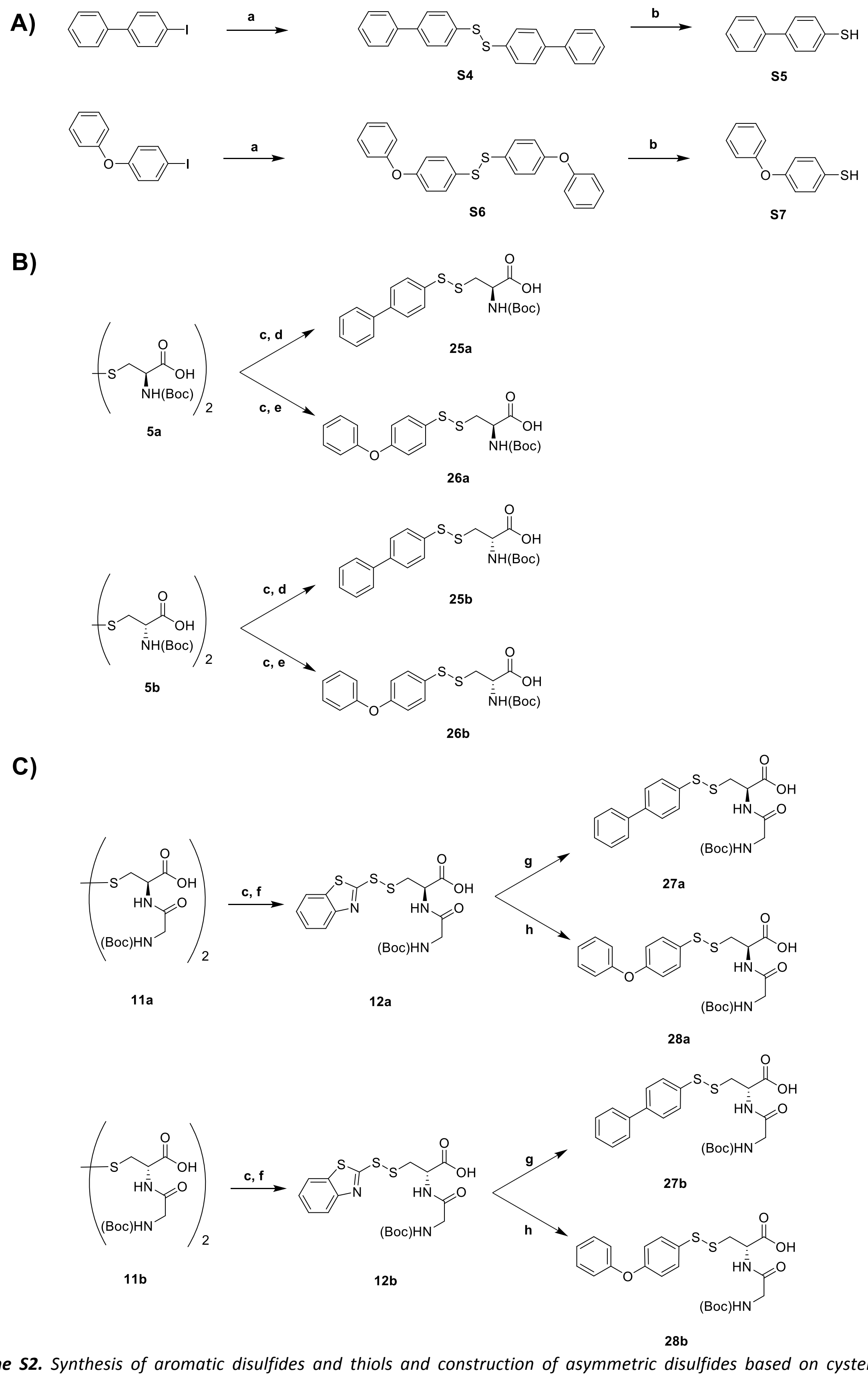

Scheme S2. *Synthesis of aromatic disulfides and thiols and construction of asymmetric disulfides based on cysteine. A) Synthesis of biphenyl disulfides and thiols. B) Conjugation of cysteine derived building blocks to biphenyl disulfides. C) Conjugation of cysteine derived building blocks to biphenyl thiols via benzothiazole intermediate. Reagents and conditions: (a) i) K_2CO_3, S_8, CuI, DMF, 90 ^oC, 36 h; ii) $NaBH_4$, 40 ^oC, 5 h; (b) Zn, HCl (aq), Et_2O, RT, 5 h; (c) PPh_3, H_2O, THF, 50 ^oC, o/n; (d)* ***S4****, Et_3N, $CHCl_3$/MeOH, RT, o/n; (e)* ***S6****, Et_3N, $CHCl_3$/MeOH, RT, o/n. (f) 2,2'-dithiobis(benzothiazole), $CHCl_3$, RT, 3h; (g)* ***S5****, $CHCl_3$, RT, o/n; (h)* ***S7****, $CHCl_3$, RT, o/n.*

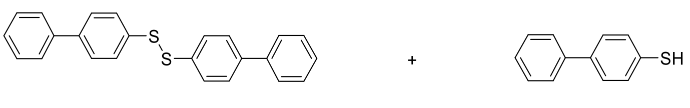

S4

1,2-Di([1,1'-biphenyl]-4-yl)disulfane and **[1,1'-biphenyl]-4-thiol**

S4: Compound was made as described before [2] with minor modifications. 4-Iodo-1,1'-biphenyl (5.0 g, 17.9 mmol), K_2CO_3 (4.93g, 35.7 mmol), S_8 (1.71 g, 53.6 mmol) and copper iodide (0.34 g, 1.79 mmol) were weighed out in an oven-dried flask, which was flushed with N_2. Dry DMF (38 mL) was added. The reaction was run at 90 °C for 36 hours. The flask was cooled on ice and $NaBH_4$ (2.03 g, 53.6 mmol) was added. The mixture was further kept at 40 °C for 5 hours. Aqueous HCl (3 M, 38 mL) was added to quench the reaction. Cold water (50 mL) was added and the mixture was extracted with EtOAc (3 x 200 mL). The EtOAc layer was filtered to remove residual sulfur. The organic layers were dried (Na_2SO_4), concentrated and column purified (15% DCM/PE) to yield a mixture of thiol species and symmetric disulfides. Yield: 2.1 g, 11.3 mmol, 64%.

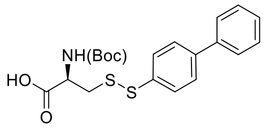

25a

***S*-([1,1'-Biphenyl]-4-ylthio)-*N*-(*tert*-butoxycarbonyl)-L-cysteine**

25a: Compound **[S4]** (0.11 g, 0.30 mmol) was dissolved in $CHCl_3$ (6 mL) and Et_3N (41 µL, 0.3 mmol) was added under N_2 atmosphere. (*tert*-Butoxycarbonyl)-L-cysteine (formed by the reduction of **[5a]** by triphenylphosphine as described for **[6a]**, 60 mg, 0.27 mmol) was dissolved in $CHCl_3$ (1 mL) and added slowly. The mixture was stirred at RT for 3 h. Afterwards, additional (*tert*-butoxycarbonyl)-L-cysteine (60 mg, 0.27 mmol) dissolved in MeOH (2 mL) was added and the reaction was kept at RT overnight. The mixture was evaporated completely, and the residue was dissolved in DCM and column purified (DCM - 2.5% MeOH/DCM/AcOH) to yield the title compound. Yield: 79 mg, 0.20 mmol, 65%. NMR: ^{1}H NMR (500 MHz, $CDCl_3$) δ 7.64 – 7.51 (m, 6H), 7.47 – 7.40 (m, 2H), 7.39 – 7.32 (m, 1H), 5.34 (d, *J* = 7.6 Hz, 1H), 4.75 – 4.47 (m, 1H), 3.35 – 3.15 (m, 2H), 1.44 (s, 9H). ^{13}C NMR (126

MHz, $CDCl_3$) δ 175.2, 155.6, 140.8, 140.3, 135.6, 129.3, 129.0, 128.0, 127.7, 80.9, 53.1, 40.4, 28.4. HRMS: calculated for $C_{20}H_{24}NO_4S_2$ $[M-H]^+$: 404.0988, found: 404.0999.

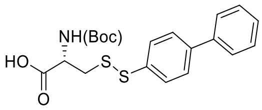

25b

_S_-([1,1'-Biphenyl]-4-ylthio)-_N_-(_tert_-butoxycarbonyl)-D-cysteine

25b: Compound **[S4]** (0.25 g, 0.68 mmol) was dissolved in $CHCl_3$ (7 mL) and brought under N_2 atmosphere. (*tert*-Butoxycarbonyl)-D-cysteine (formed by the reduction of **[5b]** by triphenylphosphine as described for **[6a]**, 130 mg, 0.59 mmol) was dissolved in MeOH (2 mL) and Et_3N (82 µL, 0.59 mmol) was added to it. The mixture was added slowly. The reaction was stirred at RT for 4 h. Once complete, DCM (10 mL) was added to the reaction mixture and the organics were washed by aqueous HCl (1 M, 2 x 5 mL) and water (2 x 5 mL). The aqueous layers were back-extracted by DCM. The combined organic layers were dried and column purified (DCM – 4% MeOH/DCM/AcOH) to yield the title compound. Yield: 97 mg, 0.24 mmol, 41%. NMR: ^1H NMR (500 MHz, $CDCl_3$) δ 7.63 – 7.52 (m, 6H), 7.47 – 7.40 (m, 2H), 7.39 – 7.32 (m, 1H), 5.32 (d, *J* = 7.3 Hz, 1H), 4.68 – 4.60(m, 1H), 3.29 (dd, *J* = 14.0, 4.5 Hz, 1H), 3.21 (dd, *J* = 6.1, 14.3 Hz, 1H), 1.44 (s, 9H). ^{13}C NMR (126 MHz, $CDCl_3$) δ 174.6, 155.6, 140.8, 140.3, 135.5, 129.3, 129.0, 128.0, 127.7, 127.1, 80.9, 53.0, 40.3, 28.4. HRMS: calculated for $C_{20}H_{24}NO_4S_2$ $[M+H]^+$: 406.1148, found: 406.1146.

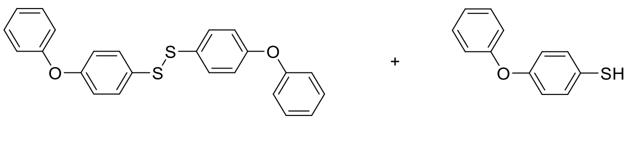

S6

1,2-Bis(4-phenoxyphenyl)disulfane and **4-phenoxybenzenethiol**

S6: Compound was made as described before for the analogues biphenyl species **[S4]**. 1-Iodo-4-phenoxybenzene (3.8 g, 12.8 mmol), K_2CO_3 (3.55 g, 25.7 mmol), S_8 (1.23 g, 38.5 mmol) and copper iodide (0.37 g, 1.92 mmol) were loaded into an oven-dried flask, which was flushed with N_2. Dry DMF

(45 mL) was added. The reaction was run at 90 °C for 36 hours. The flask was cooled on ice and $NaBH_4$ (1.46 g, 38.5 mmol) was added. The mixture was further kept at 40 °C for 5 hours. Aqueous HCl (3 M, 29 mL) was added to quench the reaction. Cold water (50 mL) was added and the mixture was extracted with EtOAc (3 x 150 mL). The EtOAc layer was filtered to remove residual sulfur. The organic layers were dried (Na_2SO_4), concentrated and column purified (15% DCM/PE) to yield a mixture of thiol species and symmetric disulfides. Yield: 2.1 g, 10.2 mmol, 80%.

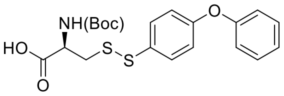

26a

***N*-(*tert*-Butoxycarbonyl)-*S*-((4-phenoxyphenyl)thio)-L-cysteine**

26a: Compound was prepared with a similar procedure to that employed for the synthesis of **[25a]** starting from **[S6]**. Yield: 0.13 g, 0.31 mmol, 45%. NMR: ^{1}H NMR (500 MHz, $CDCl_3$) δ 7.52 – 7.47 (m, 2H), 7.38 – 7.32 (m, 2H), 7.13 (t, *J* = 7.3 Hz, 1H), 7.05 – 7.00 (m, 2H), 6.97 – 6.92 (m, 2H), 5.34 (d, *J* = 6.9 Hz, 1H), 4.70 – 4.50 (m, 1H), 3.32 – 3.10 (m, 2H), 1.45 (s, 9H). ^{13}C NMR (126 MHz, $CDCl_3$) δ 175.4, 157.9, 156.6, 155.6, 132.1, 130.3, 130.0, 124.0, 119.5, 119.3, 80.9, 53.0, 40.3, 28.4. HRMS: calculated for $C_{20}H_{24}NO_5S_2$ $[M+H]^+$: 422.1098, found: 422.1093.

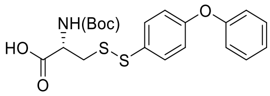

26b

***N*-(*tert*-Butoxycarbonyl)-*S*-((4-phenoxyphenyl)thio)-D-cysteine**

26b: Compound was prepared with a similar procedure to that employed for the synthesis of **[25b]** starting from **[S6]**. Yield: 74 mg, 0.18 mmol, 30%. NMR: ^{1}H NMR (500 MHz, $CDCl_3$) δ 7.52 – 7.47 (m, 2H), 7.39 – 7.32 (m, 2H), 7.17 – 7.11 (m, 1H), 7.05 – 7.00 (m, 2H), 6.98 – 6.93 (m, 2H), 5.30 (d, *J* = 7.0 Hz, 1H), 4.72 – 4.48 (m, 1H), 3.31 – 2.92 (m, 2H), 1.45 (s, 9H). ^{13}C NMR (126 MHz, $CDCl_3$) δ 175.0,

158.0, 156.6, 155.6, 132.2, 130.2, 130.0, 124.0, 119.5, 119.3, 80.9, 53.0, 40.2, 28.5. HRMS: calculated for $C_{20}H_{24}NO_5S_2$ $[M+H]^+$: 422.1098, found: 422.1092.

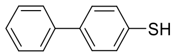

S5

[1,1'-Biphenyl]-4-thiol

S5: **[S4]** (370 mg, 1 mmol) was dissolved in Et_2O (10 mL). Zn dust (0.65 g, 10 mmol) was added and the mixture was cooled on ice. Aqueous HCl (5% v/v, 20 mL) was added and the reaction was run for 5 hours at RT. Aqueous HCl (37% v/v, 1 mL) was added and mixture was extracted with Et_2O (3 x 20 mL). The organic layers were combined, dried and concentrated to yield [1,1'-biphenyl]-4-thiol. Yield: 320 mg, 1.72 mmol, 86%.

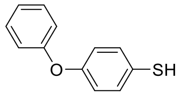

S7

4-Phenoxybenzenethiol

S7: Compound was prepared with a similar procedure to that employed for the synthesis of **[S5]** starting from **[S6]**. Yield: 355 mg, 1.76 mmol, 75%.

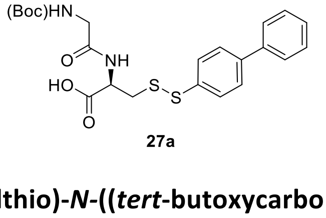

27a

***S*-([1,1'-Biphenyl]-4-ylthio)-*N*-((*tert*-butoxycarbonyl)glycyl)-L-cysteine**

27a: *S*-(Benzo[*d*]thiazol-2-ylthio)-*N*-((*tert*-butoxycarbonyl)glycyl)-L-cysteine **[12a]** (40 mg, 0.08 mmol) was suspended in $CHCl_3$ (5 mL) and brought under N_2 atmosphere. [1,1'-Biphenyl]-4-thiol **[S5]** (34 mg, 0.18 mmol) was dissolved in $CHCl_3$ (2 mL), and added slowly to the unsymmetic disulfide. Reaction was stirred at RT overnight under a N_2 atmosphere. Once complete, the mixture was concentrated and column purified (DCM - 5% MeOH/DCM/AcOH). Yield: 26 mg, 0.06 mmol, 62%. NMR: ^1H NMR

(500 MHz, MeOD/$CDCl_3$) δ 7.60 – 7.50 (m, 6H), 7.44 – 7.35 (m, 2H), 7.35 – 7.28 (m, 1H), 4.81 – 4.75 (m, 1H), 3.80 – 3.66 (m, 2H), 3.28 (dd, *J* = 14.1, 4.6 Hz, 1H), 3.13 (dd, *J* = 14.1, 7.3 Hz, 1H), 1.41 (s, 9H). ^{13}C NMR (126 MHz, MeOD/$CDCl_3$) δ 172.3, 170.8, 156.9, 140.9, 140.4, 135.9, 129.3, 129.1, 128.1, 127.9, 127.2, 80.6, 52.0, 43.9, 40.1, 28.4. HRMS: calculated for $C_{22}H_{27}N_2O_5S_2$ $[M+H]^+$: 463.1363, found: 463.1359.

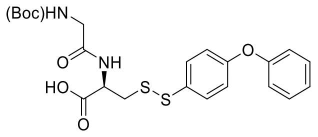

28a

N-((tert-Butoxycarbonyl)glycyl)-S-((4-phenoxyphenyl)thio)-L-cysteine

28a: Compound was prepared with a similar procedure to that employed for the synthesis of **[27a]** starting from **[S7]** and column purified (DCM – 4% MeOH/DCM/AcOH). Yield: 25 mg, 0.05 mmol, 59%. NMR: ^1H NMR (500 MHz, $CDCl_3$). δ 7.49 (d, *J* = 8.4 Hz, 2H), 7.38 – 7.31 (m, 2H), 7.28 – 7.21 (s, 1H), 7.15 – 7.10 (m, 1H), 7.04 – 6.98 (m, 2H), 6.97 – 6.91 (m, 2H), 5.46 (s, 1H), 5.08 – 4. 78 (m, 1H), 4.04 – 3.89 (m, 1H), 3.78 (dd, *J* = 17.2, 5.5 Hz, 1H), 3.37 – 3.22 (m, 1H), 3.17 (dd, *J* = 6.7, 14.2 Hz, 1H), 1.44 (s, 9H). ^{13}C NMR (126 MHz, $CDCl_3$) δ 173.4, 170.5, 158.0, 156.6, 132.2, 130.2, 130.0, 124.0, 119.5, 119.3, 81.0, 52.1, 44.2, 39.7, 28.5. HRMS: calculated for $C_{22}H_{27}N_2O_6S_2$ $[M+H]^+$: 479.1312, found: 479.1309.

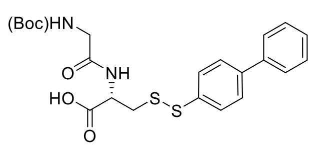

27b

S-([1,1'-Biphenyl]-4-ylthio)-N-((tert-butoxycarbonyl)glycyl)-D-cysteine

27b: Compound was prepared with a similar procedure to that employed for the synthesis of **[27a]** starting from **[S7]**. Yield: 59 mg, 0.13 mmol, 71%. NMR: ^1H NMR (500 MHz, MeOD/$CDCl_3$) δ 7.61 – 7.52 (m, 6H), 7.43 – 7.37 (m, 2H), 7.34 – 7.29 (m, 1H), 4.82 – 4.74 (m, 1H), 3.80 – 3.65 (m, 2H), 3.29 (dd, *J* = 14.0, 4.6 Hz, 1H), 3.17 – 3.09 (m, 1H), 1.42 (s, 9H). ^{13}C NMR (126 MHz, MeOD/$CDCl_3$) δ 171.5,

157.1, 141.0, 140.6, 136.0, 129.3, 129.3, 128.2, 128.0, 127.3, 80.6, 52.2, 44.3, 40.1, 28.5. HRMS: calculated for $C_{22}H_{27}N_2O_5S_2$ [M+H]$^+$: 463.1363, found: 463.1359.

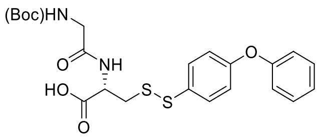

28b

_N_-((_tert_-Butoxycarbonyl)glycyl)-_S_-((4-phenoxyphenyl)thio)-D-cysteine

28b: Compound was prepared with a similar procedure to that employed for the synthesis of **[27a]** starting from **[S7]**. Yield: 46 mg, 0.1 mmol, 57%. NMR: ^{1}H NMR (500 MHz, DMSO-*d6*). δ 8.18 (d, *J* = 7.1 Hz, 1H), 7.58 – 7.50 (m, 2H), 7.44 – 7.35 (m, 2H), 7.20 – 7.14 (m, 1H), 7.07 – 6.99 (m, 4H), 6.96 (t, *J* = 6.0 Hz, 1H), 4.59 – 4.42 (m, 1H), 3.58 (d, *J* = 5.9 Hz, 2H), 3.16 (dd, *J* = 13.6, 4.8 Hz, 1H), 3.06 (dd, *J* = 13.6, 8.3 Hz, 1H), 1.37 (s, 9H). ^{13}C NMR (126 MHz, DMSO-*d6*) δ 171.7, 169.4, 156.7, 156.2, 155.8, 130.8, 130.2, 123.9, 119.3, 118.9, 78.1, 51.5, 43.0, 39.7, 28.2. HRMS: calculated for $C_{22}H_{27}N_2O_6S_2$ [M+H]$^+$: 479.1312, found: 479.1308.

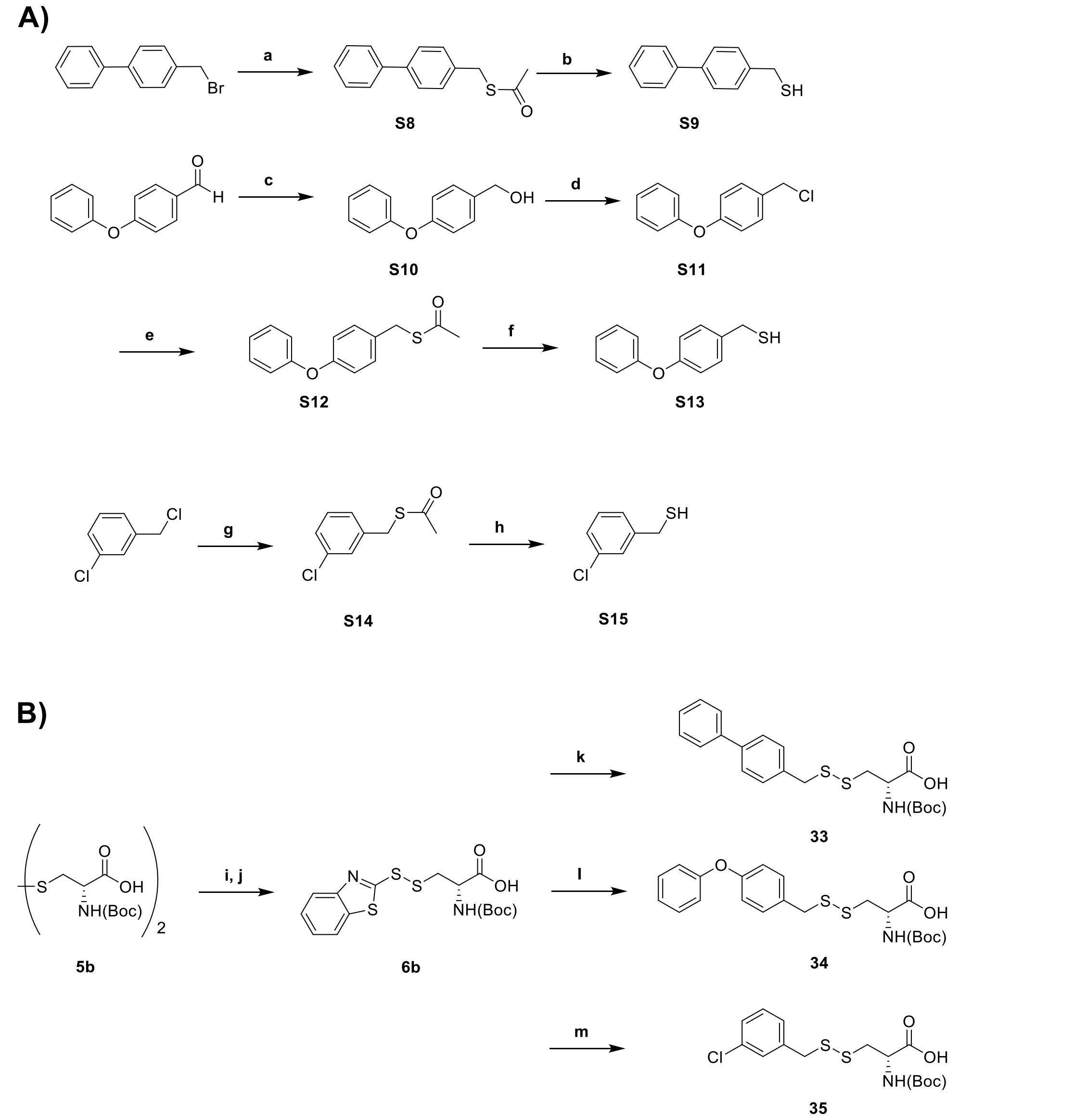

Scheme S3. *Synthesis of biaryl benzyl thiols and construction of unsymmetric disulfides, based on* D*-cysteine. A) Synthesis of thiols. B) Synthesis of unsymmetric disulfides, based on* D*-cysteine. Reagents and conditions: (a) potassium thioacetate, DMF, RT, o/n; (b) NaOH, MeOH, 0 ^{o}C – RT, 1.5 h; (c) $NaBH_4$, EtOH, 0 ^{o}C – RT, 1.5 h; (d) $SOCl_2$, $CHCl_3$, reflux, o/n; (e) potassium thioacetate, DMF, RT, 2 h; (f) NaOH, MeOH, 0 ^{o}C – RT, 0.5 h; (g) potassium thioacetate, DMF, 0 ^{o}C – RT, 0.5 h; (h) NaOH, MeOH, 0 ^{o}C – RT, 0.5 h; (i) PPh_3, H_2O, THF, 50 ^{o}C, o/n; (j) 2,2'-dithiobis(benzothiazole), $CHCl_3$, RT, 3 h; (k)* ***S9****, $CHCl_3$, RT, o/n; (l)* ***S13****, $CHCl_3$, RT, o/n; (m)* ***S15****, $CHCl_3$, RT, o/n.*

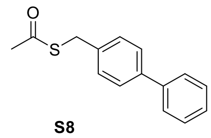

S8

***S*-([1,1'-Biphenyl]-4-ylmethyl) ethanethioate**

S8: Compound was synthesized from 4-(bromomethyl)-1,1'-biphenyl as described before [3]. Potassium thioacetate (0.83 g, 7.3 mmol) was dissolved in dry DMF (3 mL) and brought under N_2 atmosphere. 4-(Bromomethyl)-1,1'-biphenyl (1.5 g, 6.1 mmol) was dissolved in dry DMF (5 mL) and

added slowly to the potassium thioacetate. The reaction was stirred at RT overnight. Water (50 mL) was added and the mixture was extracted by Et_2O (3 x 40 mL). The combined organic layers were dried and the crude subjected to column purification to yield the title compound. Yield: 0.6 g, 6.1 mmol, 42%. NMR: ^1H NMR (500 MHz, $CDCl_3$) δ 7.59 – 7.50 (m, 4H), 7.46 – 7.40 (m, 2H), 7.39 – 7.32 (m, 3H), 4.17 (s, 2H), 2.37 (s, 3H). ^{13}C NMR (126 MHz, $CDCl_3$) δ 195.3, 140.9, 140.4, 136.8, 129.4, 128.9, 127.5, 127.5, 127.2, 33.3, 30.5.

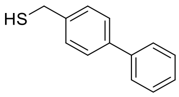

S9

[1,1'-Biphenyl]-4-ylmethanethiol

S9: S-([1,1'-biphenyl]-4-ylmethyl) ethanethioate **[S8]** (0.3g, 1.2 mmol) was dissolved in DCM (10 mL). Sample was cooled on ice and N_2 flushed. NaOH (1 M in MeOH, 6.3 mL, 6.3 mmol) was added dropwise to the sample on ice. Reaction was run at RT for 1.5 hours. Solution was acidified by HCl (5 M). Water (20 mL) was added and layers were separated. The aqueous layer was extracted with DCM (3 x 10 mL). Combined organic layers were dried and concentrated. The resulting product was used straight for disulfide synthesis.

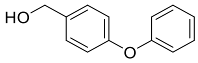

S10

(4-Phenoxyphenyl)methanol

S10: Compound was synthesized according to literature procedures [4].

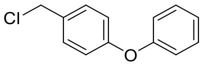

S11

1-(Chloromethyl)-4-phenoxybenzene

S11: Compound was synthesized according to literature procedures [5].

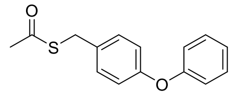

S12

S-(4-phenoxybenzyl) ethanethioate

S12: Potassium thioacetate (0.46 g, 4.0 mmol) was dissolved in dry DMF (2 mL) and brought under N_2 atmosphere. 1-(Chloromethyl)-4-phenoxybenzene **[S11]** (0.7 g, 3.3 mmol) was dissolved in dry DMF (3 mL) and added slowly to the potassium thioacetate. The reaction was stirred at RT for 2 h. Water (25 mL) was added and the mixture was extracted with Et_2O (3 x 20 mL). The combined organic layers were dried and the resulting crude was subjected to column purification (30% DCM/PE) to yield the title compound. Yield: 0.56 g, 2.2 mmol, 65%. NMR: ^{1}H NMR (500 MHz, $CDCl_3$) δ 7.36 – 7.31 (m, 2H), 7.27 – 7.23 (m, 2H), 7.14 – 7.07 (m, 1H), 7.04 – 6.97 (m, 2H), 6.97 – 6.90 (m, 2H), 4.11 (s, 2H), 2.36 (s, 3H). ^{13}C NMR (126 MHz, $CDCl_3$) δ 195.3, 157.2, 156.6, 132.5, 130.3, 129.9, 123.5, 119.1, 119.0, 33.0, 30.5.

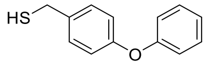

S13

(4-Phenoxyphenyl)methanethiol

S13: Compound was prepared with a similar procedure to that employed for the synthesis of **[S9]** starting from **[S12]**.

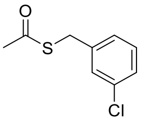

S14

S-(3-Chlorobenzyl) ethanethioate

S14: 1-Chloro-3-chloromethyl benzene (3.0 g, 18.6 mmol) was mixed with DMF (105 mL). The solution was cooled to 0 ˚C and potassium thioacetate (6.4 g , 56 mmol) was added. The reaction was stirred at RT for 0.5 h, diluted with DCM (150 mL) and washed with water (3 x 150 mL). The organic

layer was dried, concentrated and column purified (3% EtOAc/PE) to yield the title compound. Yield: 3.0 g, 15 mmol, 80%. NMR: ^{1}H NMR (400 MHz, $CDCl_3$) δ 7.32 – 7.27 (m, 1H), 7.24 – 7.13 (m, 3H), 4.09 (s, 2H), 2.37 (s, 3H).

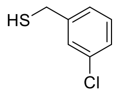

S15

(3-Chlorophenyl)methanethiol

S15: S-(3-Chlorobenzyl) ethanethioate **[S14]** (0.5 g, 2.5 mmol) was dissolved in EtOH (9 mL). The solution was cooled to 0 ˚C and brought under N_2 atmosphere. NaOH (5 M in MeOH, 6 mL) was added slowly and the reaction stirred for 30 min. at 0 ˚C. The mixture was neutralized with the addition of HCl (1 M) followed by extraction with DCM (3 x 90 mL). The combined organic layers were washed by brine, dried and concentrated. The title compound was used without further purification.

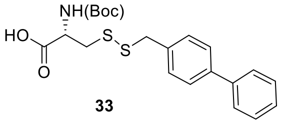

33

***S*-(([1,1'-Biphenyl]-4-ylmethyl)thio)-*N*-(*tert*-butoxycarbonyl)-D-cysteine**

33: *S*-(Benzo[*d*]thiazol-2-ylthio)-*N*-(*tert*-butoxycarbonyl-D-cysteine **[6b]** (0.2 g, 0.52 mmol) was dissolved in $CHCl_3$ (16 mL) and brought under N_2 atmosphere. **S9** (0.21 g, 1.0 mmol) was dissolved in $CHCl_3$ (2 mL) and added dropwise. The reaction was stirred at RT overnight. Once complete, the mixture was concentrated and column purified (DCM – 5% MeOH/DCM/0.5% AcOH) to yield the title compound. Yield: 0.17 g, 0.40 mmol, 78%. NMR: ^{1}H NMR (400 MHz, DMSO-*d6*) δ 7.68 – 7.59 (m, 4H), 7.50 – 7.39 (m, 4H), 7.39 – 7.33 (m, 1H), 7.24 (d, *J* = 8.4 Hz, 1H), 4.20 (ddd, *J* = 10.2, 8.4, 4.1 Hz, 1H), 4.07 – 3.95 (m, 2H), 2.95 (dd, *J* = 13.6, 4.1 Hz, 1H), 2.78 (dd, *J* = 13.6, 10.2 Hz, 1H), 1.38 (s, 9H). ^{13}C NMR (101 MHz, DMSO-*d6*) δ 172.6, 155.5, 139.9, 139.2, 136.7, 129.9, 129.0, 127.5, 126.8, 126.7, 78.4, 52.7, 41.6, 39.1, 28.2. HRMS: calculated for $C_{21}H_{26}NO_4S_2$ $[M+H]^+$: 420.1305, found: 420.1299.

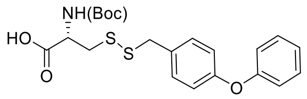

34

N-(tert-Butoxycarbonyl)-S-((4-phenoxybenzyl)thio)-D-cysteine

34: Compound was prepared with a similar procedure to that employed for the synthesis of **[33]** starting from **[S13]**. Yield: 0.11 g, 0.25 mmol, 65%. NMR: ^{1}H NMR (400 MHz, $CDCl_3$) δ 8.01 (s, 1H), 7.38 – 7.31 (m, 2H), 7.31 – 7.23 (m, 2H), 7.15 – 7.06 (m, 1H), 7.04 – 6.91 (m, 4H), 5.33 (d, *J* = 8.0 Hz, 1H), 4.55 (q, *J* = 6.3 Hz, 1H), 3.89 (s, 2H), 2.99 – 2.68 (m, 2H), 1.45 (s, 9H). ^{13}C NMR (101 MHz, $CDCl_3$) δ 175.5, 157.1, 157.0, 155.5, 131.7, 130.9, 129.9, 123.5, 119.1, 119.0, 80.8, 52.9, 42.9, 40.1, 28.4. HRMS: calculated for $C_{21}H_{26}NO_5S_2$ $[M+H]^+$: 436.1254, found: 436.1245.

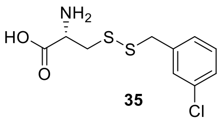

35

N-(tert-Butoxycarbonyl)-S-((3-chlorobenzyl)thio)-D-cysteine

35: Compound was prepared with a similar procedure to that employed for the synthesis of **[33]** starting from **[S15]**. Yield: 0.52 g, 1.37 mmol, 90%. ^{1}H NMR (400 MHz, $CDCl_3$) δ 8.00 (s, 1H), 7.33 – 7.30 (m, 1H), 7.28 – 7.24 (m, 2H), 7.23 – 7.17 (m, 1H), 5.32 (d, *J* = 7.9 Hz, 1H) 4.60 – 4.36 (m, 1H), 3.86 (s, 2H), 2.99 – 2.71 (m, 2H), 1.46 (s, 9H). ^{13}C NMR (101 MHz, $CDCl_3$) δ 175.3, 155.5, 139.1, 134.5, 130.0, 129.5, 127.9, 127.7, 80.9, 52.9, 42.9, 40.2, 28.4. HRMS: calculated for $C_{21}H_{26}NO_5S_2$ $[M(-Boc)+H]^+$: 278.0078, found: 278.0078.

Synthetic procedures and analytical data for unnatural amino acid 44 and its precursors

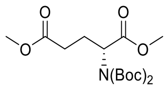

39

Dimethyl (R)-2-Di-tert-butoxycarbonyl-aminopentanedioate

39: Compound was synthesized as described before [6]. Dimethyl D-glutamate (HCl salt, 2.1 g, 10 mmol) was dissolved in MeOH (26 mL). Et_3N (9.1 mL, 65 mmol) was added, followed by the addition of di-*tert*-butyl dicarbonate (5.5 g, 25 mmol). The mixture was stirred at RT overnight and concentrated. DCM (30 mL) was added and subsquently washed with aqueous citric acid (1 M, 3 x 20 mL) and water (20 mL). The organic layer was dried and evaporated completely. The resulting solid, together with DMAP (1.8g, 14.6 mmol), was dissolved in ACN (30 mL). Di-*tert*-butyl dicarbonate (2.8g, 13.0 mmol) was added and reaction was stirred at RT overnight. Once complete, the mixture was concentrated. The residue was column purified (10% EtOAc/PE) to yield the title compound. Yield: 2.0 g, 5.4 mmol, 54%. The analytical data (NMR) was consistent with literature ([6]).

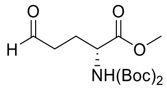

40

Methyl (R)-2-Di-tert-butoxycarbonylamino-5-oxopentanoate

40: Compound was synthesized as described before [6], starting from [**39**]. Yield: 1.3 g, 3.9 mmol, 72%. The analytical data (NMR) was consistent with literature ([6]).

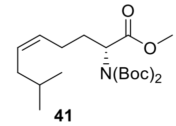

41

Methyl (5Z,2R)-2-Di-tert-butoxycarbonylamino-8-methylnon- 5-enoate

41: Isopentyltriphenylphosphonium bromide (1.9 g, 4.5 mmol) was suspended in dry toluene, brought under N_2 atmosphere, cooled to 0 °C and $KN(TMS)_2$ (0.5 M in toluene, 8.3 mL, 4.1 mmol) was added dropwise. After 15 min., the flask was cooled to -78 °C. Methyl (R)-2-Di-tert-

butoxycarbonylamino-5-oxopentanoate **[40]** (1.3 g, 3.7 mmol) was dissolved in dry toluene (4 mL) and added dropwise to the mixture. The reaction was stirred for 2 h at -78 ^{o}C. Once complete, the mixture was quenched with aqueous NH_4Cl (saturated, 32 mL) and extracted with Et_2O (3 x 20 mL). The combined organics were washed with brine (30 mL), dried and concentrated. The crude was column purified (20% Et_2O/PE) to yield the title compound as a transparent oil. Yield: 0.85 g, 2.1 mmol, 57%. NMR: ^{1}H NMR (400 MHz, $CDCl_3$) δ 5.43 – 5.33 (m, 2H), 4.84 (dd, *J* = 9.0, 4.8 Hz, 1H), 3.68 (s, 3H), 2.20 – 1.98 (m, 3H), 1.93 – 1.82 (m, 3H), 1.56 (n, *J* = 6.7 Hz, 1H), 1.47 (s, 18H), 0.85 (dd, *J* = 6.7, 2.7 Hz, 6H). ^{13}C NMR (101 MHz, $CDCl_3$) δ 171.4, 152.1, 129.9, 128.9, 83.1, 57.8, 52.2, 36.4, 30.2, 28.7, 28.1, 24.2, 22.5, 22.4.

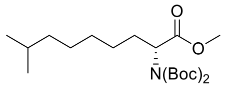

42

Methyl (R)-2-Di-tert-butoxycarbonylamino-8-methylnonate

42: Methyl (5Z,2R)-2-Di-tert-butoxycarbonylamino-8-methylnon- 5-enoate **[41]** (0.8 g, 2.0 mmol) was dissolved in EtOH (4 mL). Pd/C (10% w/w, 0.15 g, 0.14 mmol) was suspended in EtOH (4mL) and was added to the above solution. The mixture was brought under N_2 atmosphere. Triethylsilane (3.2 mL, 20 mmol) was added dropwise to the mixture. The reaction was run at RT for 40 min. and the resulting mixture filtered over Celite. The filtrate was concentrated and column purified (10% Et_2O/PE) to yield the title compound. Yield: 0.72 g, 1.8 mmol, 90%. NMR: ^{1}H NMR (400 MHz, $CDCl_3$) δ 4.86 (dd, *J* = 9.7, 5.0 Hz, 1H), 3.71 (s, 3H), 2.15 – 2.02 (m, 1H), 1.94 – 1.82 (m, 1H), 1.47 (s, 18H), 1.40 – 1.22 (m, 6H), 1.19 – 1.10 (m, 2H), 0.85 (d, *J* = 6.6 Hz, 6H). ^{13}C NMR (101 MHz, $CDCl_3$) δ 171.6, 152.2, 83.0, 58.2, 52.2, 39.0, 29.9, 29.6, 28.1, 28.0, 27.3, 26.3, 22.7, 22.7. HRMS: calculated for $C_{16}H_{32}NO_4$ $[M+H]^+$: 302.2333, found: 302.2328.

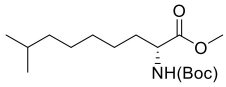

43

Methyl (*R*)-2-((*tert*-butoxycarbonyl)amino)-8-methylnonanoate

43: 8-Methyl-2-*N*-diBoc-methylnonanoic methyl ester **[42]** (0.35 g, 0.87 mmol) was dissolved in dry THF (15 mL). HCl (4 M in 1,4-dioxane, 12 mL) was added and the reaction was stirred at RT and under N_2 atmosphere for 4 h. Once complete, the reaction mixture was concentrated. The crude was dissolved in MeOH (17 mL) and Et_3N (2.4 mL, 11.5 mmol) and di-*tert*-butyl dicarbonate (0.29 g, 1.3 mmol) were added. The reaction was stirred at RT overnight. Once complete, the sample was concentrated and column purified (20% Et_2O/PE) to yield the title compound. Yield: 0.25 g, 0.81 mmol, 93%. NMR: ^{1}H NMR (400 MHz, $CDCl_3$) δ 4.99 (d, *J* = 8.5 Hz, 1H), 4.33 – 4.24 (m, 1H), 3.74 (s, 3H), 1.85 – 1.71 (m, 1H), 1.66 – 1.55 (m, 1H), 1.54 – 1.46 (m, 1H), 1.45 (s, 9H), 1.40 – 1.21 (m, 6H), 1.19 – 1.09 (m, 2H), 0.85 (d, *J* = 6.6 Hz, 6H). ^{13}C NMR (101 MHz, $CDCl_3$) δ 173.7, 155.5, 80.0, 53.6, 52.3, 39.0, 32.9, 29.6, 28.5, 28.0, 27.3, 25.4, 22.6. HRMS: calculated for $C_{16}H_{32}NO_4$ $[M+H]^+$: 302.2333, found: 302.2328.

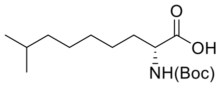

44

(*R*)-2-((*tert*-Butoxycarbonyl)amino)-8-methylnonanoic acid

44: Methyl (*R*)-2-((*tert*-butoxycarbonyl)amino)-8-methylnonanoate **[43]** (0.15 g, 0.5 mmol) was dissolved in 1,4-dioxane (8 mL). Aqueous NaOH (1 M, 50 mL) was added and reaction was stirred at RT for 2 h. The mixture was cooled at 0 ˚C and treated with aqueous HCl (5 M) to pH 2. The mixture was extracted with EtOAc (3 x 30 mL) and the combined organics were washed with brine (30 mL) and dried. The residue was concentrated and column purified (5% MeOH/DCM) to yield the title compound. Yield: 0.13 g, 0.44 mmol, 87%. NMR: ^{1}H NMR (400 MHz, $CDCl_3$) δ 8.20 (s, 1H), 5.02 (d, *J* = 8.3 Hz, 1H), 4.35 – 4.06 (m, 1H), 1.90 – 1.76 (m, 1H), 1.72 – 1.58 (m, 1H), 1.50 (n, *J* = 6.5 Hz, 1H), 1.44 (s, 9H), 1.42 – 1.32 (m, 2H), 1.31 – 1.22 (m, 4H), 1.19 – 1.09 (m, 2H), 0.85 (d, *J* = 6.6 Hz, 6H). ^{13}C NMR (101 MHz, $CDCl_3$) δ 177.9, 155.8, 80.3, 53.6, 39.0, 32.6, 29.6, 28.4, 28.0, 27.3, 25.4, 22.7. HRMS: calculated for $C_{15}H_{29}NO_4$ $[M+H]^+$: 288.2177, found: 288.2172.

HRMS data and HPLC traces of final peptide compounds

17a

Composition	Exact mass	M + H	(M+2H)/2	Found value
$C_{52}H_{91}N_{15}O_{12}S_2$	1181.6413	1182.6493	591.8287	1182.6481

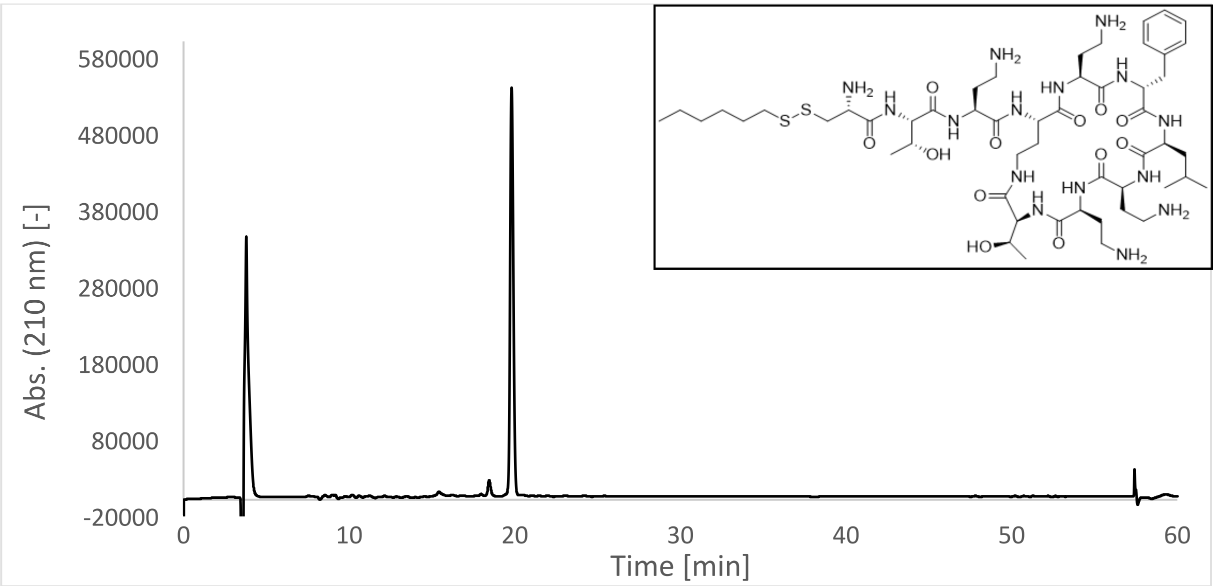

17b

Composition	Exact mass	M + H	(M+2H)/2	Found value
$C_{52}H_{91}N_{15}O_{12}S_2$	1181.6413	1182.6493	591.8287	1182.6503

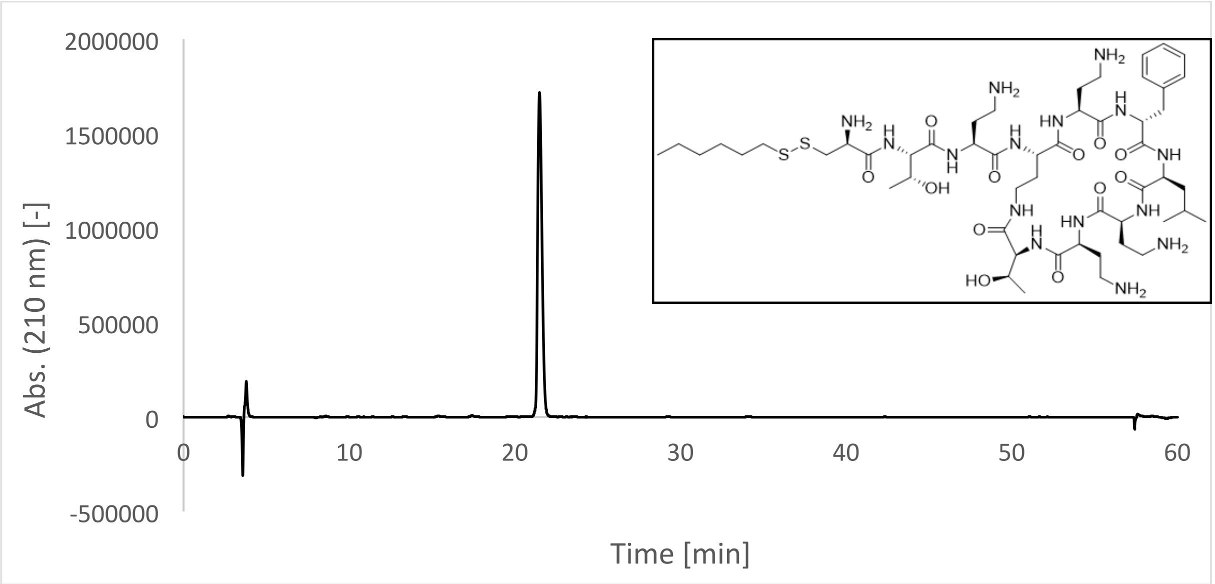

18a

Composition	Exact mass	M + H	(M+2H)/2	Found value
$C_{51}H_{89}N_{15}O_{12}S_2$	1167.6257	1168.6337	584.8209	1168.6348

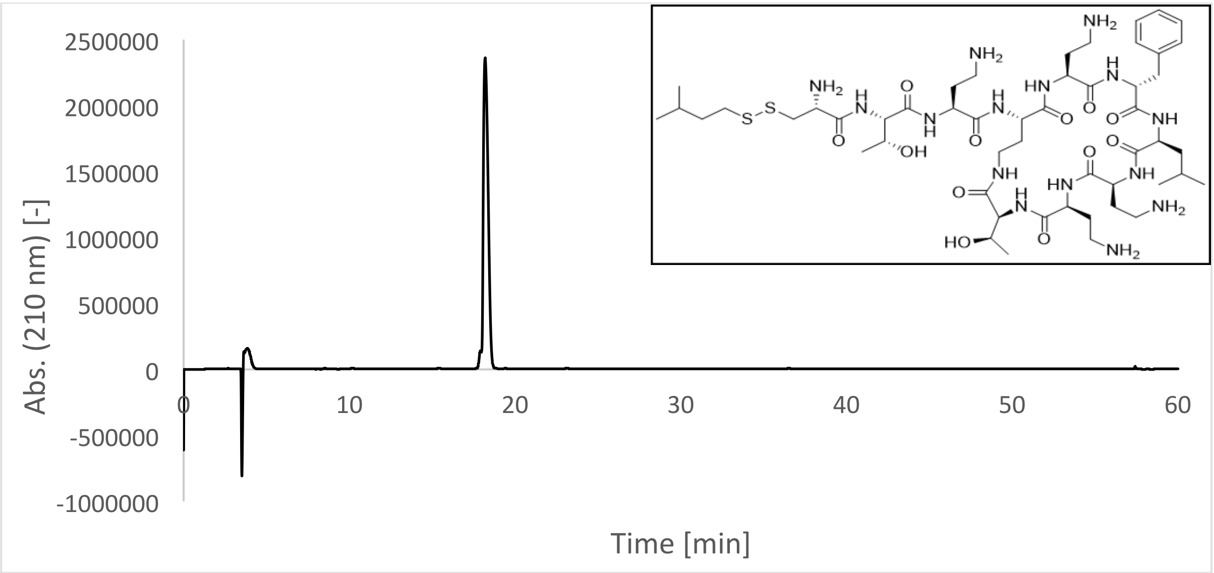

18b

Composition	Exact mass	M + H	(M+2H)/2	Found value
$C_{51}H_{89}N_{15}O_{12}S_2$	1167.6257	1168.6337	584.8209	1168.6338

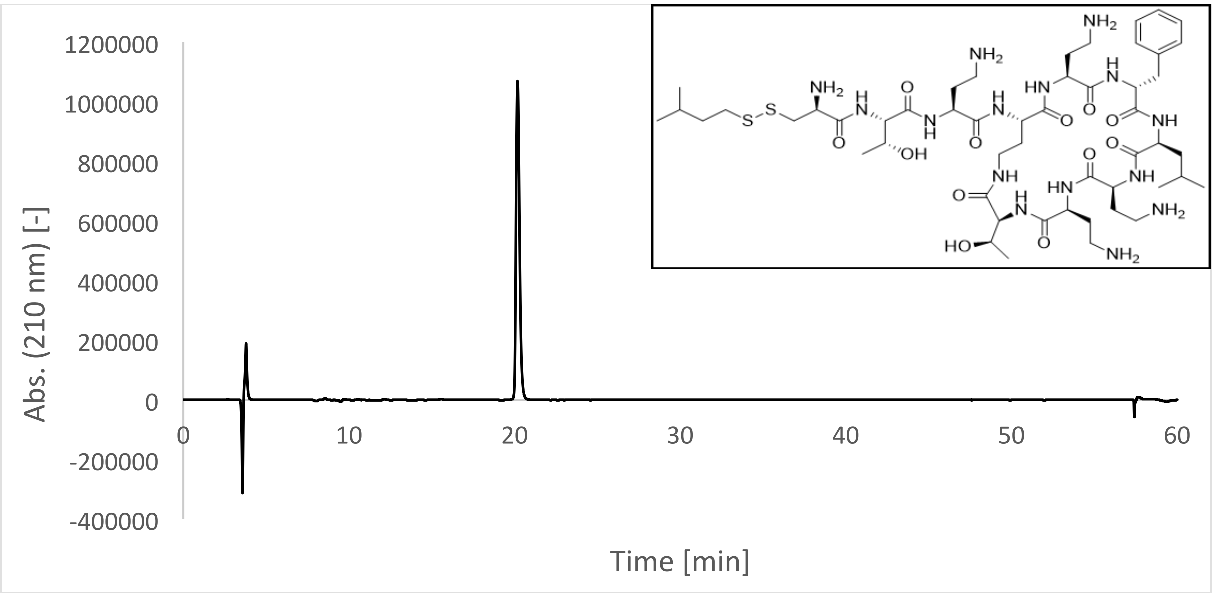

19a

Composition	Exact mass	M + H	(M+2H)/2	Found value
$C_{54}H_{93}N_{15}O_{12}S_2$	1207.6570	1208.6650	604.8365	1208.6674

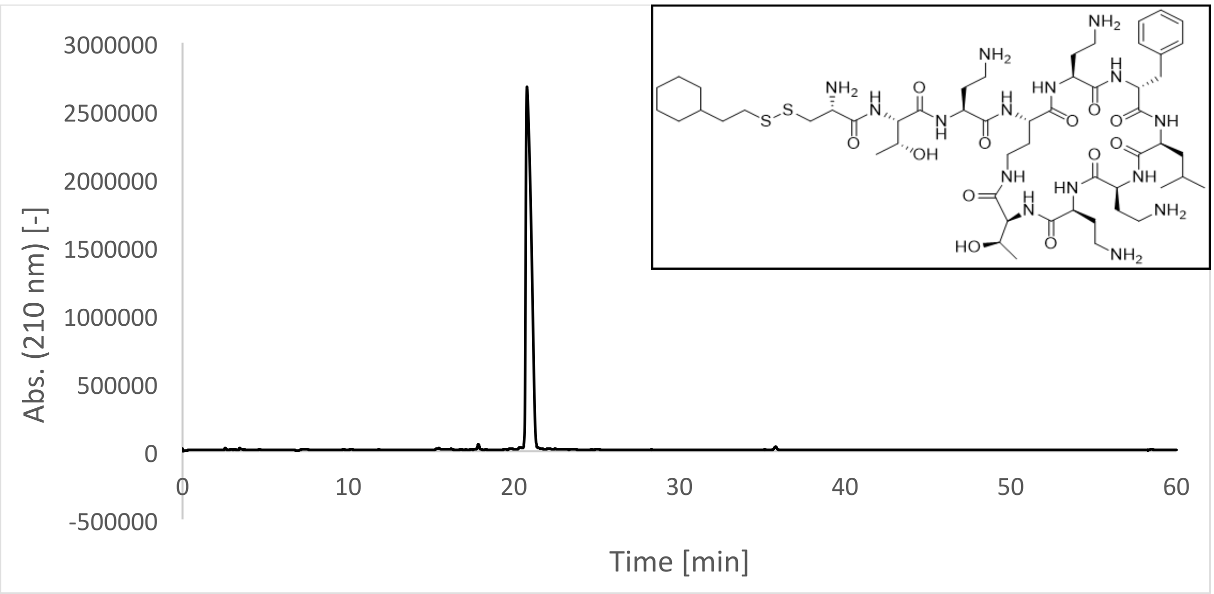

19b

Composition	Exact mass	M + H	(M+2H)/2	Found value
$C_{54}H_{93}N_{15}O_{12}S_2$	1207.6570	1208.6650	604.8365	1208.6647

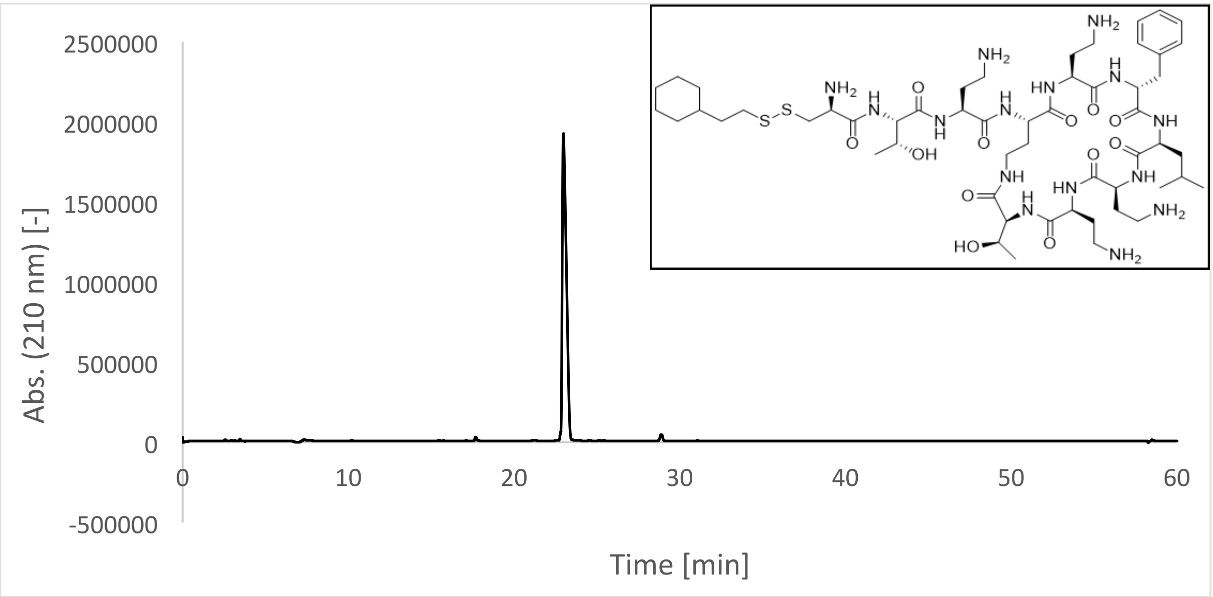

20a

Composition	Exact mass	M + H	(M+2H)/2	Found value
$C_{53}H_{93}N_{15}O_{12}S_2$	1195.6570	1196.6650	598.8365	1196.6663

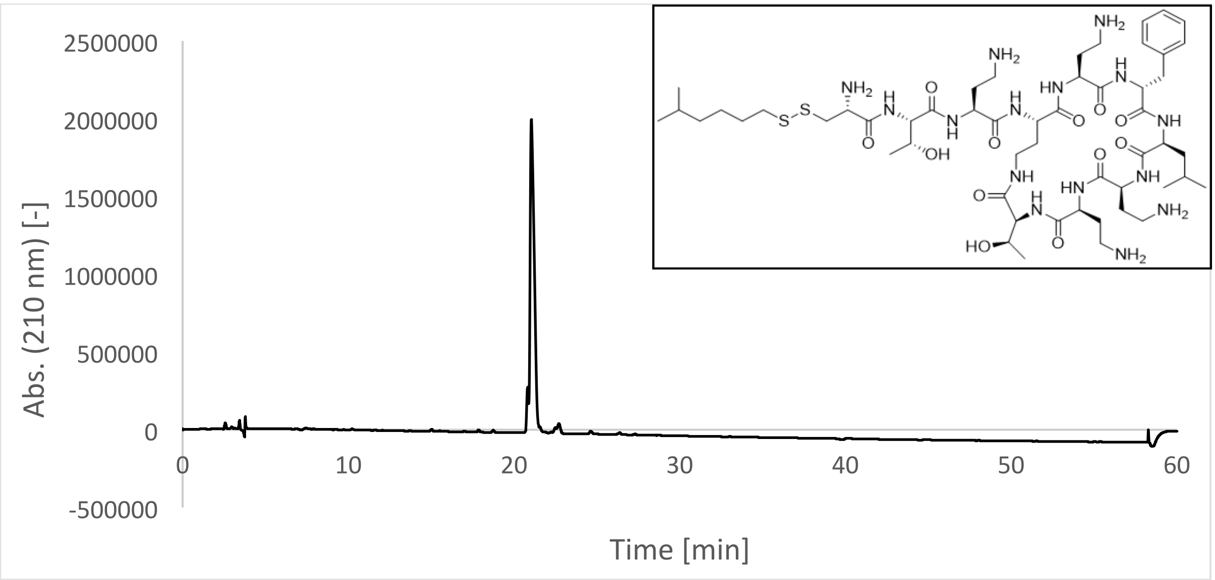

20b

Composition	Exact mass	M + H	(M+2H)/2	Found value
$C_{53}H_{93}N_{15}O_{12}S_2$	1195.6570	1196.6650	598.8365	598.8360

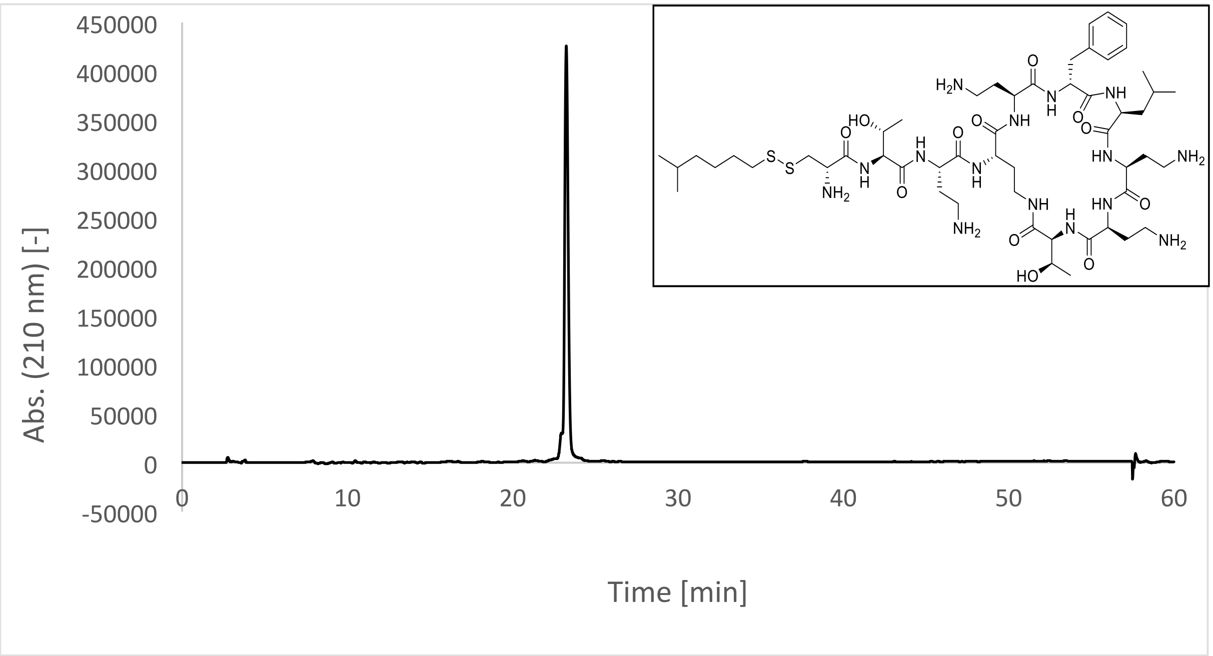

21a

Composition	Exact mass	M + H	(M+2H)/2	Found value
$C_{54}H_{94}N_{16}O_{13}S_2$	1238.6628	1239.6708	620.3394	620.3392

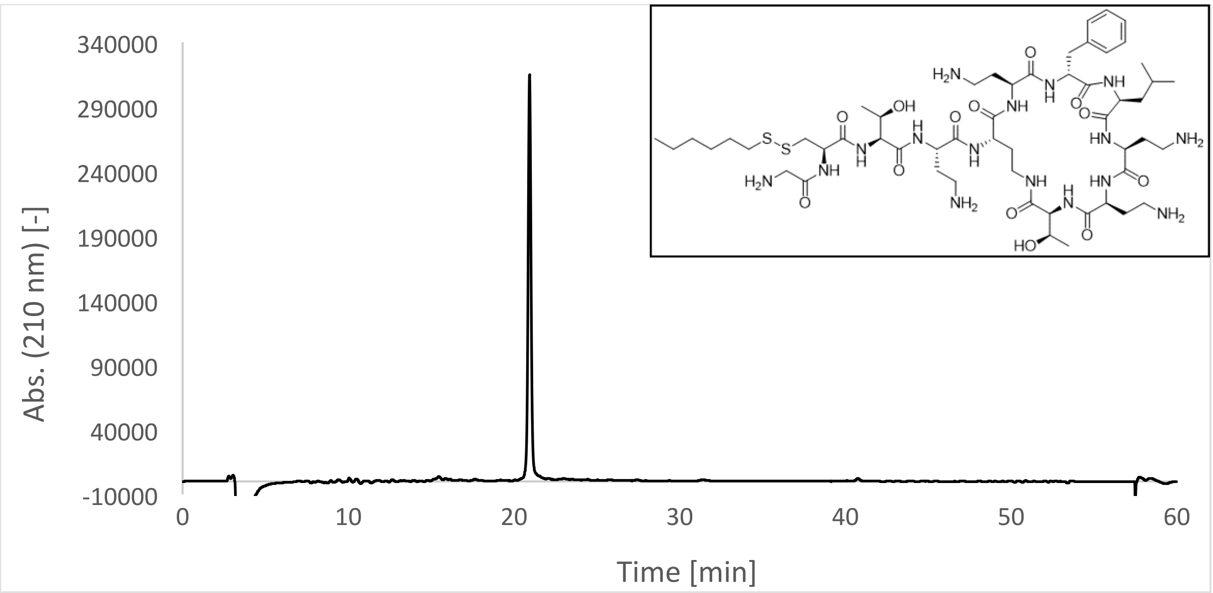

21b

Composition	Exact mass	M + H	(M+2H)/2	Found value
$C_{54}H_{94}N_{16}O_{13}S_2$	1238.6628	1239.6708	620.3394	1239.6708

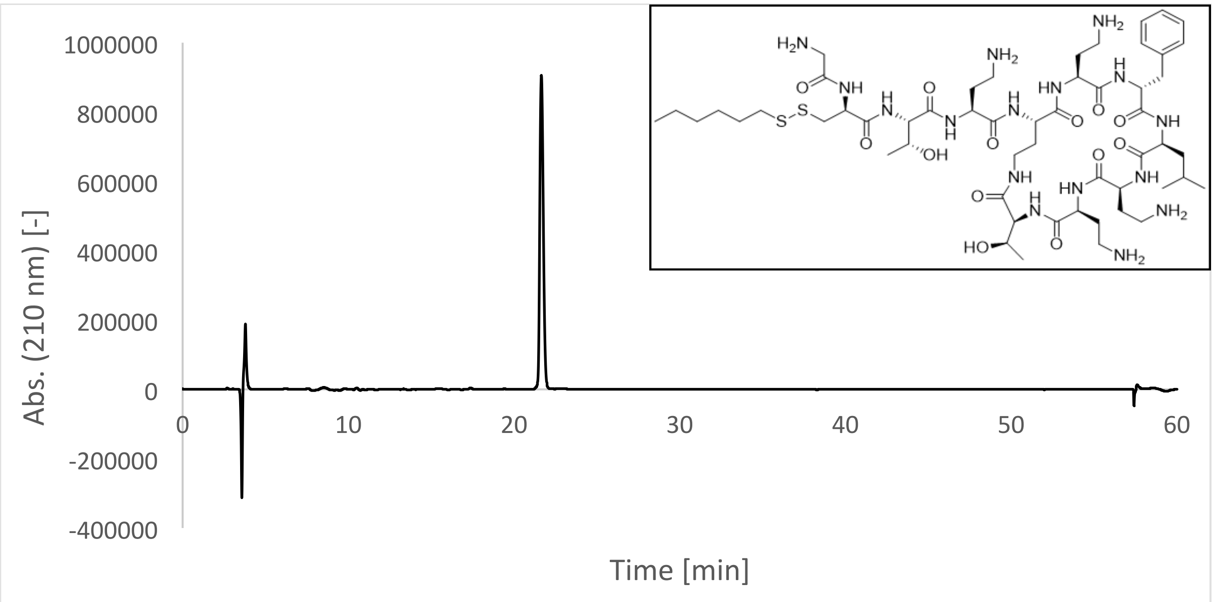

22a

Composition	Exact mass	M + H	(M+2H)/2	Found value
$C_{53}H_{92}N_{16}O_{13}S_2$	1224.6471	1225.6551	613.3316	1225.6545

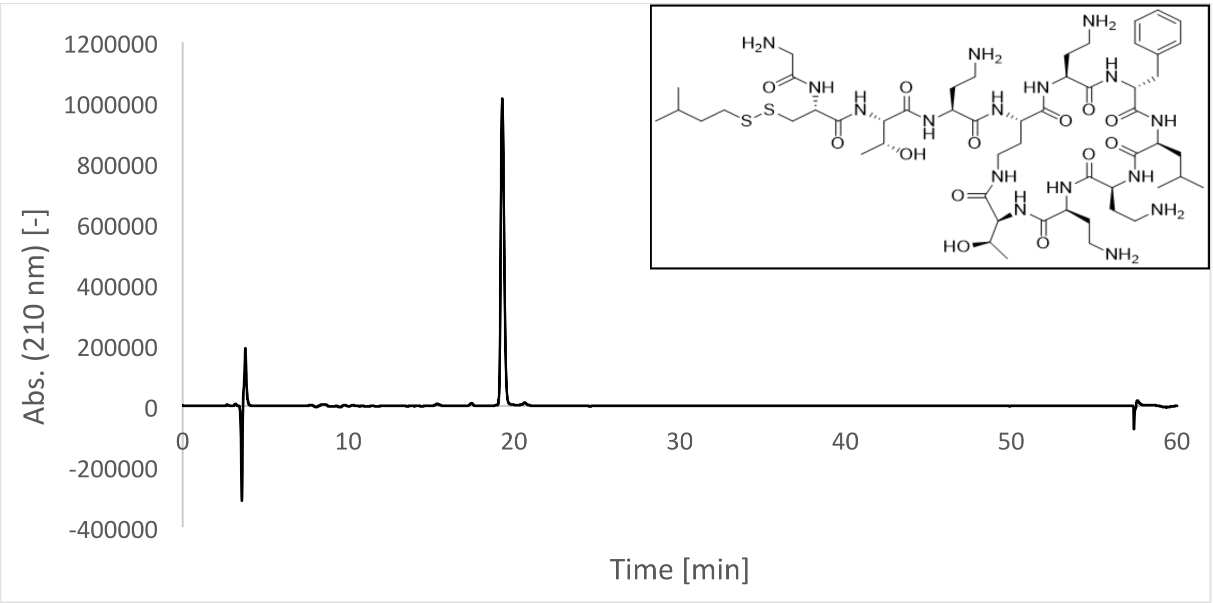

22b

Composition	Exact mass	M + H	(M+2H)/2	Found value
$C_{53}H_{92}N_{16}O_{13}S_2$	1224.6471	1225.6551	613.3316	1225.6559

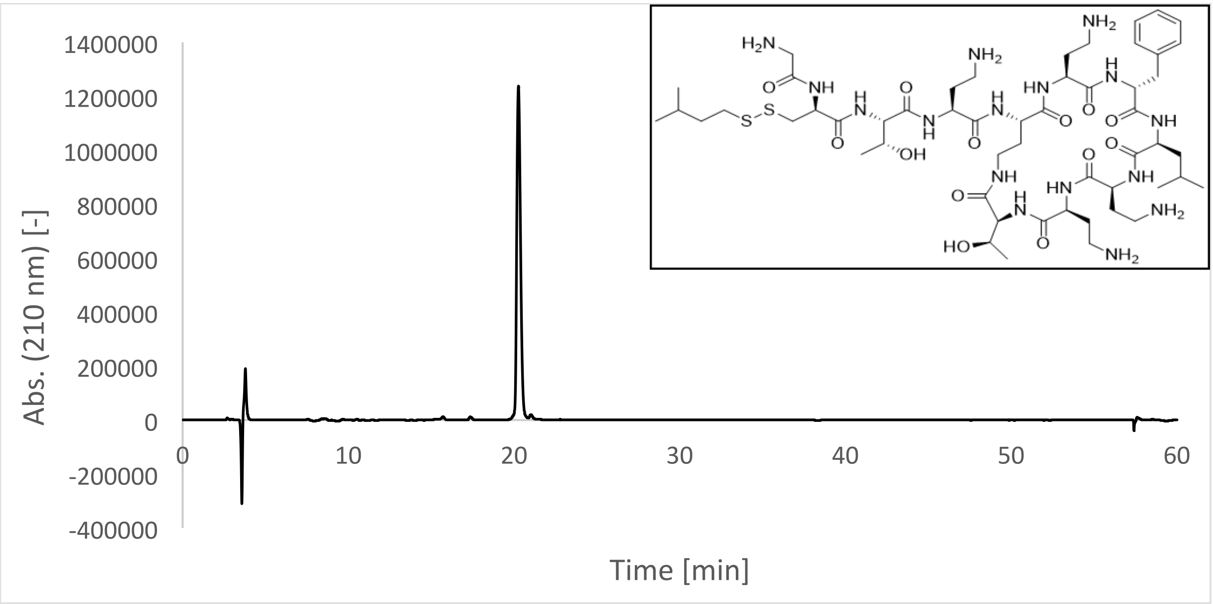

23a

Composition	Exact mass	M + H	(M+2H)/2	Found value
$C_{56}H_{96}N_{16}O_{13}S_2$	1264.6784	1265.6864	633.3472	1265.6876

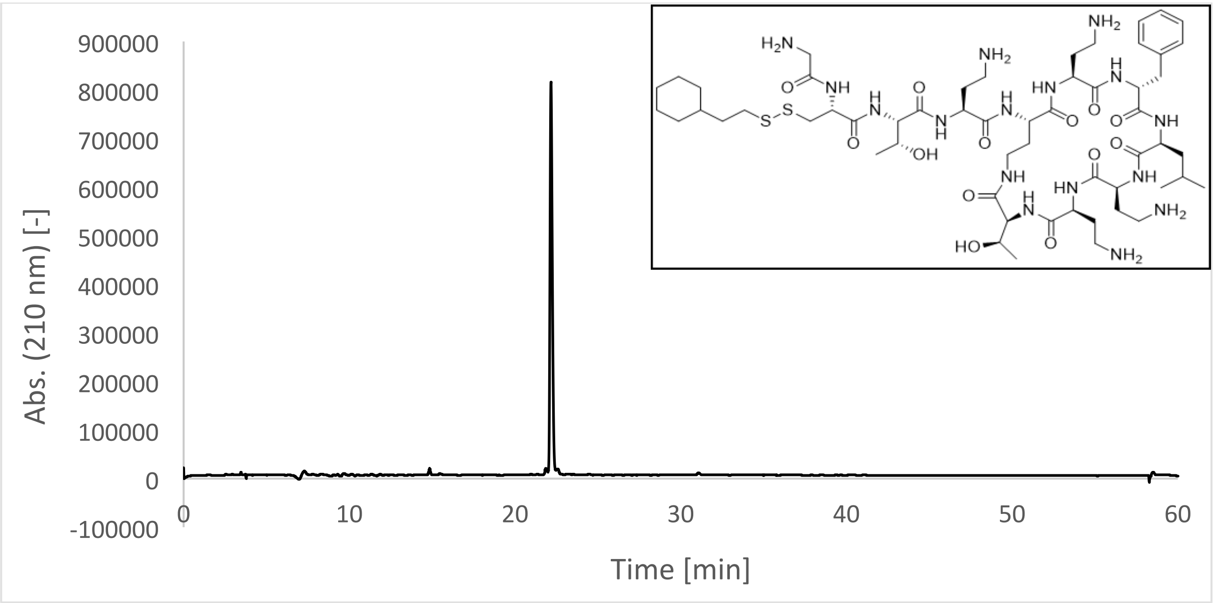

23b

Composition	Exact mass	M + H	(M+2H)/2	Found value
$C_{56}H_{96}N_{16}O_{13}S_2$	1264.6784	1265.6864	633.3472	1265.6881

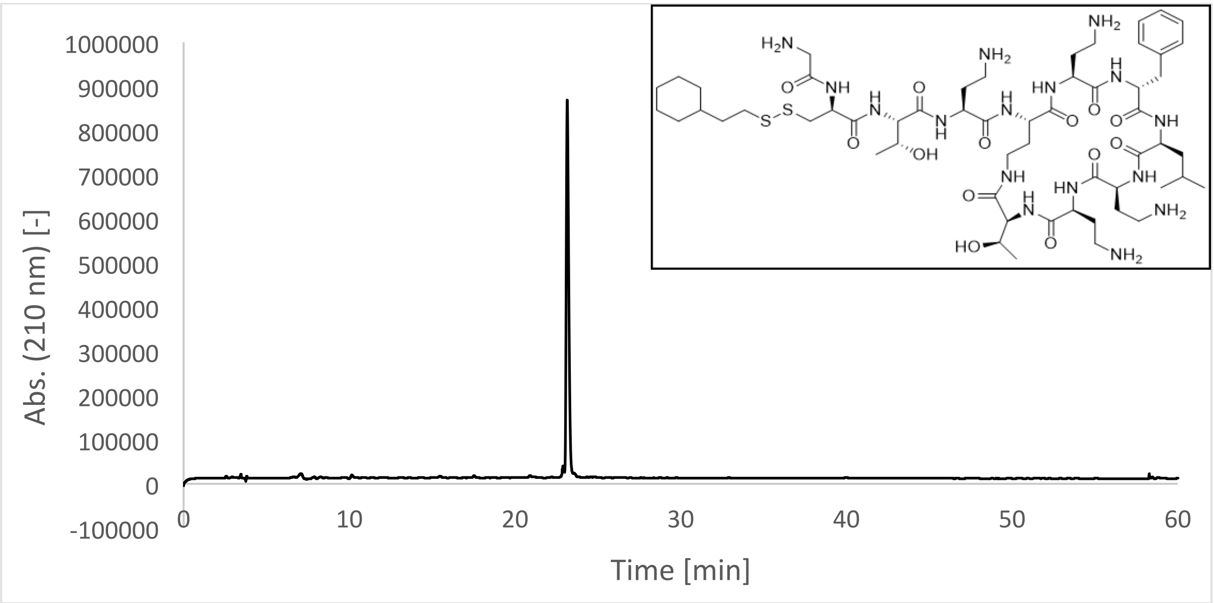

24a

Composition	Exact mass	M + H	(M+2H)/2	Found value
$C_{55}H_{96}N_{16}O_{13}S_2$	1252.6784	1253.6864	627.3472	1253.6886

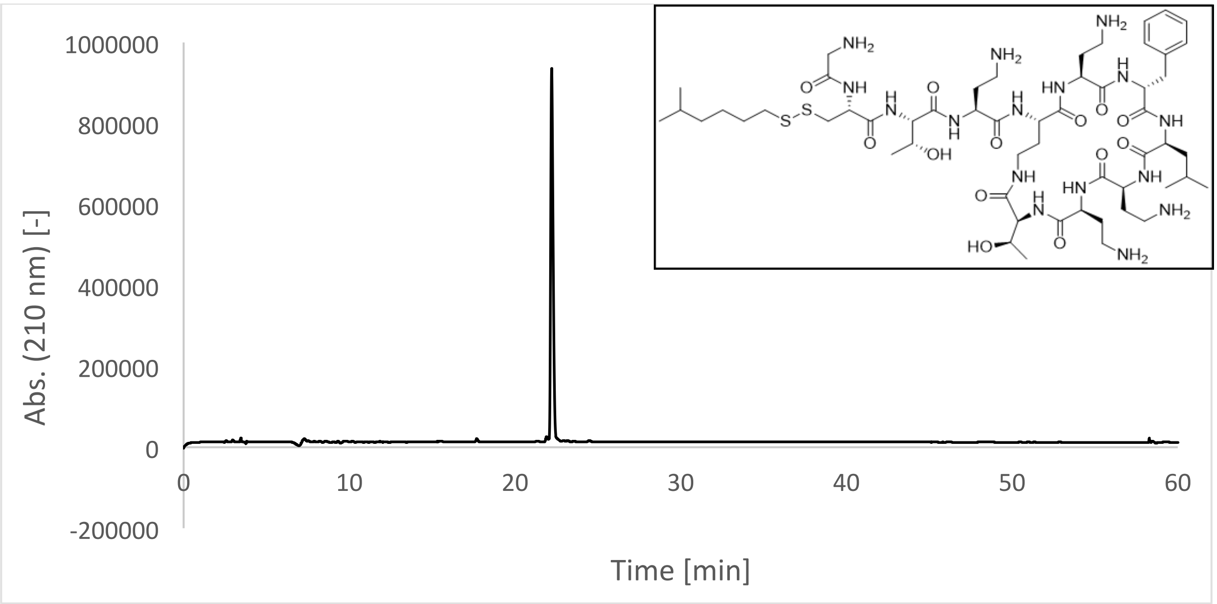

24b

Composition	Exact mass	M + H	(M+2H)/2	Found value
$C_{55}H_{96}N_{16}O_{13}S_2$	1252.6784	1253.6864	627.3472	627.3465

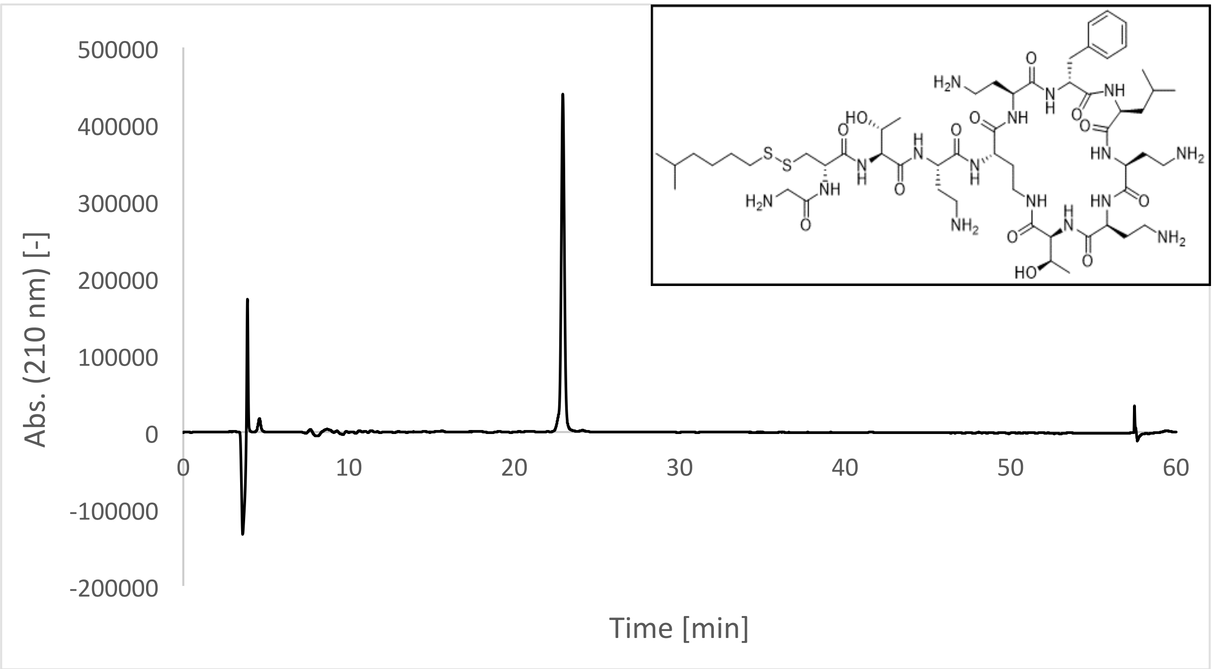

29a

Composition	Exact mass	M + H	(M+2H)/2	Found value
$C_{58}H_{87}N_{15}O_{12}S_2$	1249.6100	1250.6180	625.8130	1250.6207

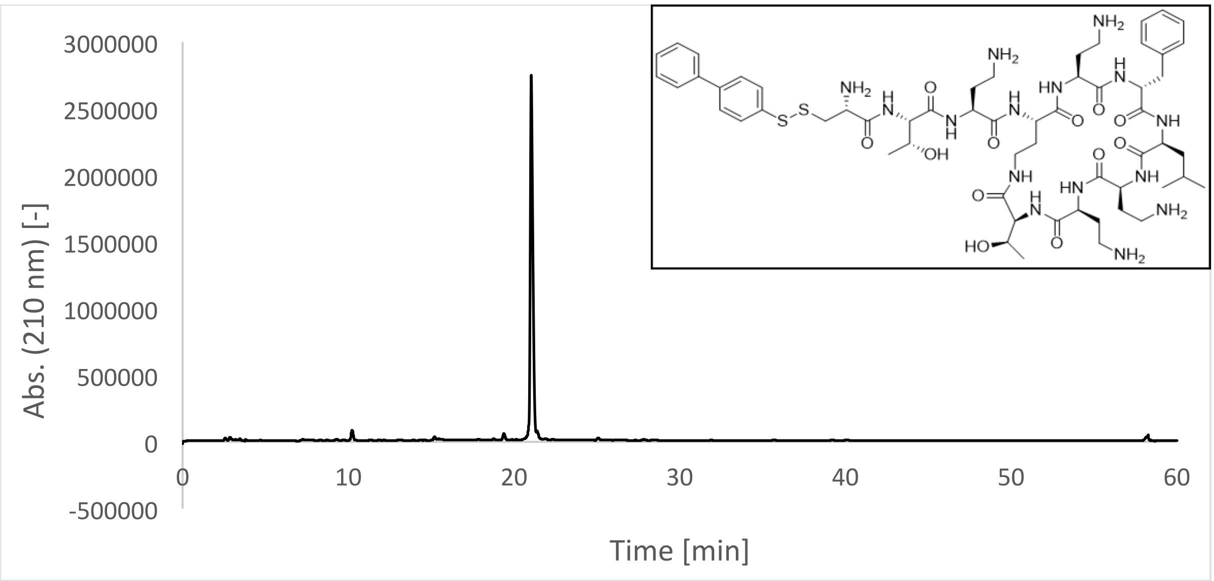

29b

Composition	Exact mass	M + H	(M+2H)/2	Found value
$C_{58}H_{87}N_{15}O_{12}S_2$	1249.6100	1250.6180	625.8130	625.8118

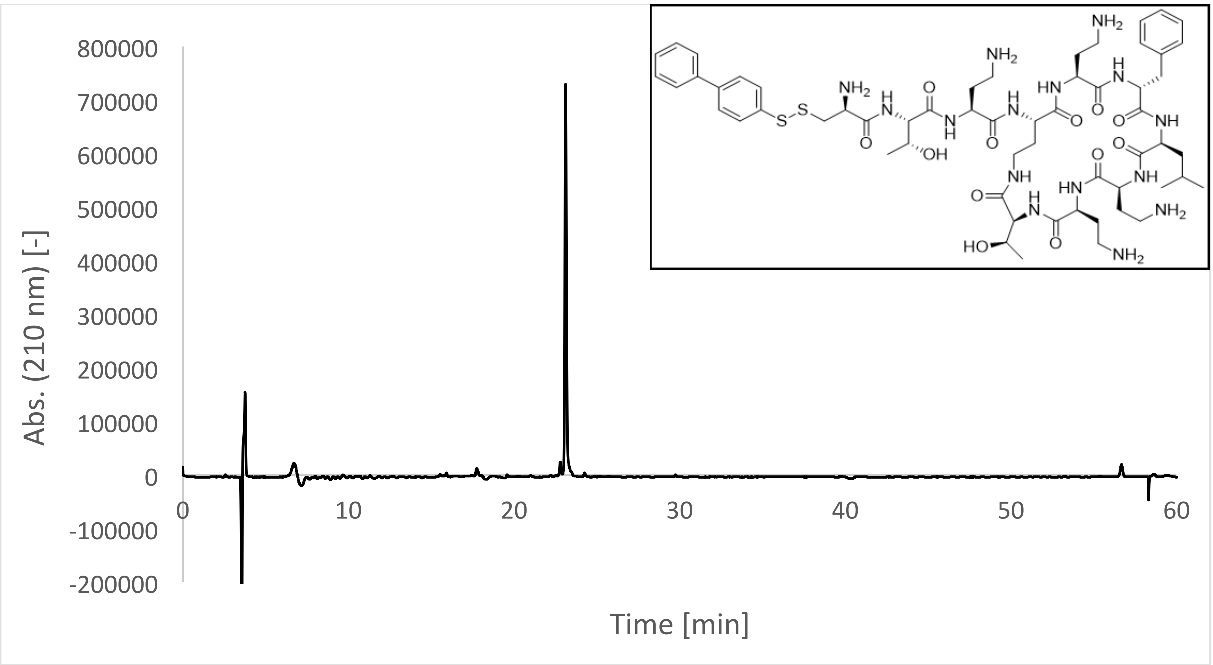

30a

Composition	Exact mass	M + H	(M+2H)/2	Found value
$C_{58}H_{87}N_{15}O_{13}S_2$	1265.6049	1266.6129	633.8105	1266.6145

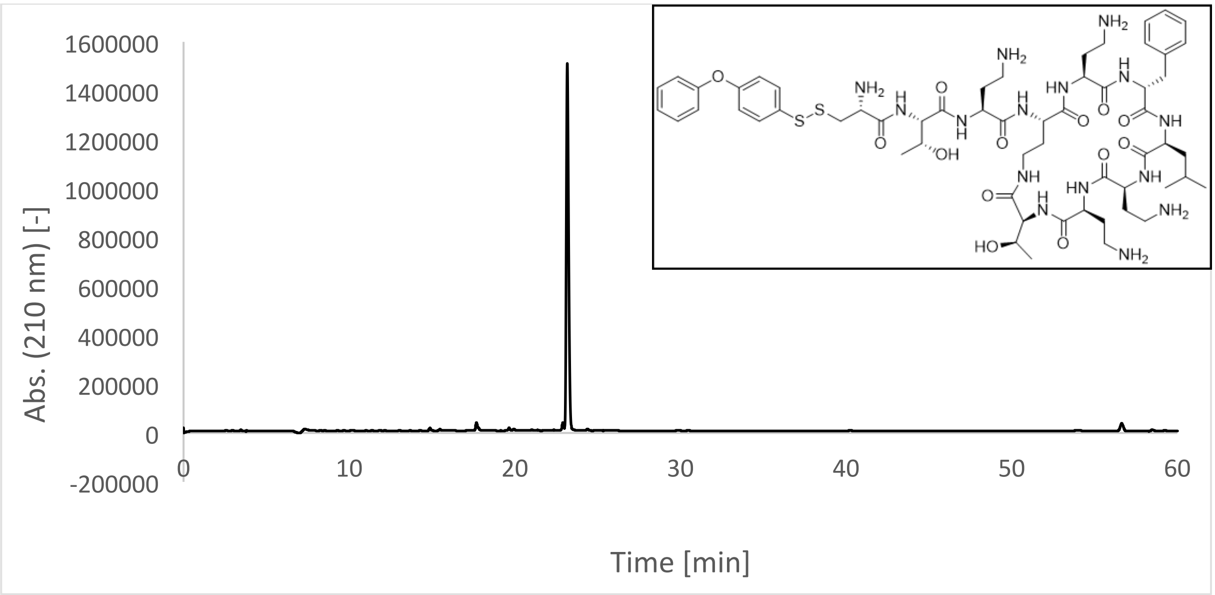

30b

Composition	Exact mass	M + H	(M+2H)/2	Found value
$C_{58}H_{87}N_{15}O_{13}S_2$	1265.6049	1266.6129	633.8105	633.8099

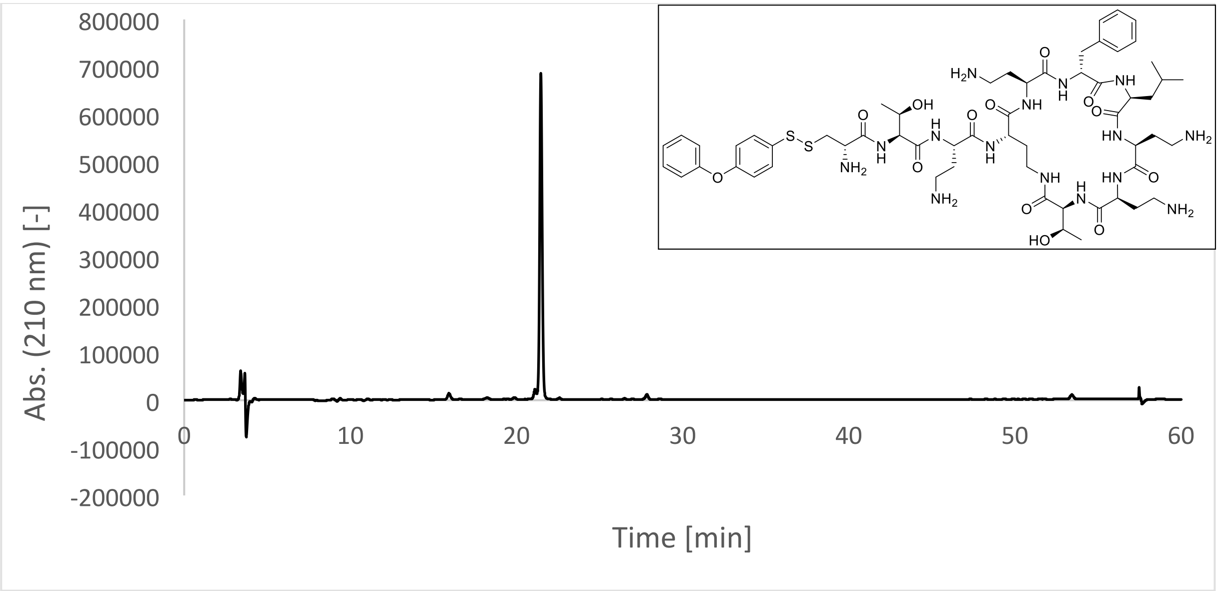

31a

Composition	Exact mass	M + H	(M+2H)/2	Found value
$C_{60}H_{90}N_{16}O_{13}S_2$	1306.6315	1307.6395	654.3238	1307.6397

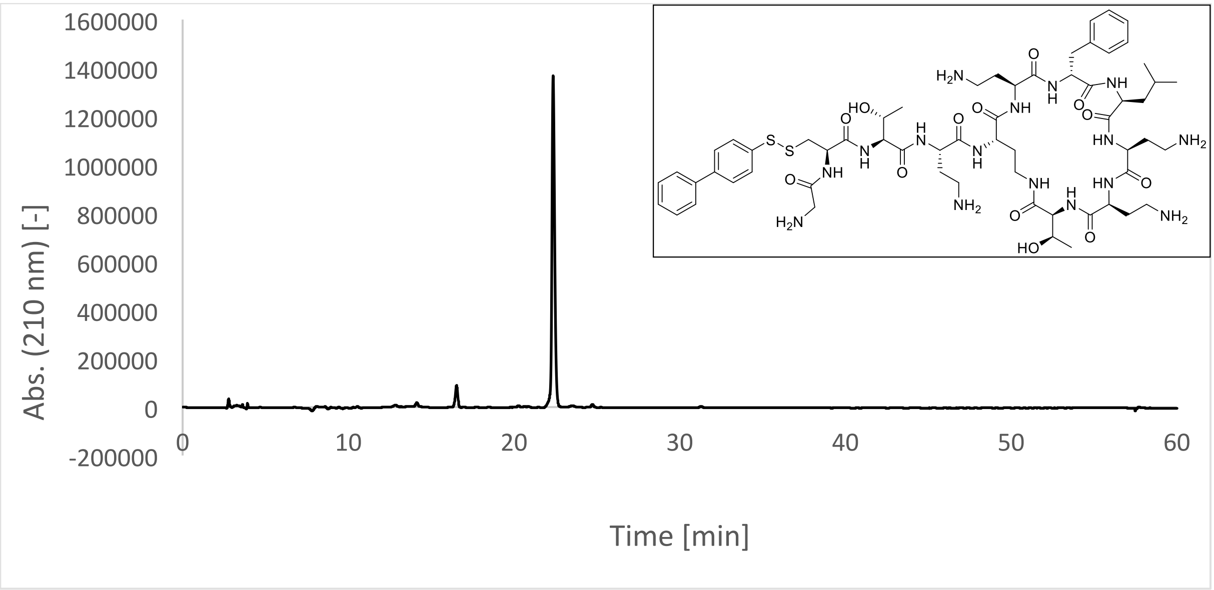

31b

Composition	Exact mass	M + H	(M+2H)/2	Found value
$C_{60}H_{90}N_{16}O_{13}S_2$	1306.6315	1307.6395	654.3238	654.3228

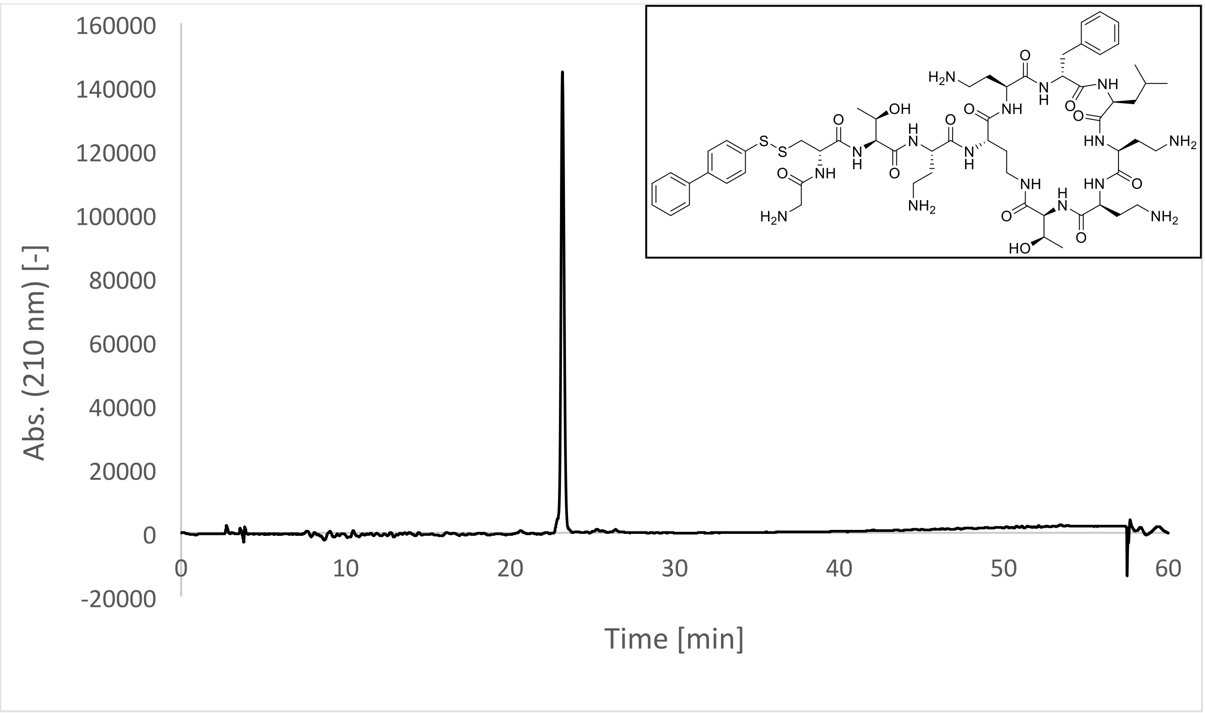

32a

Composition	Exact mass	M + H	(M+2H)/2	Found value
$C_{60}H_{90}N_{16}O_{14}S_2$	1322.6264	1323.6344	662.3212	1323.6350

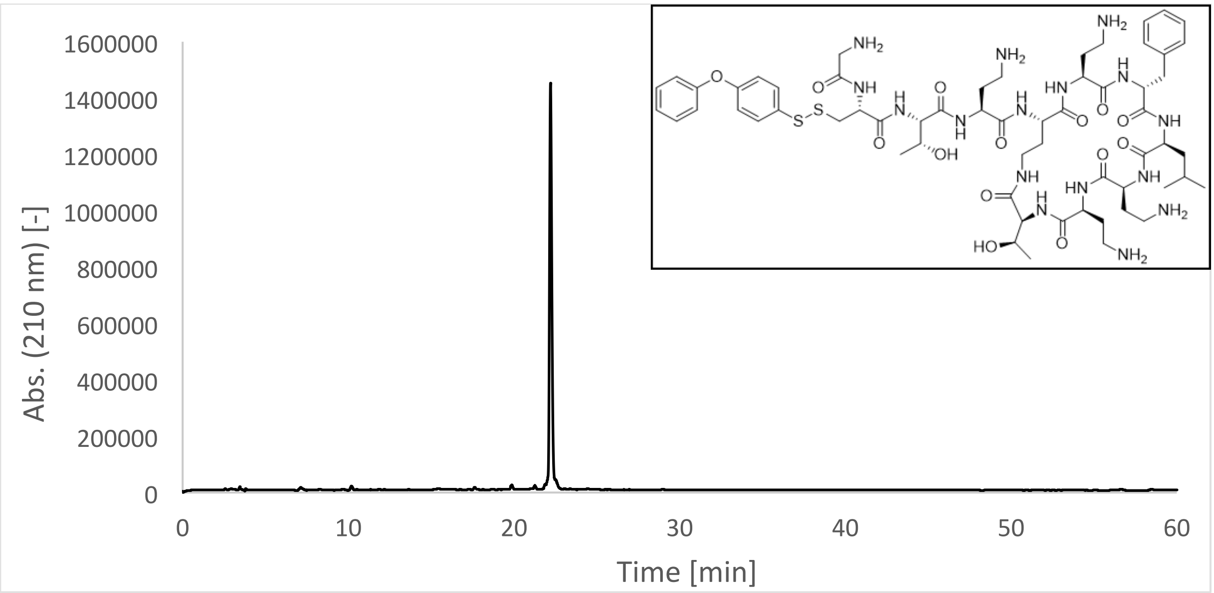

32b

Composition	Exact mass	M + H	(M+2H)/2	Found value
$C_{60}H_{90}N_{16}O_{14}S_2$	1322.6264	1323.6344	662.3212	662.3205

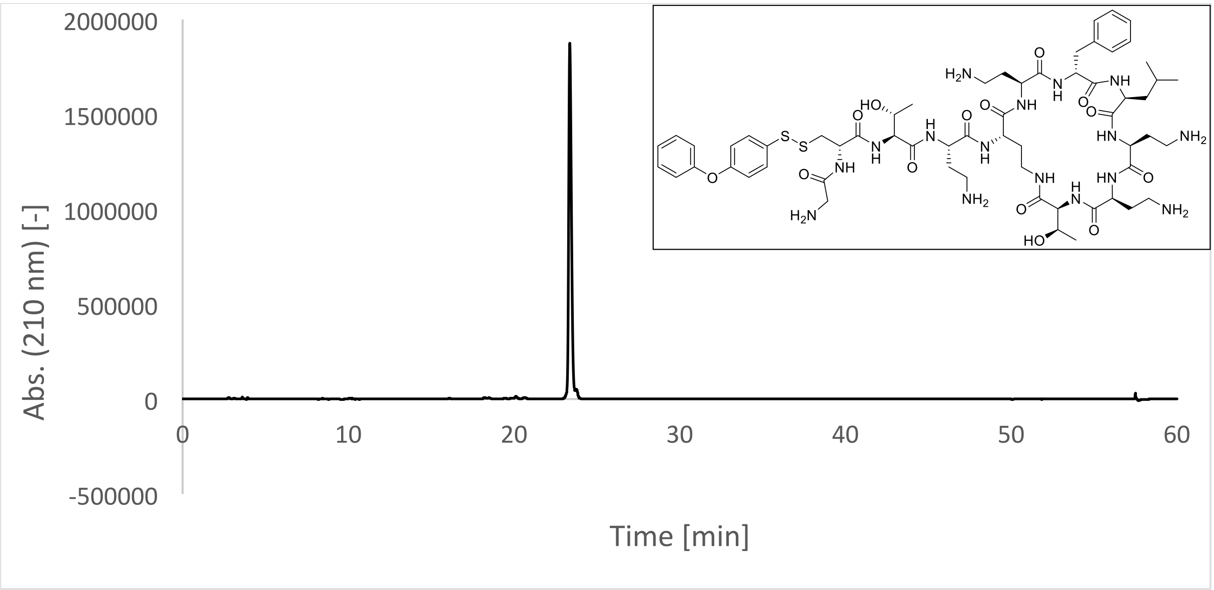

36

Composition	Exact mass	M + H	(M+2H)/2	Found value
$C_{59}H_{89}N_{15}O_{12}S_2$	1263.6257	1264.6337	632.8209	632.8209

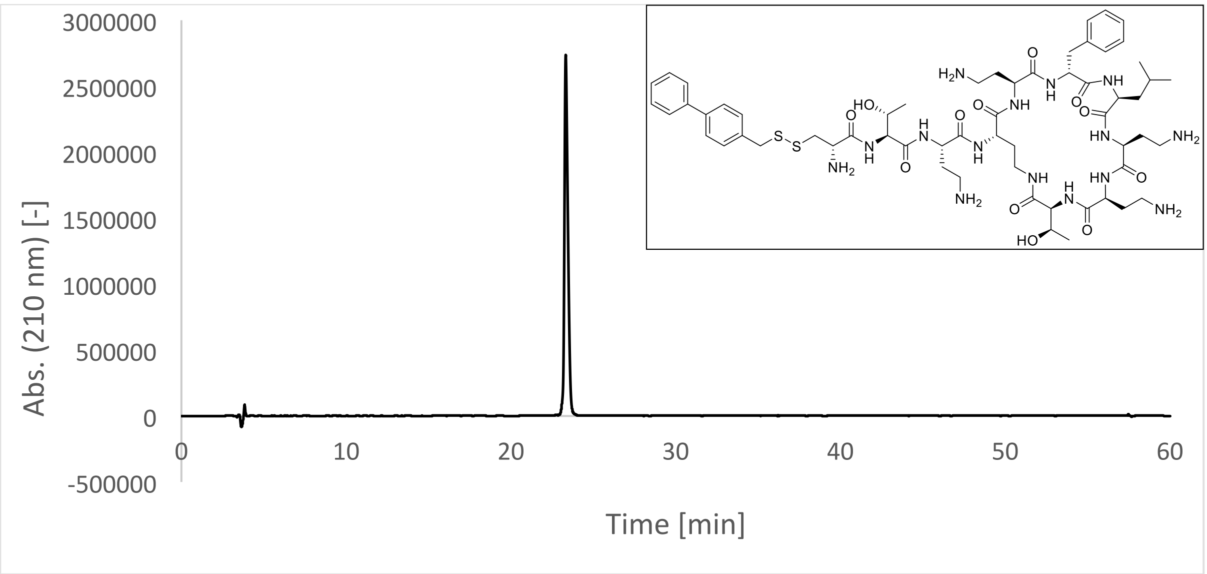

37

Composition	Exact mass	M + H	(M+2H)/2	Found value
$C_{59}H_{89}N_{15}O_{13}S_2$	1279.6206	1280.6286	640.8183	640.8179

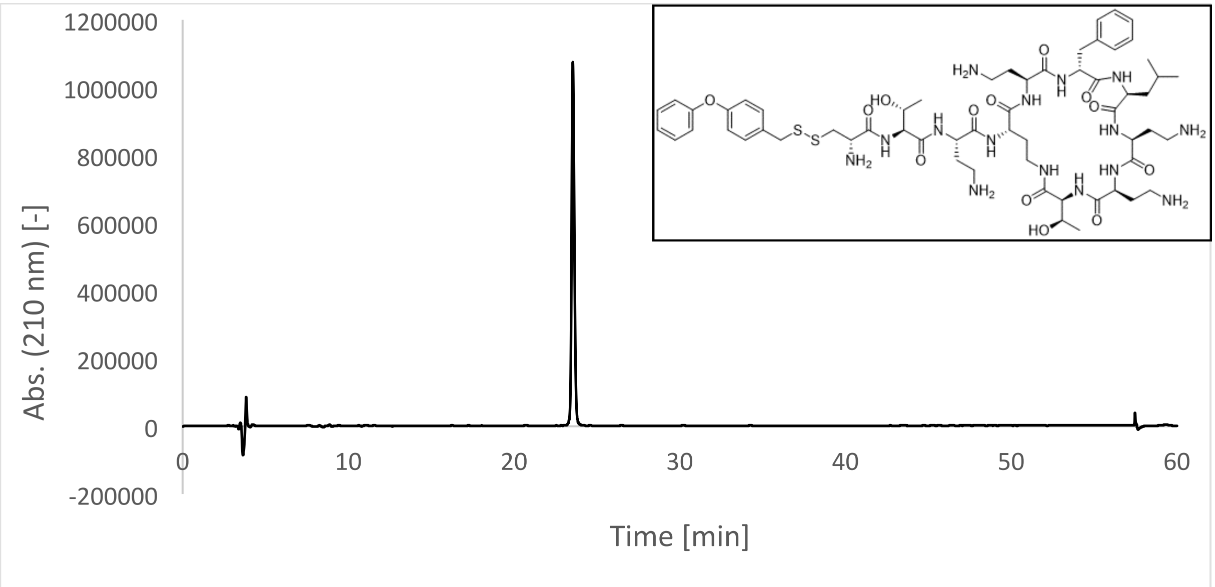

38

Composition	Exact mass	M + H	(M+2H)/2	Found value
$C_{53}H_{84}ClN_{15}O_{12}S_2$	1221.5554	1222.5634	611.7857	611.7855

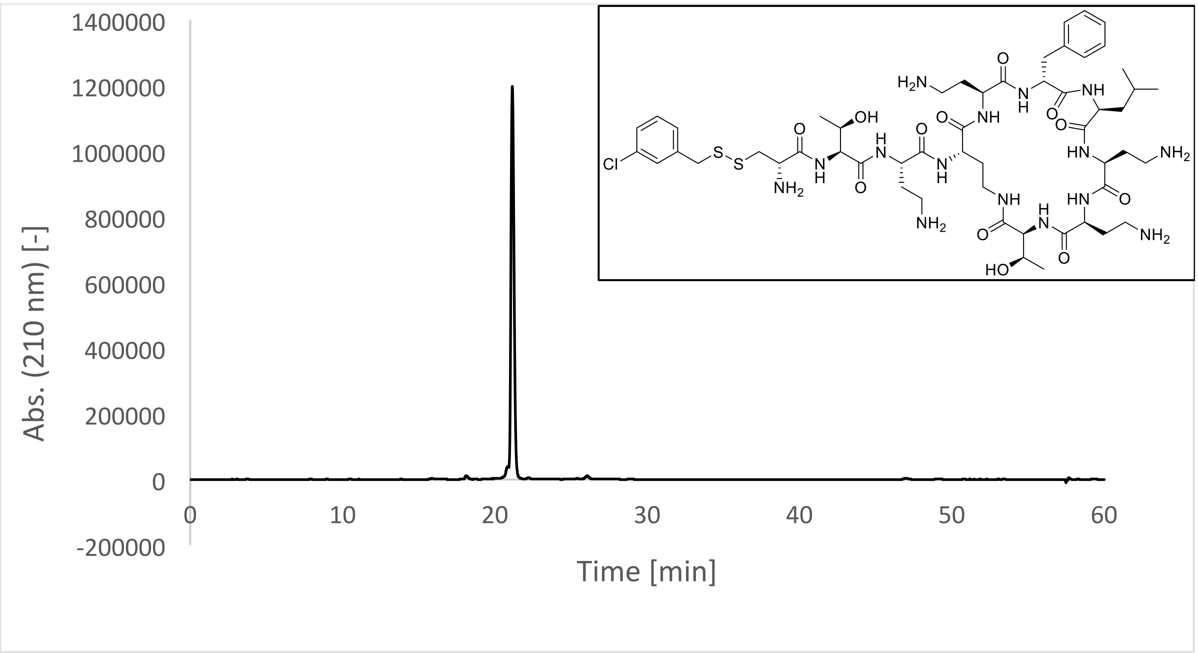

PMBN

Composition	Exact mass	M + H	(M+2H)/2	Found value
$C_{43}H_{74}N_{14}O_{11}$	962.5661	963.5741	482.2911	963.5738

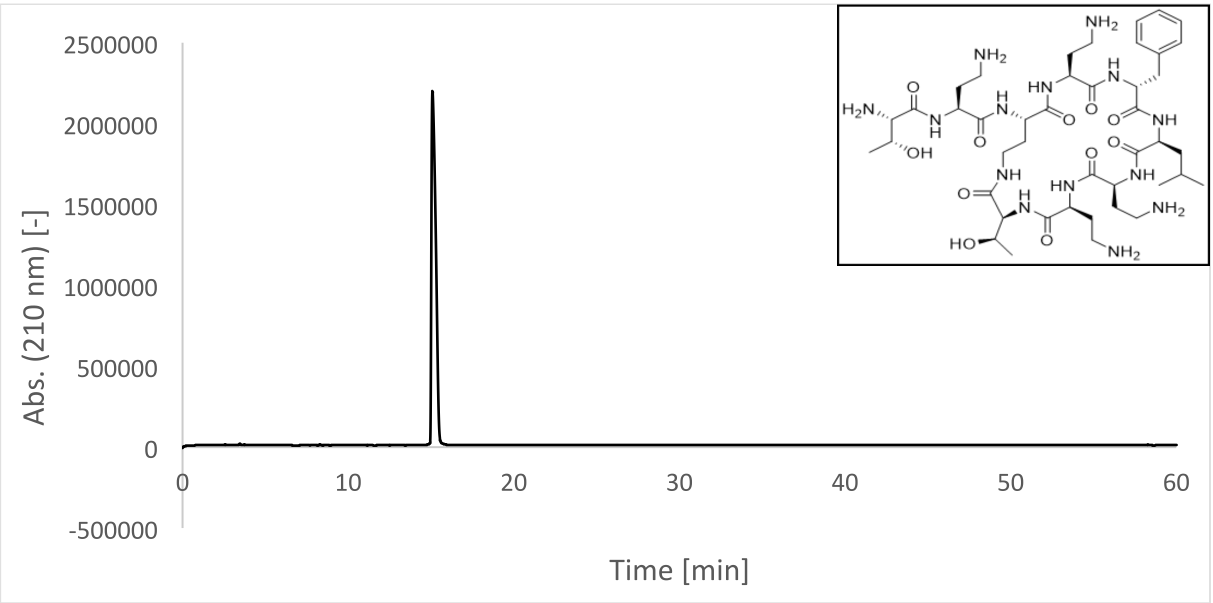

45

Composition	Exact mass	M + H	(M+2H)/2	Found value
$C_{53}H_{93}N_{15}O_{12}$	1131.7128	1132.7208	566.8644	566.8643

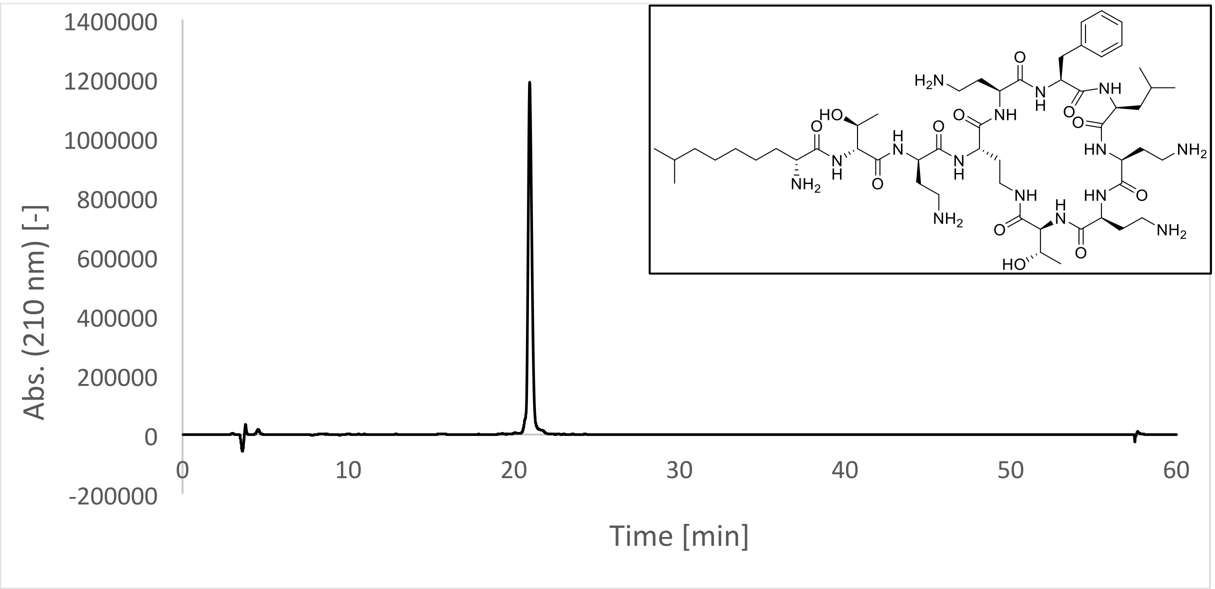

Stability assessment in presence of glutathione

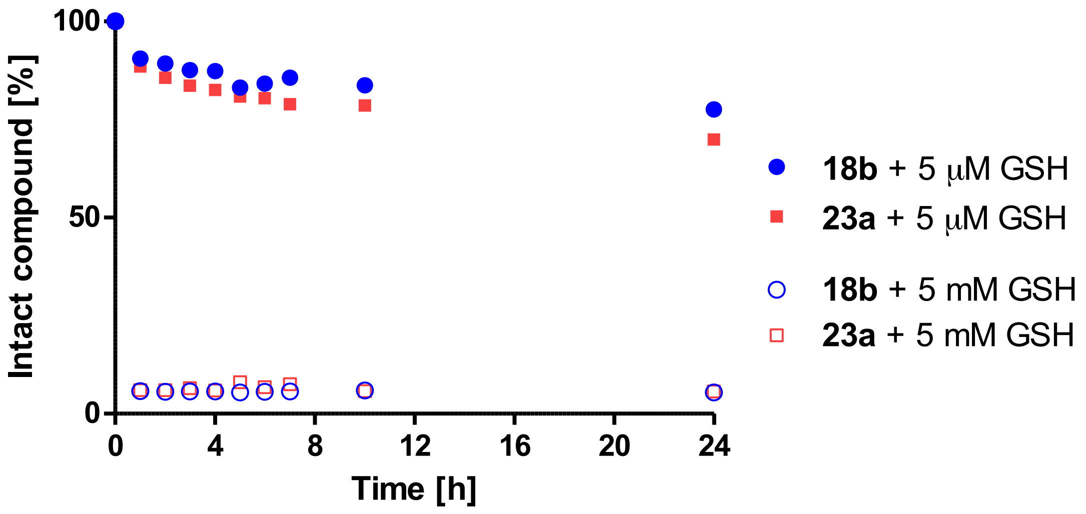

Figure S1. *Compound stability upon incubation with low (5 μM) or high (5 mM) glutathione (GSH) concentration. Compounds were incubated in PBS with the indicated concentration glutathione for 24 hours. Indicated data points are based upon integration of HPLC UV data.*

Hemolysis assessment

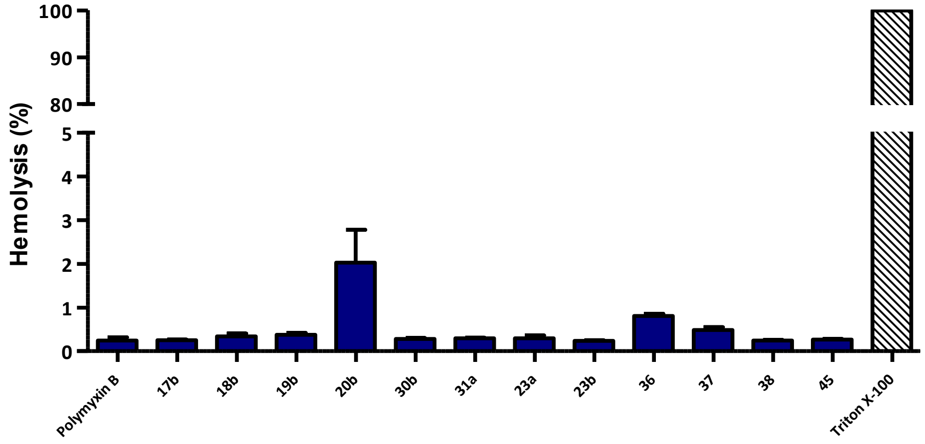

Figure S2. *Hemolysis data on best performing analogues, tested at 128 μg/mL. All tested compounds are not hemolytic at 128 μg/mL as the percent hemolysis is below 5%. Data are based on triplicates and normalized based on the hemolysis observed for 0.1% Triton-X100.*

Toxicity assessment on PTECs

Table S1. *TC_{50} values for polymyxin analogues. Toxicity assessment on proximal tubular epithelial cells performed using PrestoBlue™ assay. TC_{50} values are derived after non-linear regression analysis on cell viability data, with bottom constraint at 0 (see representative curves provided in Figure S3 below). Day 1 and Day 2 correspond to data obtained from 2 different batches of cells/analyses on different days. As the value for polymyxin B differs 2-fold, values in the manuscript are presented relative to the polymyxin B value.*

Day 1	
**Compound**	**TC_{50} (μM)**
**Polymyxin B**	80
**17a**	421
**17b**	240
**18a**	786
**18b**	345
**21b**	357
**22a**	305
**22b**	163

Day 2	
**Compound**	**TC_{50} (μM)**
**Polymyxin B**	41
**18b**	192
**19b**	51
**20b**	135
**23a**	391
**23b**	58
**24a**	327
**24b**	63
**36**	68
**37**	72
**38**	74
**45**	82

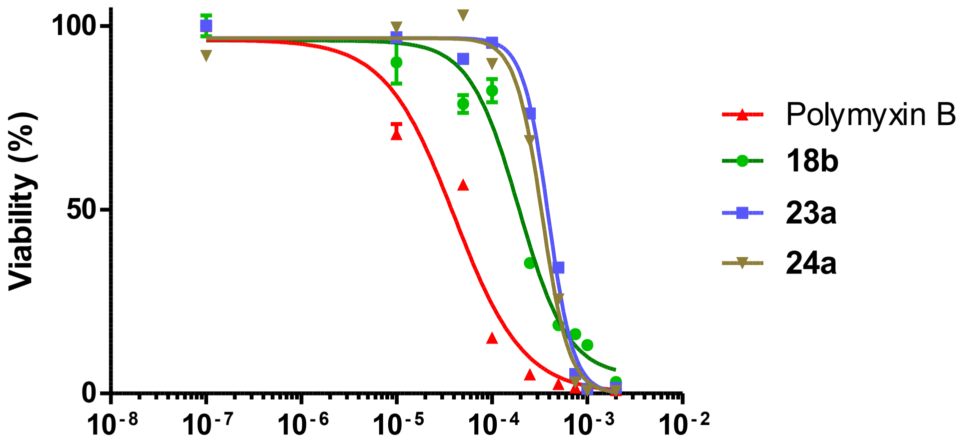

Figure S3. *Representative traces of ciPTECs' viability after incubation with analogues for 24 hours. Shown data are based on triplicates.*

Bacterial strains used for MIC assays and extended MIC assessment

Strains: The following strains were obtained from BEI Resources, NIAID, NIH: *E. Coli* M0072, strain MVAST0072, NR-51488; K. Pneumoniae, KP1.53, strain 1.53, NR-48978.

Table S2. *Extended MIC data on polymyxin B and analogues* ***18b, 23a****, and* ***24a****. Values are given in µg/mL.*

Species	Strain ID	Resistance indicator	Polymyxin B	18b	23a	24a
E. coli	M0072	Multiple	0.125	0.5	0.5	1
	1793	NDM-1	0.063	0.25	0.5	1
	JS136	OXA-48	0.125	0.5	0.5	1
K. pneumoniae	RC0045	OXA-48	0.25	0.5	1	1
	KP1.53	MDR	0.5	1	2	2
	ATCC 29665	-	0.25	0.5	1	1
A. baumannii	NRZ-00687	NDM-2	0.25	1	0.5	1
	RUH-134	Multiple	0.25	1	1	1
	BAA-747	-	0.25	0.5	1	1
P. aeruginosa	2018-007	IMP-7	2	2	8	16
	M120	OXA-50, blaPAO	1	2	2	2
	2251	VIM-2	1	2	2	2
	ATCC 10145	-	1	2	2	4

Table S3. *MIC values [µg/mL] and relative toxicity values for disulfide containing polymyxin derivative* ***18b*** *and non-disulfide containing analogue* ***45****.*

General Structure:

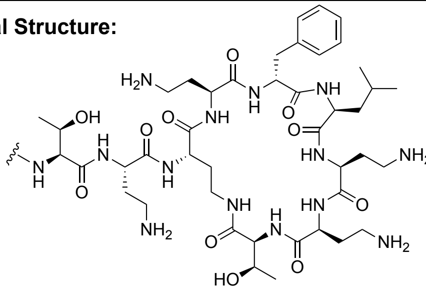

Compound (structural features indicated)

	Strain	Polymyxin B 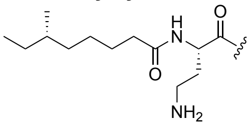	18b 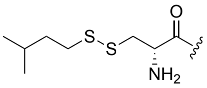	45 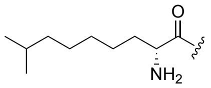
**MIC (µg/mL)**	*E. coli* (ATCC 25922)	1	1	1
	K. pneumoniae (ATCC13883)	0.25	0.5	≤0.25
	A. baumanii (ATCC 17961)	0.25	0.25	0.25
	A. baumanii (NRZ 00687)	0.25	1	0.5
	A. baumannii (ATCC 19606)	0.25	1	0.25
	P. aeruginosa (PAO1)	0.5	1	1
	P. aeruginosa (ATCC 27853)	1	1	1
	**ciPTEC TC_{50} (µM)**	41	192	82
	**ciPTEC Toxicity Index (relative to polymyxin B)**	1.0	4.7	2.0

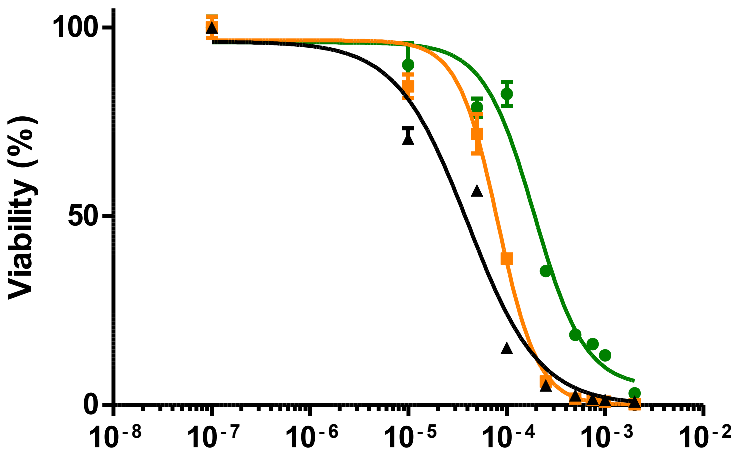

Figure S4. *ciPTEC viability after incubation with disulfide analogue* ***18b*** *and the corresponding analogue without disulfide bond* ***45****. Data shown are based on triplicates.*

References

(1) Zheng, Z.; Li, G.; Wu, C.; Zhang, M.; Zhao, Y.; Liang, G. Intracellular Synthesis of D-Aminoluciferin for Bioluminescence Generation. *Chem. Commun.* **2017**, *53* (25), 3567–3570.

(2) Jiang, Y.; Qin, Y.; Xie, S.; Zhang, X.; Dong, J.; Ma, D. A General and Efficient Approach to Aryl Thiols: CuI-Catalyzed Coupling of Aryl Iodides with Sulfur and Subsequent Reduction. *Org. Lett.* **2009**, *11* (22), 5250–5253.

(3) Balakumar, A.; Lysenko, A. B.; Carcel, C.; Malinovskii, V. L.; Gryko, D. T.; Schweikart, K.-H.; Loewe, R. S.; Yasseri, A. A.; Liu, Z.; Bocian, D. F.; Lindsey, J. S. Diverse Redox-Active Molecules Bearing O-, S-, or Se-Terminated Tethers for Attachment to Silicon in Studies of Molecular Information Storage. *J. Org. Chem.* **2004**, *69* (5), 1435–1443.

(4) Szajnman, S. H.; Yan, W.; Bailey, B. N.; Docampo, R.; Elhalem, E.; Rodriguez, J. B. Design and Synthesis of Aryloxyethyl Thiocyanate Derivatives as Potent Inhibitors of Trypanosoma Cruzi Proliferation. *J. Med. Chem.* **2000**, *43* (9), 1826–1840.

(5) Masakuni, K.; Kazumasa, H.; Hiromitsu, F.; Yamamoto, T. Aminoethanol Derivatives. Patent application EP1362846, 2003.

(6) Kokotos, G.; Padrón, J. M.; Martín, T.; Gibbons, W. A.; Martín, V. S. A General Approach to the Asymmetric Synthesis of Unsaturated Lipidic α-Amino Acids. The First Synthesis of α-Aminoarachidonic Acid. *J. Org. Chem.* **1998**, *63* (11), 3741–3744.
